# Feedback Inhibition of Bacterial Nucleotidyltransferases by Rare Nucleotide l-Sugars Restricts Substrate Promiscuity

**DOI:** 10.1021/jacs.3c02319

**Published:** 2023-06-07

**Authors:** Meng Zheng, Maggie C. Zheng, Hanee Kim, Tania J. Lupoli

**Affiliations:** Department of Chemistry, New York University, New York, New York 10003, United States

## Abstract

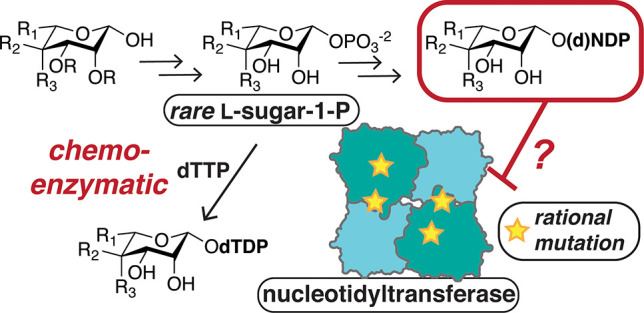

Bacterial glycomes are rich in prokaryote-specific or “rare” sugars that are absent in mammals. Like common sugars found across organisms, rare sugars are typically activated as nucleoside diphosphate sugars (NDP-sugars) by nucleotidyltransferases. In bacteria, the nucleotidyltransferase RmlA initiates the production of several rare NDP-sugars, which in turn regulate downstream glycan assembly through feedback inhibition of RmlA via binding to an allosteric site. *In vitro*, RmlA activates a range of common sugar-1-phosphates to produce NDP-sugars for biochemical and synthetic applications. However, our ability to probe bacterial glycan biosynthesis is hindered by limited chemoenzymatic access to rare NDP-sugars. We postulate that natural feedback mechanisms impact nucleotidyltransferase utility. Here, we use synthetic rare NDP-sugars to identify structural features required for regulation of RmlA from diverse bacterial species. We find that mutation of RmlA to eliminate allosteric binding of an abundant rare NDP-sugar facilitates the activation of noncanonical rare sugar-1-phosphate substrates, as products no longer affect turnover. In addition to promoting an understanding of nucleotidyltransferase regulation by metabolites, this work provides new routes to access rare sugar substrates for the study of important bacteria-specific glycan pathways.

Bacterial cell surfaces are composed of polysaccharides and glycoconjugates that mediate cellular interactions and form a protective barrier, such as the Gram-negative cell envelope (Figure 1A).^1,2^ These glycan structures often differ in composition from those of eukaryotes, as bacteria produce prokaryote-specific or “rare” sugars that are absent in mammals.^3−6^l-Rhamnose (l-Rha) is one of the most prevalent prokaryote-specific sugars.^1,3,7^ An activated form of l-Rha, dTDP-β-l-Rha (**1**) (dTDP = deoxythymidine diphosphate), is synthesized via a four-step biosynthetic pathway that begins with the coupling of α-d-glucose-1-phosphate (α-Glc-1P) and deoxythymidine triphosphate (dTTP) by the nucleotidyltransferase RmlA (Figure 1B).^8^ Intermediates from this pathway can be used to form another activated 6-deoxy rare sugar called dTDP-6-deoxy-β-l-talose (dTDP-β-l-6dTal, **2**).^9−11^ RmlA serves as a point of regulation for bacterial glycan biosynthesis through feedback inhibition by dTDP-β-l-Rha and possibly other nucleotide sugars.^12,13^

**Figure 1:**
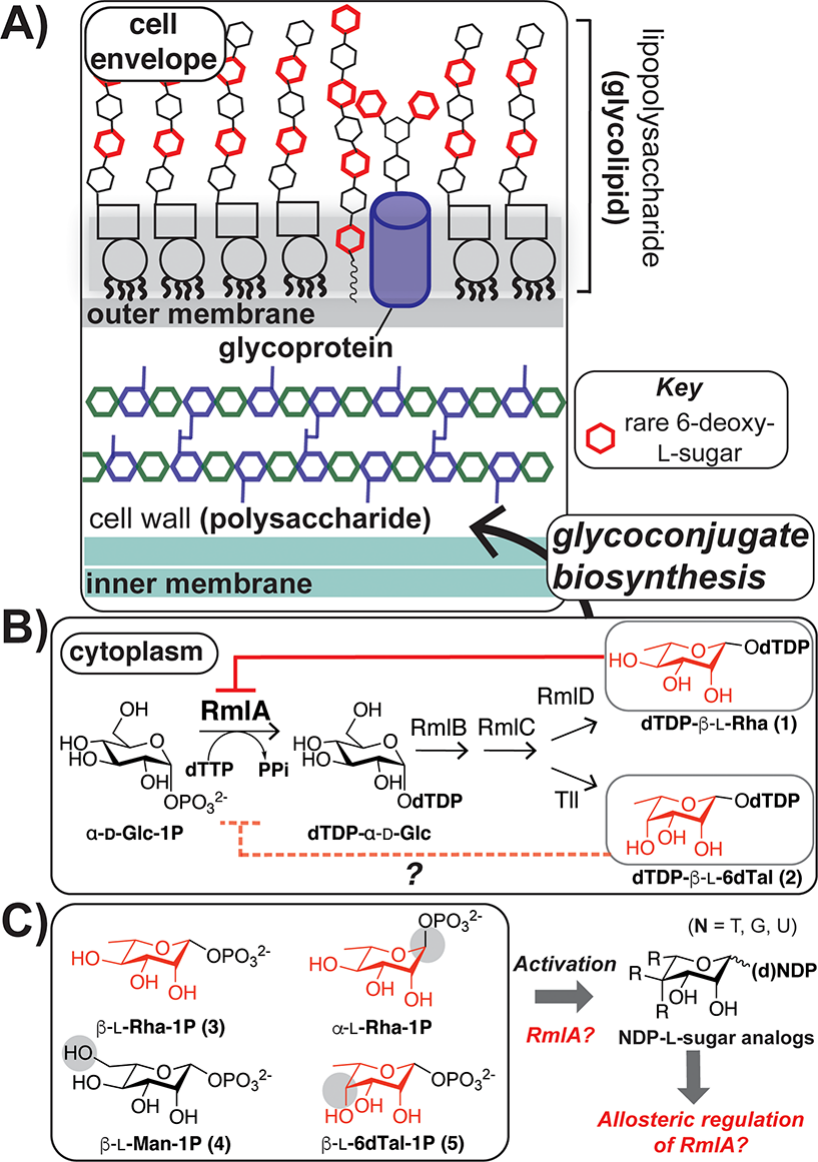
l-6-Deoxysugars absent in mammals are enriched in bacterial glycans. (A) Schematic of Gram-negative cell envelope with key glycans shown; prokaryote-specific (“rare”) sugars are shown in red. (B) Representative *E. coli* pathway for rare l-6-deoxysugar biosynthesis by the nucleotidyltransferase RmlA, which is feedback-regulated by dTDP-β-l-Rha and possibly other dNDP-sugars. (C) Overview of goals for this study.

Bacterial monosaccharides are enriched in other 6-deoxy-l-sugars^3^ that lack a hydroxyl at the C6-position and are l as opposed to the d stereochemistry found in most common sugars. Rare nucleotide sugars that contain β-anomeric centers are challenging to access synthetically, perhaps due to stability issues.^14−22^ While a recent one-pot, multistep enzymatic strategy produces nucleotide rare sugars from simple monosaccharides, these routes require several proteins, some of which suffer from product inhibition.^23,24^ Similarly, nucleotidyltransferases like RmlA are often modulated by intermediates that bind to an allosteric site to control flux.^25−29^ Despite several studies on RmlA,^13,25,26,30,31^^,^ the structural features of nucleotide sugars involved in allosteric recognition are still unknown, which restricts our ability to devise new strategies to rare nucleotide sugars via nucleotidyltransferase activity and impedes the study of bacterial glycan biosynthetic pathways that represent potential antibiotic and antivirulence targets.^13,32−34^

Here, we synthesize rare nucleotide sugar analogues to examine structural features that are required for allosteric regulation of RmlA. Since we have shown that mutation of the allosteric pocket of RmlA affects substrate selectivity at the active site,^35^ we now evaluate if mutated RmlA can produce rare deoxynucleoside diphosphate (dNDP)-sugars that typically inhibit RmlA. We find that **1** is the most potent regulator of RmlA across species and that RmlA mutants insensitive to feedback inhibition can activate various l-sugar substrates. In addition to promoting an understanding of nucleotidyltransferase regulation, which ultimately controls surface glycan assembly, this work provides alternative routes to nucleotide l-sugars.

NDP-sugars are often obtained via chemoenzymatic or synthetic activation of sugar-1-phosphates (S-1Ps) with appropriate stereochemistry. We aimed to obtain β-l-Rha-1P (**3**) and select S-1P analogues that could be activated to generate a collection of nucleotide sugars for structure–function studies (Figure 1C). To do so, we targeted the synthesis of **3** and the analogues β-l-mannose-1P (b-l-Man-1P, **4**), α-l-Rha-1P, and β-l-6dTal-1P (**5**). These S-1Ps would allow us to evaluate the consequences of hydroxylation of the C6-position and altered anomeric or hydroxyl stereochemistry on resulting nucleotide sugar function. Additionally, α-l-Rha and β-l-6dTal are present in bacterial nucleotide sugars^7,11,21^ that could potentially impact cellular nucleotidyltransferase activity. Inspired by stereoselective syntheses of α- or β-l-Rha-1P using temperature-controlled phosphorylation,^14,36−39^ we prepared partially acetylated precursors for l-Rha (**6a**), l-Man (**6b**), and l-6dTal (**6c**). **6a** and **6b** were obtained from commercial l-Rha and l-Man, respectively, following acetylation and benzylamine-mediated selective deprotection of the anomeric position (Figure 2A).^40,41^ For the preparation of **6c**, we began with anomeric *O*-allylation of l-Rha under thermodynamic control to afford the α-anomer, likely due to the anomeric effect (Figure 2B).^42,43^ Isopropylidene protection at C2 and C3 resulted in **7**.^44^ Then, C4 epimerization via Swern oxidation followed by hydride reduction from the less hindered face gave **8**.^45^ Finally, acetylation and anomeric deprotection afforded **6c**. A recent one-step sugar epimerization reaction^46^ has not yet been reported as compatible with protecting groups required for downstream phosphorylation.

**Figure 2:**
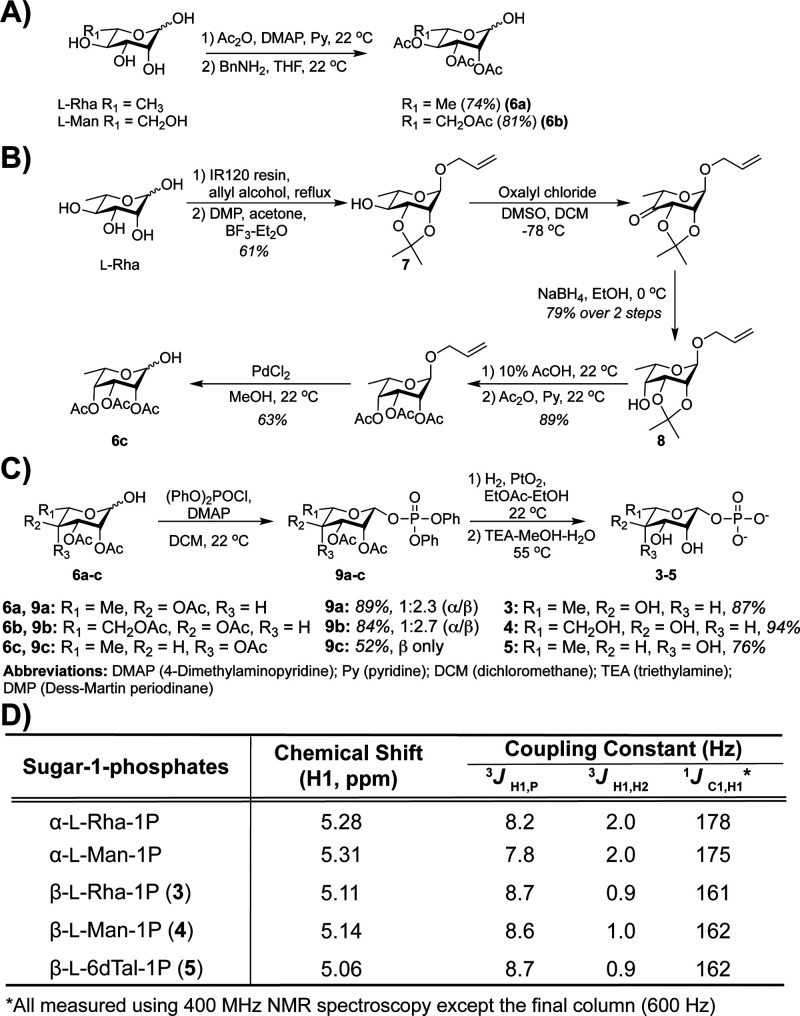
Rare l-S-1Ps can be accessed synthetically. (A, B) Schemes for partially acetylated l-Rha and analogues, followed by (C) phosphorylation and deprotection. (D) NMR characterization verifies isolation of indicated l-S-1Ps.

To produce desired S-1Ps, acetylated l-Rha, l-Man, and l-6dTal (**6a**–**6c**) precursors were then phosphorylated (Figure 2C). For each sugar, diphenyl chlorophosphate was added slowly at room temperature in the presence of excess base, which favored formation of β-products (**9a**–**9c**).^14^ Notably, we could never isolate α-l-6dTal-1P. Following mild deprotection to yield free S-1P-triethylammonium salts (**3**–**5**), we confirmed the anomeric configuration of each using ^1^H and ^13^C NMR (Figure 2D). Heteronuclear Single-Quantum Correlation Spectroscopy (HSQC) analyses of **3**–**5** provided ^1^*J*_C1,__H1_ coupling constants of ∼160 Hz for each, which is in line with the predicted value for β-l-Rha-1P.^19,47,48^ As a comparison, we isolated α-l-Rha-1P and α-l-Man-1P as byproducts of Figure 2C and observed ^1^*J*_C1,__H1_ coupling constants of >170 Hz. Using this approach, we achieved 5-step syntheses of **3** and **4** with overall yields of 33% and 49%, respectively. To our knowledge, this is the first reported synthesis of β-l-6dTal-1P (**5**), which was prepared in 10 steps with an overall yield of 11%.

To convert S-1Ps to nucleotide sugars, *N*,*N*-carbonyldiimidazole (CDI)-activated (deoxy)nucleoside monophosphates ((d)NMPs) have been coupled to S-1Ps to improve upon slower methods (*t* > 1 week).^19,49−52^ Since both l-Rha and l-6dTal are activated in bacteria as dTDP-β-l-sugars, we first produced dTMP-imidazolide (dTMP-Im) as a stable adduct to enable coupling to S-1Ps without requiring repeated *in situ* generation.^53^ For optimization of dTMP-Im and S-1P couplings (Figure 3A), the model substrates α-d-Glc-1P and dTMP-Im were evaluated with catalysts spanning a range of p*K*_a_ values (5–11),^54^ and reactions were monitored by ^31^P NMR (Figure S1). Of the nine catalysts tested, only *N*-methyl imidazole-HCl (NMI-HCl) led to formation of considerable dNDP-sugar (*t* = 18 h). Similar results have been observed in phosphate couplings between CDI-activated S-1Ps and lipids.^54^

**Figure 3:**
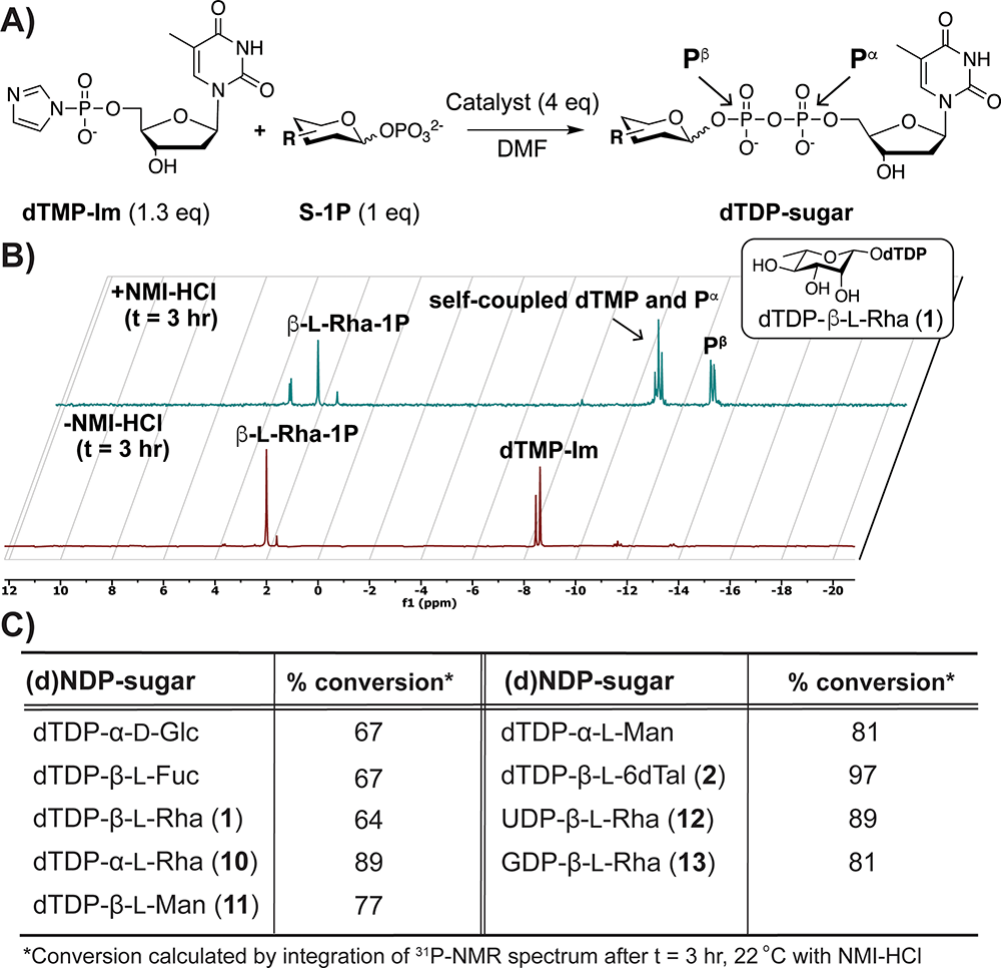
Formation of NDP-sugar analogues is accelerated by NMI-HCl. (A) General reaction scheme for asymmetric phosphate couplings. (B) ^31^P NMR analyses of β-l-Rha-1P (**3**) and dTMP-Im ± NMI-HCl demonstrates maximal coupling after *t* = 3 h, similar to other tested S-1Ps. Data is representative of *n* = 3. (C) Percent conversions of indicated products.

We then assessed the coupling of different l-S-1Ps to dNMP-Im reactants, as yields of nucleotide sugar syntheses typically vary widely.^19^ Over a short time course, a commercial l-deoxyhexose-1P, β-l-Fuc-1P, underwent nearly maximal nucleotide sugar formation at *t* = 3 h with NMI-HCl added, similar to α-Glc-1P (Figures S2 & S3). Coupling of β-l-Rha-1P (**3**) afforded **1** only in the presence of catalyst (Figures 3B & S4). Activation of the remaining synthetic l-S-1Ps (α-l-Rha-1P, α/β-l-Man-1P (**4**), β-l-6dTal-1P (**5**)) were similarly accomplished. Since uridine diphosphate (UDP) and guanosine diphosphate (GDP) are common activating groups for sugar metabolites,^21,55,56^**3** was also activated with appropriate NMP-Im substrates to generate additional NDP-sugar analogues for structure–function studies (Figure 1C). Despite the variety of reactants, >65% conversion was observed in each case (Figure 3C).

In bacterial NDP-sugar biosynthetic pathways, RmlA is feedback-inhibited by binding of **1** at a protein–protein interface between monomers, which associate to form the active tetramer (Figure 4A).^12^ We aimed to use our synthetic (d)NDP-l-sugars to define structural features necessary for RmlA regulation of both Gram-negative (*Escherichia coli*, *Pseudomonasaeruginosa* (Pa), *Salmonella enterica* LT2 (SALTY)) and Gram-positive, acid-fast (*Mycobacterium tuberculosis* (Mtb)) species, which have similar allosteric site structures (Figure S5). We reconstituted the activity of each RmlA to measure the half-maximal inhibitory concentration (IC_50_) of each nucleotide sugar (Figures 4A & S6). All tested RmlA homologues were inhibited by **1** in the mid- to high-micromolar range. Notably, the Hill slopes differed for each, with Mtb and SALTY RmlA tetramers producing the highest and lowest slope values, respectively (Figure 4B). These values reflect differing degrees of cooperativity upon ligand binding, as seen for Pa RmlA with different allosteric inhibitors.^13^ Fragments of **1** caused no RmlA inhibition (Figure S7), indicating that intact nucleotide sugars were required. Comparison to the α-anomer **10** resulted in a >7-fold IC_50_ increase across species. dTDP-β-l-Man (**11**), which contains a hydroxyl at the C6-position, behaved similarly. Inversion of stereochemistry at C4 in dTDP-β-l-6dTal (**2**) only showed inhibition against Pa RmlA. While thymidine analogues can inhibit RmlA,^13,30^ these results highlight the importance of the sugar in forming contacts with RmlA, as seen in solved cocomplexes (Figure 4C, left). Variation of the nucleotide in UDP-β-l-Rha (**12**) and GDP-β-l-Rha (**13**) led to loss of inhibitory activity, except for weak inhibition of Pa RmlA by **12**. Hence, NDPs, especially those with purines, are not tolerated in the dNDP binding site.

**Figure 4:**
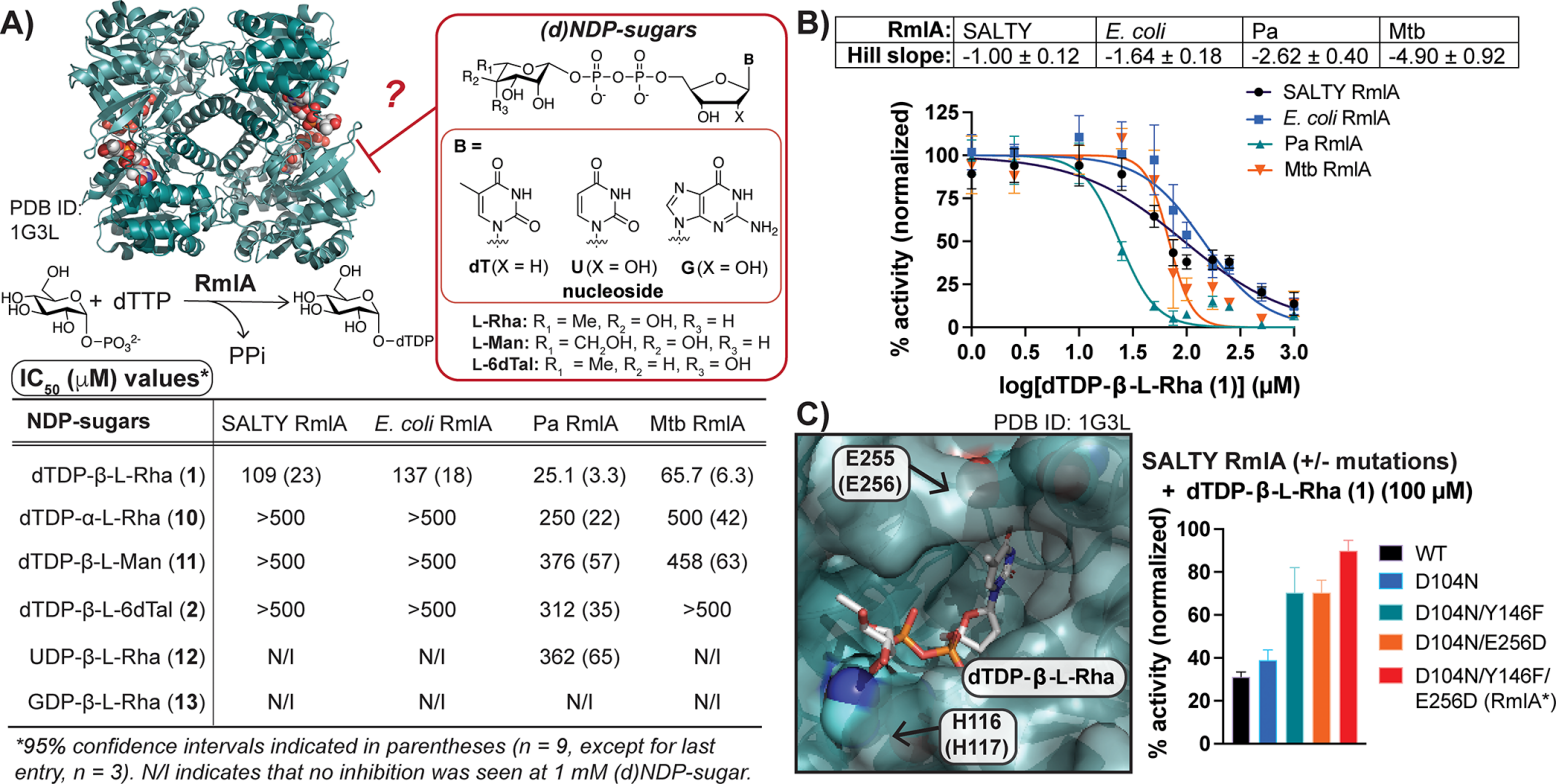
RmlA homologues are regulated by dTDP-l-sugars at an allosteric site. (A) IC_50_ analyses of nucleotide sugars added to indicated RmlA proteins. Structure of Pa RmlA bound to allosteric ligand (**1**) shown. (B) Representative IC_50_ curves indicate varying degrees of cooperativity with **1** added across species (*n* = 9). (C) *right:* SALTY RmlA demonstrates decreased sensitivity to **1** upon mutation (reactions normalized to those lacking inhibitor). *left:* Pa RmlA allosteric site (with corresponding SALTY RmlA residues) highlights important contacts with **1**. Standard deviation (SD) for all parts indicated by bars (*n* = 3).

Expanding on past work,^57−63^ we recently demonstrated that engineering of SALTY RmlA’s allosteric site leads to enhanced substrate promiscuity.^35^ Incorporation of the allosteric mutation E256D in a known active site mutant (Y146F) carrying the stabilizing alteration D104N also led to improvements in catalytic activity. As **1** tightly regulates all tested RmlA proteins and makes contacts with SALTY RmlA E256 (Figure 4C, left), we hypothesized that allosteric mutation might eliminate feedback inhibition. Accordingly, we found that while addition of **1** reduced wild-type activity to ∼30% of native levels, active or allosteric site point mutants maintained ∼70% activity in its presence (Figure 4C, right). The triple mutant (D104N/Y146F/E256D or RmlA*) was nearly insensitive to **1**. Kinetic analyses indicated that mutation of E256 leads to competitive inhibition by **1**, instead of the mixed inhibition mechanism observed against wild type^26^ (Figure S8). Hence, we postulated that engineered RmlA may activate l-S-1Ps without experiencing feedback inhibition by dNDP-l-sugar products.

With synthetic access to l-S-1Ps, we evaluated the substrate scope of engineered RmlA. As expected,^35^ incubation of RmlA* with α-Glc-1P, α-Man-1P, or β-l-Fuc-1P and dTTP (2 mM each) led to formation of the corresponding dTDP-sugars (Figure 5A). However, when the synthetic β-l-S-1Ps (**3**–**5**) or corresponding α-l-S-1Ps were incubated with dTTP under similar conditions, only β-l-Man-1P (**4**) was activated (Figure 5A,B), as confirmed by MS analysis (Figure 5C). Since some noncanonical S-1Ps exhibit high *K*_M_ values (>1 mM) with RmlA proteins,^35,63^ we next tested synthetic l-S-1Ps at a higher concentration (10 mM) and found that both β-l-Rha-1P (**3**) and β-l-6dTal-1P (**5**) could be activated by RmlA* using dTTP, as confirmed by LC-MS analysis of reactions and relevant standards (Figure 5D, traces i-ii, Figure S9). Under these conditions, α-l-Man-1P and only trace α-l-Rha-1P could be activated (traces iii-iv). Notably, the yield of dTDP-β-l-Man (**11**) was ∼10× higher than that of **1** (Figure S10). Wild-type enzyme does not promote activation of β-l-S-1P; even mutation of H117, which contacts the sugar on **1** (Figure 4C), results in low turnover of **4** (Figure S11). While RmlA* can activate α-Glc-1P with structurally diverse dNTPs and NTPs,^35^**4** (Figure S12) or **3** (Figure 5D, trace v) did not react with GTP or UTP, respectively, in RmlA* reactions. Hence, β-l-S-1Ps in the active site likely exclude the binding of non-native nucleotides in the adjacent binding cleft.

**Figure 5:**
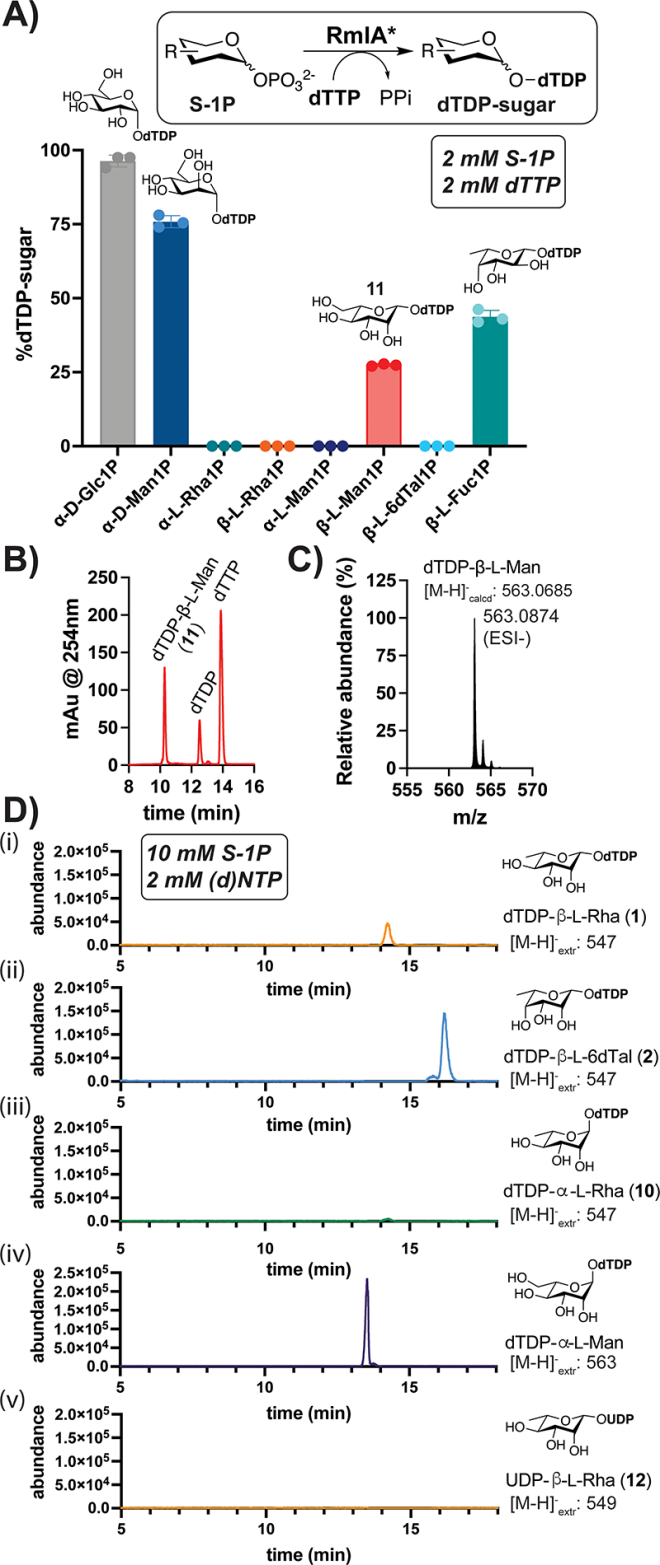
Engineered SALTY RmlA (RmlA*) activates l-S-1Ps. (A) Percent conversions of S-1Ps to indicated dTDP-sugars (*t* = 6 h). Error bars represent SD (*n* = 3). (B) HPLC and (C) HR-MS analyses of **11**. (D) LC-MS extracted ion chromatograms of RmlA* reactions indicate nucleotide sugar production at higher [substrate] (*n* = 3). RmlA* can produce most dTDP-sugars (traces i, ii, iv), but not a UDP-sugar (v) when UTP is added. [M-H]_extr_^−^ values indicate calculated *m*/*z* for indicated ions that were extracted.

In conclusion, we found that dTDP-β-l-Rha (**1**) is unique in its ability to inhibit RmlA proteins across species at mid-micromolar concentrations. As there is an estimated 20–600 μM of nucleotide sugars in bacteria, these inhibitory concentrations are physiologically relevant.^64^ Since dTDP-β-l-6dTal (**2**) is produced by a cellular pathway that relies on RmlA, these results suggest that direct product inhibition may dominate in some species,^26^ unless high micromolar concentrations are reached. Further, RmlA proteins from different species displayed distinct degrees of cooperativity. The observed differences between Mtb and Pa RmlA inhibition help rationalize the finding that a potent Pa RmlA allosteric inhibitor showed only weak activity against Mtb,^13^ even though *rmlA* is essential in Mtb.^65^ Hence, these findings may aid in the future design of species-selective inhibitors of RmlA.

Using engineered SALTY RmlA that is nearly insensitive to allosteric regulation, we demonstrated the first RmlA-mediated activation of rare β-l-S-1Ps. It has been hypothesized that RmlA might accept β-l-S-1Ps in the slightly higher-energy ^4^C_1_ conformation, which resembles the native α-Glc-1P substrate^63^ (Figure S13, Table S1). Alternatively, β-l-S-1Ps might assume a flipped ^1^C_4_ conformation in the active site, as observed when β-l-Man-1P was docked into SALTY RmlA (Figure S14). While not all substrates could be utilized, such as α-l-Rha-1P and GTP, dTDP-β-l-sugar production was likely enhanced due to lowered allosteric inhibition by the product as well as expanded substrate tolerance^35^ in the active site. Hence, allosteric regulation does restrict the substrate scope of this model nucleotidyltransferase. These findings serve as a foundation for improved chemoenzymatic syntheses of rare sugar substrates to study bacterial glycan assembly.

## Supporting Information Available

Tables S1–S5, Schemes S1–S3, and Figures S1–S14; description of experimental methods for sugar-1-phosphate and nucleotide sugar syntheses; compound characterization (NMR, mass spectrometry); methods for plasmid cloning, protein expression, and purification; and additional analyses of enzymatic activities of RmlA (HPLC and mass spectrometry analysis). Tables S2–S4 contain information on plasmids, primers, and strains that were utilized (PDF)
