# Supplementary material for: Feedback Inhibition of Bacterial Nucleotidyltransferases by Rare Nucleotide l-Sugars Restricts Substrate Promiscuity
Source: J Am Chem Soc. 2023 Jun 7;145(29):15632–8. doi: 10.1021/jacs.3c02319 (PMC10375476; doi:10.1021/jacs.3c02319)
Supplement: Supplementary file 1 — ja3c02319_si_001.pdf [file ja3c02319_si_001.pdf]

Supporting Information

Feedback inhibition of bacterial nucleotidyltransferases by rare nucleotide L-sugars restricts substrate promiscuity

Meng Zheng, Maggie C. Zheng, Hanee Kim, Tania J. Lupoli*

Department of Chemistry, New York University, New York, NY 10003,USA

*tjl229@nyu.edu

A)

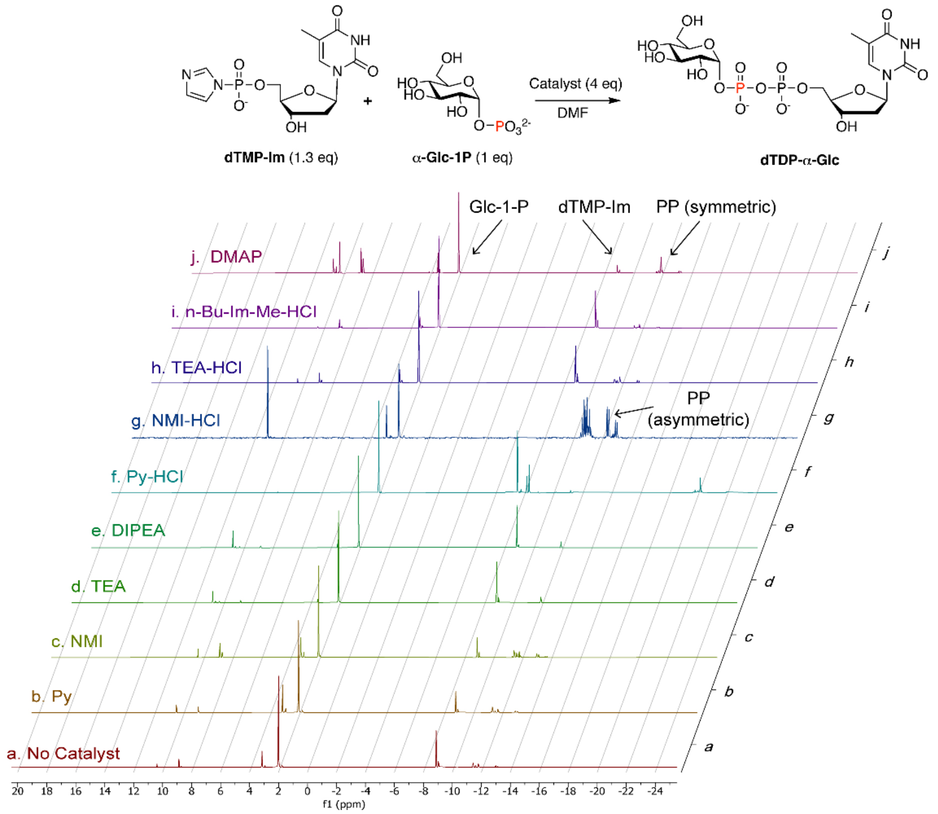

B) Estimated percent conversion* of S-1P to NDP-sugar.

Entry	Catalyst	pKa†	18 h	36 h	72 h	120 h
a	None	-	3.8	4.7	9.9	14.5
b	Pyridine (Py)	5.2	3.8	4.8	9.9	12.3
c	N-methylimidazole (NMI)	7.1	9.9	13.8	19.4	24.8
d	Triethylamine (TEA)	10.8	0	0	0	0
e	Diisopropylethylamine (DIPEA)	11.0	0	0	0	0
f	Py-HCl	5.2	0	0	0	0
g	NMI-HCl	7.1	62.6	66.0	66.2	70.0
h	TEA-HCl	10.8	3.8	7.4	12.8	17.0
i	1-n-Butyl-3-methylimidazolium chloride (n-Bu-Im-Me-HCl)	-	1.2	2.5	5.1	8.5
j	4-Dimethylaminopyridine (DMAP)	9.7	3.7	5.2	7.7	9.9

* Calculated using the following equation: $[P_\beta / (P_\beta + P_{S\text{-}1P}) \times 100\%]$

† pKa in H_2O, published elsewhere.[1, 2]

Figure S1. Time course of coupling reactions of Glc-1P with dTMP-Imidazolide to evaluate different catalysts. (a) Representative ^{31}P-NMR analyses of coupling reactions using α-Glc-1P and dTMP-Imidazolide (dTMP-Im) substrates with different catalysts indicates NMI-HCl is the best of tested catalysts at

promoting formation of the desired product (t = 18 hr) (PP = pyrophosphate). Full consumption of dTMP-Im (~ -9 ppm) to the desired asymmetric pyrophosphate bond was indicated by the appearance of two doublets (~ -11 to -14 ppm). Notably, addition of 4-dimethylaminopyridine (DMAP, trace J) led to the formation of a singlet (~ -12 ppm), which represents a symmetric pyrophosphate resulting from self-dimerization of dTMP, as confirmed by mass spectrometry.[3] See table in part B for abbreviations of catalysts tested. Catalysts were chosen based on a previous study on the optimization of phosphate condensation for glycolipid synthesis.[1] (B) Quantification of percent conversion to NDP-sugars using indicated catalysts as monitored by ^{31}P-NMR over a long time course (for t = 18 hr, traces shown in part A).

A)

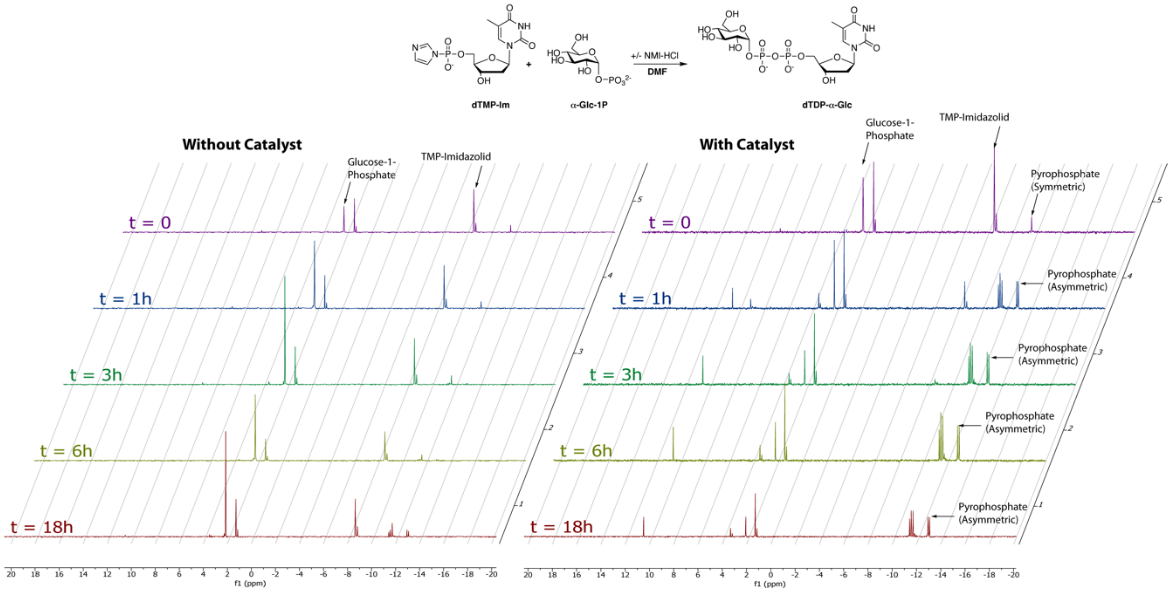

B) Estimated percent conversion* from S-1P to NDP-sugar

**Time**	**Without Catalyst**	**With Catalyst**
1 h	0	34.6
3 h	2.4	67.2
6 h	3.4	71.9
18 h	11.8	70.6

*Calculated as shown in **Figure S1B**.

Figure S2. Short time course of coupling reactions of Glc-1P with dTMP-Im -/+ NMI-HCl catalyst. (A) Representative ^{31}P-NMR analyses of coupling reactions using α-Glc-1P minus (left) or plus NMI-HCl (right) using the same reagent ratios as **Figure S1A** over the indicated time period, which demonstrates that NMI-HCl accelerates the reaction. With catalyst added, the reaction appears nearly complete within t = 3 hr. (B) Quantification of percent conversion to NDP-sugar based on data shown in part A.

A)

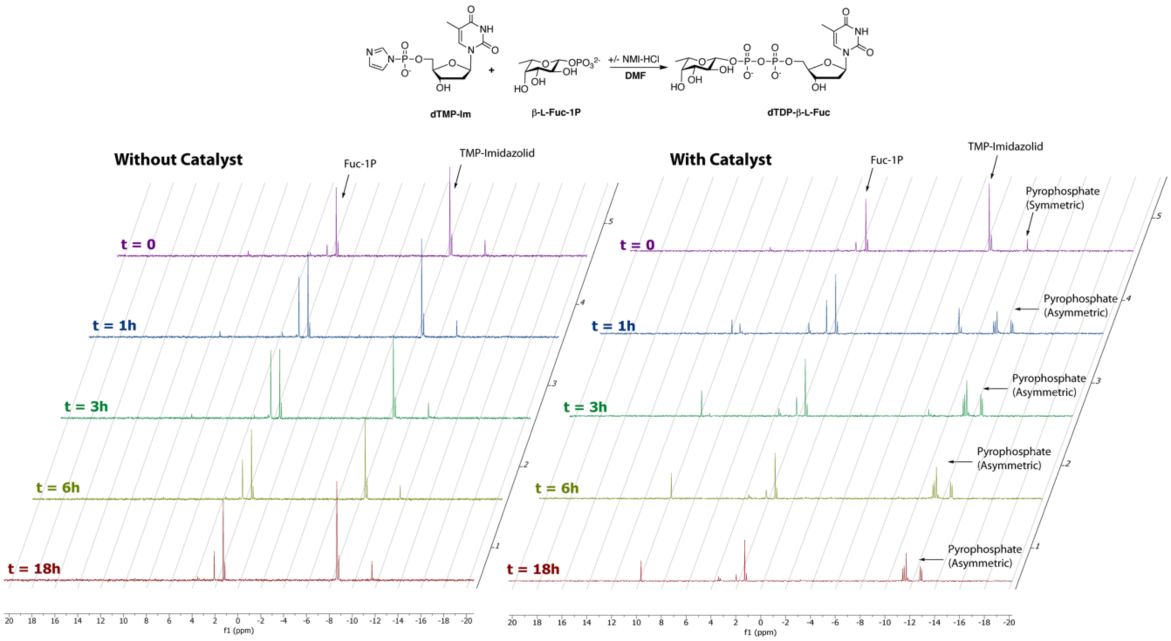

B) Estimated percent conversion* from S-1P to NDP-sugar

**Time**	**Without Catalyst**	**With Catalyst**
1 h	0	40.8
3 h	0	67.1
6 h	0	70.5
18 h	0	76.2

*Calculated as shown in **Figure S1B**.

Figure S3. Short time course of coupling reactions of β-L-Fuc-1P with dTMP-Im -/+ NMI-HCl catalyst. Representative ^{31}P-NMR analyses of coupling reactions using β-L-Fuc-1P minus (left) or plus NMI-HCl (right) using the same reagent ratios as **Figure S1A** over the indicated time periods demonstrate that NMI-HCl accelerates the reaction, and it is nearly complete in t = 3 hr with catalyst added. (B) Quantification of percent conversion to NDP-sugar based on data shown in part A.

A)

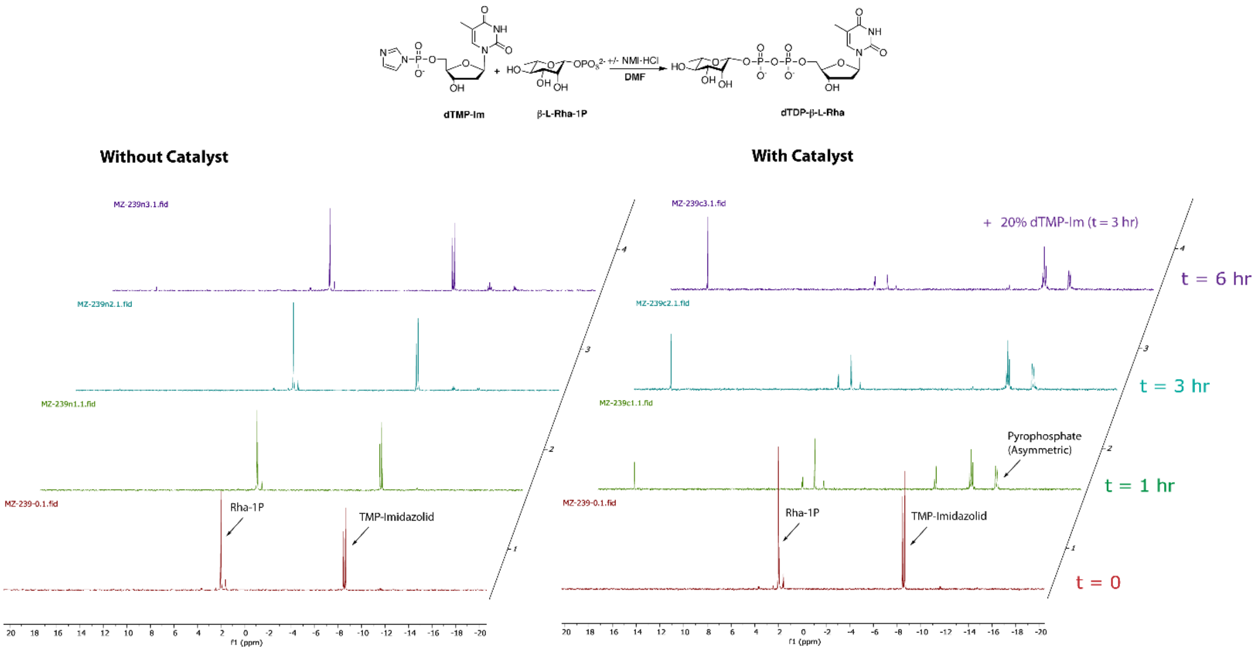

B) Estimated percent conversion* from S-1P to NDP-sugar

**Time**	**Without Catalyst**	**With Catalyst**
1 h	trace	53
3 h	3.6	64
6 h	10	76

*Calculated as shown in **Figure S1B**.

Figure S4. Short time course of coupling reactions of β-L-Rha-1P (3) with dTMP-Im -/+ NMI-HCl catalyst. (A) Representative ^{31}P-NMR analyses of coupling reactions using β-L-Rha-1P minus (left) or plus NMI-HCl (right) using the same reagent ratios as **Figure S1A** over the indicated time periods demonstrate that NMI-HCl accelerates the reaction, and it is nearly complete in t = 3 hr with catalyst added. Note that 20% additional dTMP-Im was added to the reaction with catalyst at t = 3 hr. (B) Quantification of percent conversion to NDP-sugar based on data shown in part A.

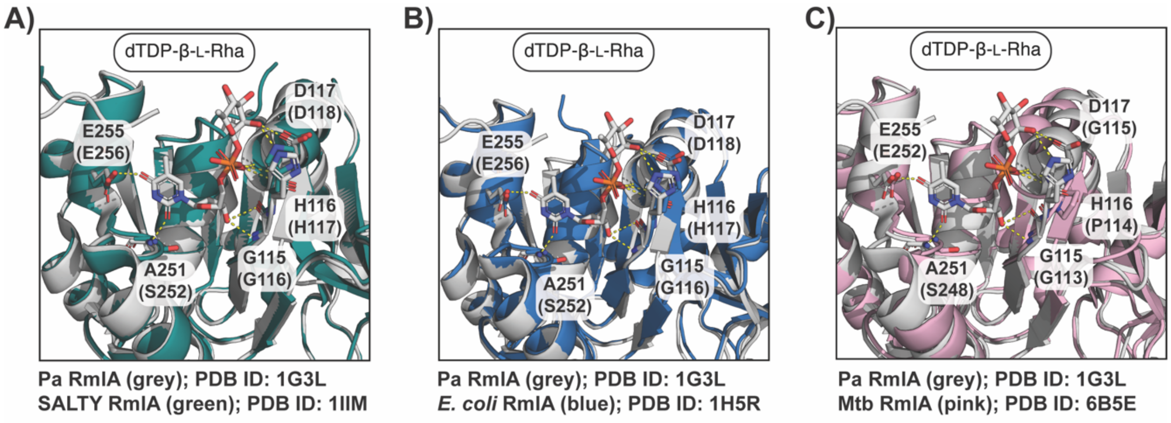

Figure S5. RmlA proteins from diverse species have similar allosteric binding clefts. Alignments of *P. aeruginosa* (Pa) RmlA monomer bound to dTDP-β-L-Rha with monomers from (A) *Salmonella enterica* LT2 (SALTY) RmlA (bound to dTTP, not shown) (RMSD 0.440); (B) *E. coli* RmlA (bound to thymidine and Glc-1P, not shown) (RMSD 0.427); and (C) *M. tuberculosis* (Mtb) RmlA (bound to dTDP-α-Glc, not shown) (RMSD 0.769). In each structural alignment, residues in parentheses indicate homologous residues from RmlA protein that is aligned to Pa RmlA/dTDP-β-L-Rha complex. While the allosteric sites are all similar, note that two helices are shifted in Mtb RmlA, but this structure is co-complexed with product, which might affect its conformation. The yellow dashed lines indicate polar contacts (H-bonds) made between Pa RmlA and ligand (Alignments and polar contact detection performed in PyMol (The PyMOL Molecular Graphics System, Version 2.0 Schrödinger, LLC).

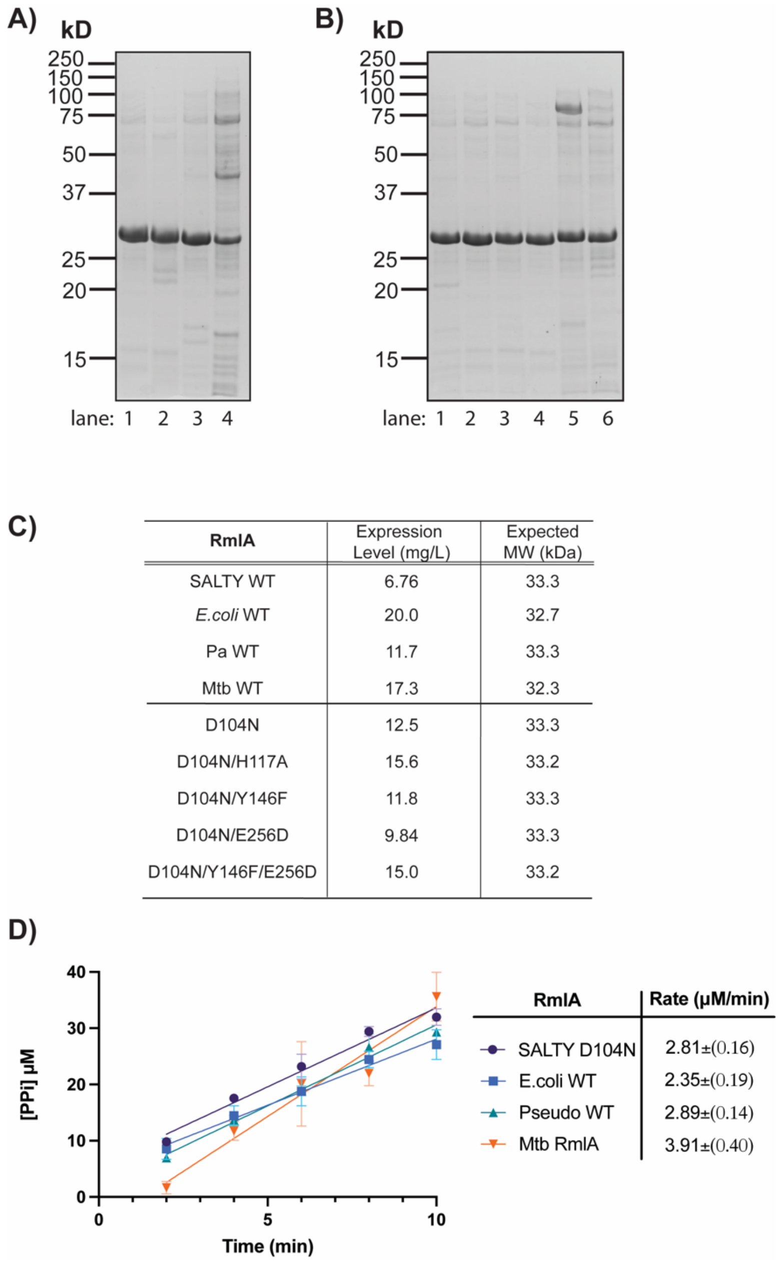

RmlA	Expression Level (mg/L)	Expected MW (kDa)
SALTY WT	6.76	33.3
E.coli WT	20.0	32.7
Pa WT	11.7	33.3
Mtb WT	17.3	32.3
D104N	12.5	33.3
D104N/H117A	15.6	33.2
D104N/Y146F	11.8	33.3
D104N/E256D	9.84	33.3
D104N/Y146F/E256D	15.0	33.2

RmlA	Rate (μM/min)
SALTY D104N	2.81±(0.16)
E.coli WT	2.35±(0.19)
Pseudo WT	2.89±(0.14)
Mtb RmlA	3.91±(0.40)

Figure S6. RmlA from different species could be purified and showed similar initial rates under specified conditions. (A) SDS-PAGE analyses of RmlA from various species. Lanes 1-4 represent the following enzymes: SALTY RmlA D104N, *E. coli* RmlA, Pa RmlA, and Mtb RmlA. 5 μg of each protein was loaded. (B) SDS-PAGE analyses of SALTY RmlA mutants. Lanes 1-6 represent the following enzymes: SALTY RmlA WT, D104N, D104N/H117A, D104N/Y146F, D104N/E256D, and D104N/Y146F/E256D. 5 μg of each protein was loaded. Higher molecular weight bands likely indicate oligomer formation. (C) Expression levels in mg/mL of each purified RmlA protein with expected molecular weight (MW) shown. (D) Rate analysis was carried out with 12.5 nM each of SALTY D104N, *E. coli* WT RmlA, and *P. aeruginosa* (Pseudo) RmlA, and 25 nM of *M. tuberculosis* (Mtb) RmlA, 0.008 mg/mL of PPiase (inorganic pyrophosphatase), 100 μM each of dTTP and Glc-1P (n = 3, error bars represent SD).

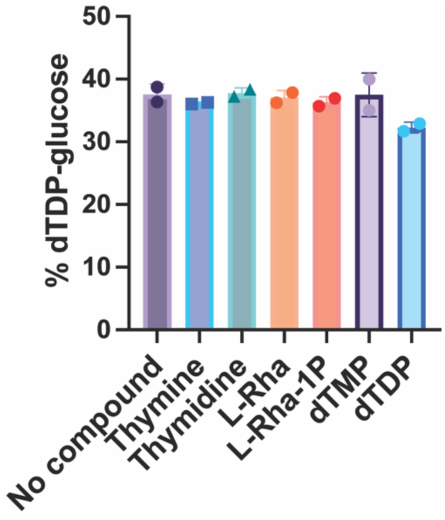

Figure S7. Fragments of dTDP-β-L-Rha (1) do not inhibit RmlA at micromolar concentrations. SALTY RmlA (12.5 nM) was incubated with Glc-1P (100 μM), dTTP (100 μM), and 0.008 mg/mL of PPiase in the absence or presence of indicated fragments (100 μM) of dTDP-β-L-Rha, and percent dTDP-α-Glc produced was measured (t = 6 min, 25 °C). Bars indicate SD (n = 2).

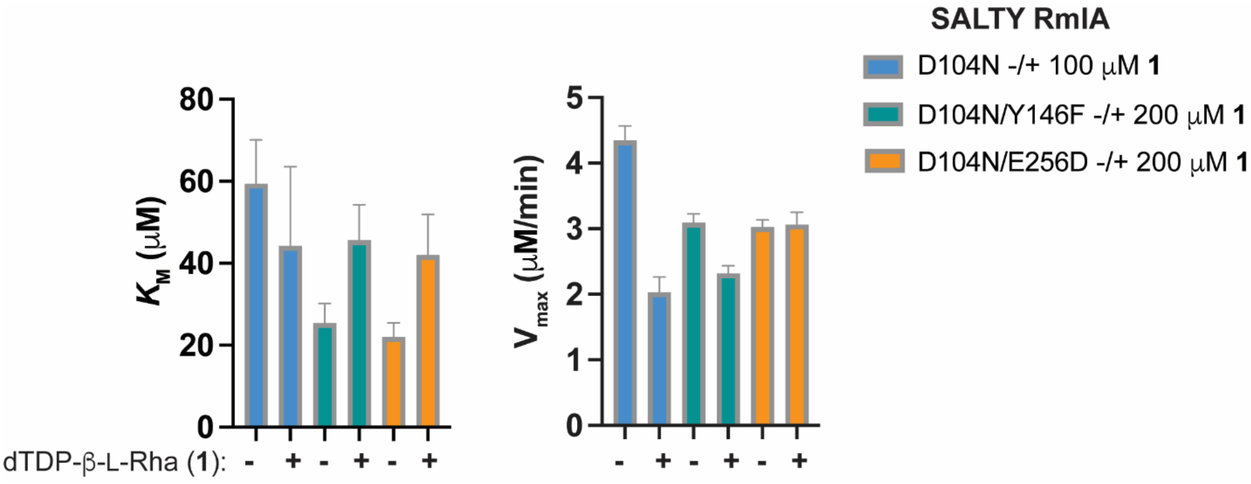

Figure S8. Mutation of E256 in SALTY RmlA leads to a competitive inhibition mechanism by dTDP-β-L-Rha (1). Indicated reactions were set-up in the presence and absence of dTDP-β-L-Rha at concentrations that led to measurable inhibition for each SALTY RmlA construct. While D104N (stabilizing mutation) and D104N/Y146F (active site mutation) experience mixed inhibition by dTDP-β-L-Rha, D104N/E256D (allosteric site mutation) is competitively inhibited by dTDP-β-L-Rha, demonstrating that allosteric regulation is lost upon mutation of E256. Briefly, Glc-1P was fixed at 100 μM and dTTP was titrated. For each reaction, 12.5 nM of RmlA enzyme and 0.008 mg/mL PPiase was pre-incubated at 25 °C in buffer (35 mM Tris-HCl pH 7.5, 2.2 mM $MgCl_2$) +/- dTDP-β-L-Rha prior to addition of substrates. Bars indicate standard error of the mean (SEM, n = 3).

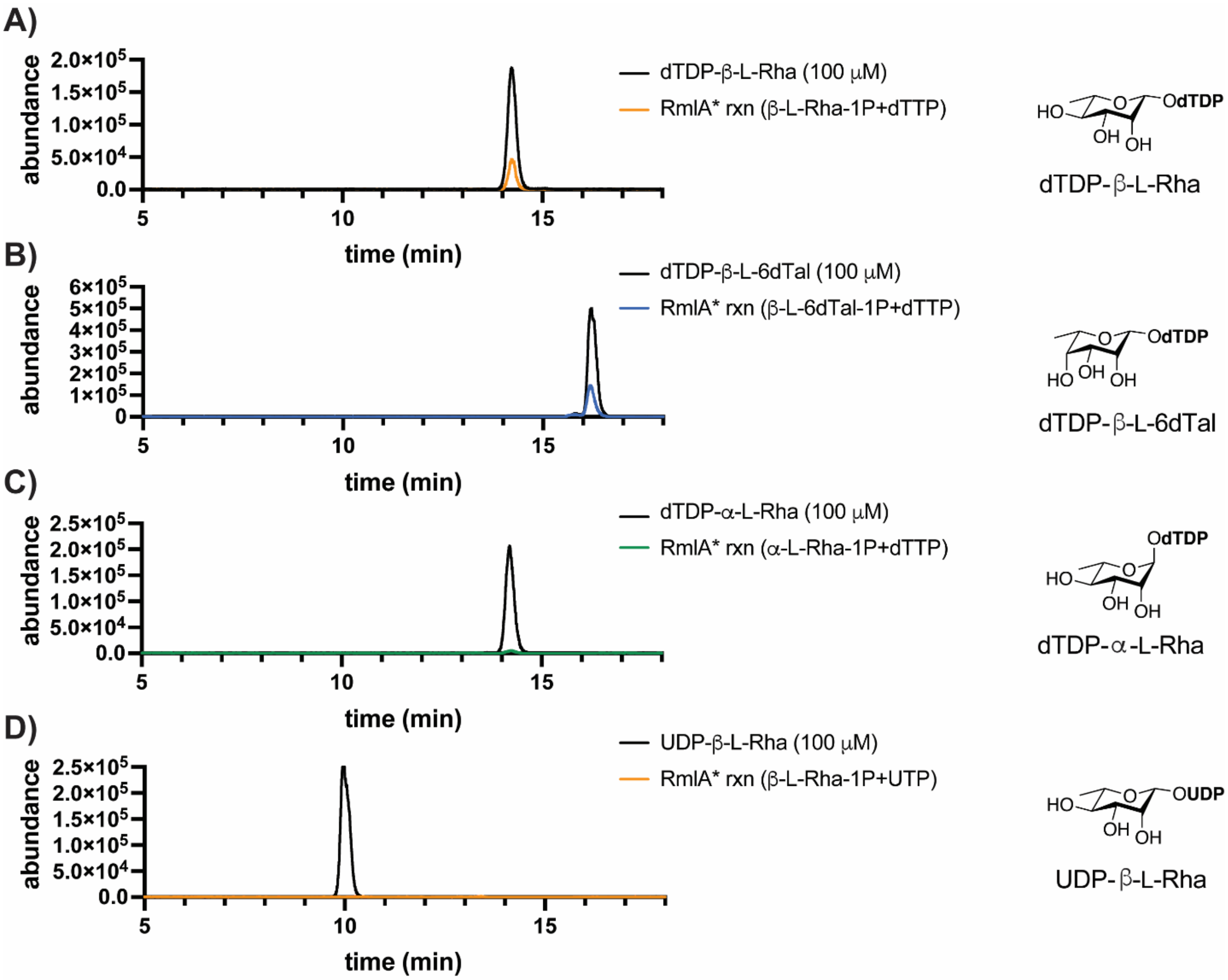

Figure S9. Extracted ion chromatogram (EIC) traces for RmlA* reactions compared to synthetic NDP-sugars indicate enzymatic NDP-L-sugar formation. The indicated synthetic NDP-sugars were analyzed using the same EIC protocol as shown in **Figure 5D** and overlaid with relevant RmlA* (D104N/Y146F/E256D) reactions from **Figure 5D**: (A) dTDP-β-L-Rha, (B) dTDP-β-L-6dTal, (C) dTDP-α-L-Rha, (D) UDP-β-L-Rha. Standards were diluted to a final concentration of 100 μM in reaction buffer (100 mM MOPS, pH 7.5, and 7.5 mM $MgCl_2$). 15 μL of each standard mixture was injected onto a Thermo Scientific LCMS-TQ Fortis system for analysis. $[M-H]^-$ ions corresponding to indicated NDP-sugar products (calculated *m/z*) were extracted: A: 547; B: 547; C: 547; D: 549.

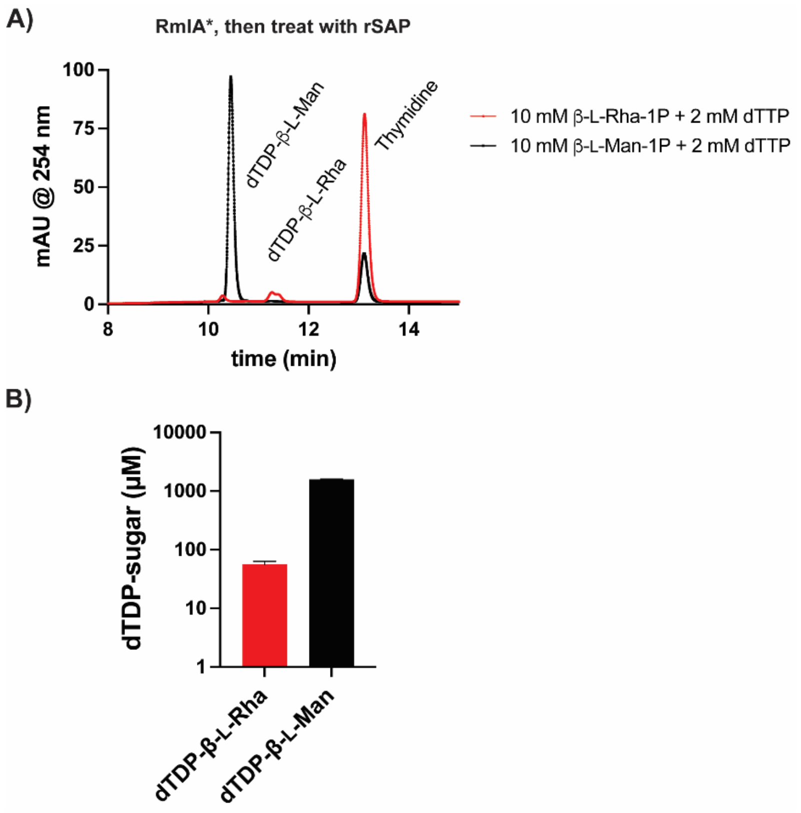

Figure S10. β-L-Man-1P is a better substrate for RmlA* than β-L-Rha-1P. (A) Overlaid HPLC analyses of RmlA* (D104N/Y146F/E256D) reactions containing β-L-Rha-1P or β-L-Man-1-P with dTTP (t = 24 hr, 37 °C) followed by treatment with shrimp alkaline phosphatase (rSAP, t = 2 hr, 37 °C). rSAP degrades remaining dTTP, but not dTDP-sugar, to form thymidine to aid in quantification of product. (B) Quantification of indicated dTDP-sugars from part A.

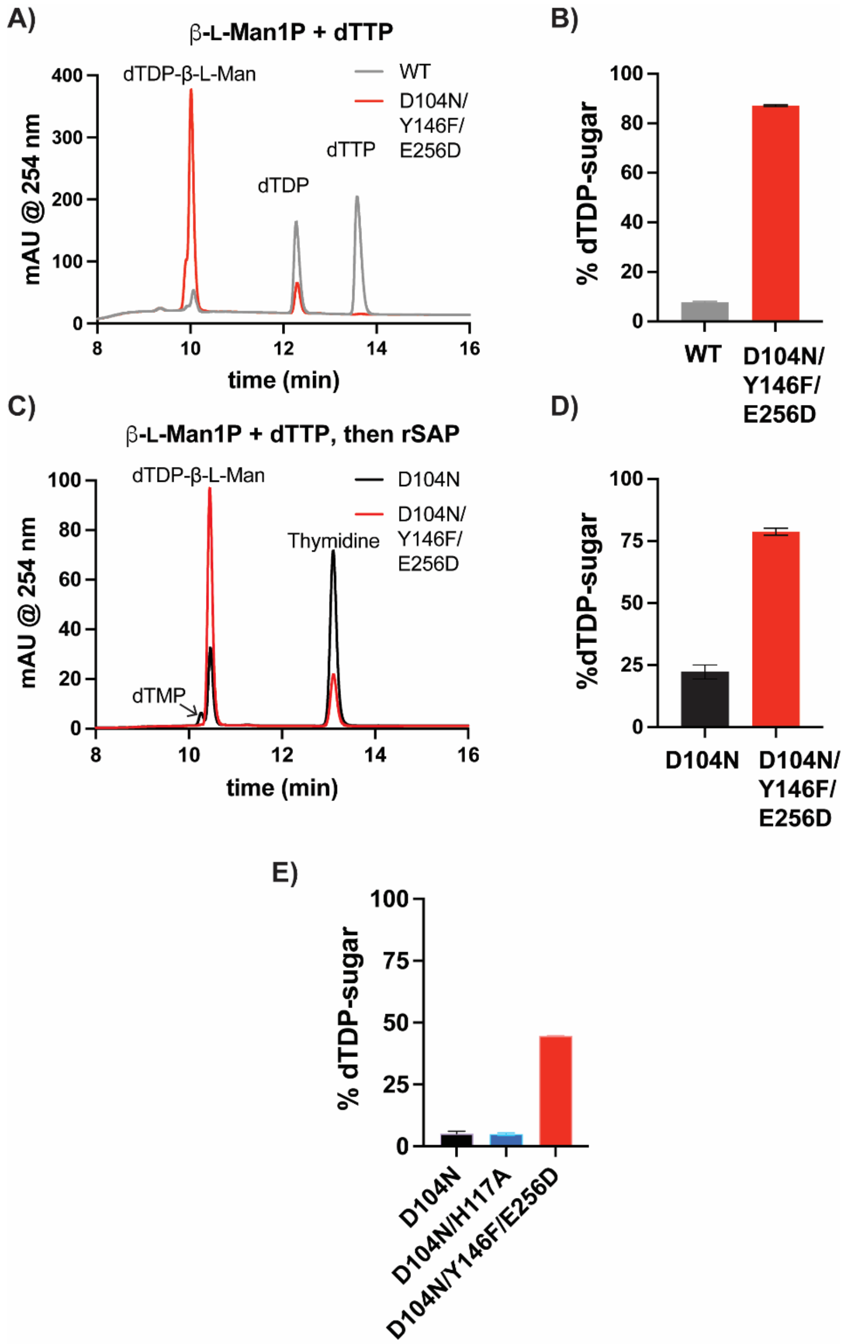

Figure S11. Engineered RmlA* (D104N/Y146F/E256D) can activate a β-L-S-1P substrate in higher yields than the wild-type (WT) or other mutants. (A) Overlaid HPLC analyses of reactions containing β-L-Man-1P and dTTP shows that RmlA* (D104N/Y146F/E256D) can activate the non-canonical substrate, while wild-type RmlA (WT) produces only a small amount of NDP-sugar product. (B) Quantification of dTDP-sugar produced from part A. (C) Overlaid HPLC analyses of indicated reactions demonstrates that activation of the non-canonical substrate, β-L-Man-1P using dTTP nears completion using RmlA* but not RmlA D104N. Note that reaction products were treated with shrimp alkaline phosphatase (rSAP) to confirm production of product and aid in quantification. (D) Quantification of dTDP-sugar produced from part C. (E) Quantification of dTDP-β-L-Man produced with indicated RmlA mutants, including an alternative allosteric site mutation (H117A), indicates RmlA* still shows the highest product production

(performed over a shorter reaction time than parts A and C). Reactions were carried out in a final volume of 30 μL containing 5 μM of indicated RmlA protein, 0.008 mg/mL PPiase, 2 mM dTTP, and 10 mM S-1P (except 2 mM S-1P used in part E) in buffer (100 mM MOPS, pH 7.5, and 7.5 mM $MgCl_2$, 37 °C, t = 24 hr for all but part E, for which t = 6 hr). For part C, 1 μL of rSAP (33.33 U/mL) was subsequently added to each reaction (after t = 24 hr) and incubated for t = 2 hr at 37 °C.

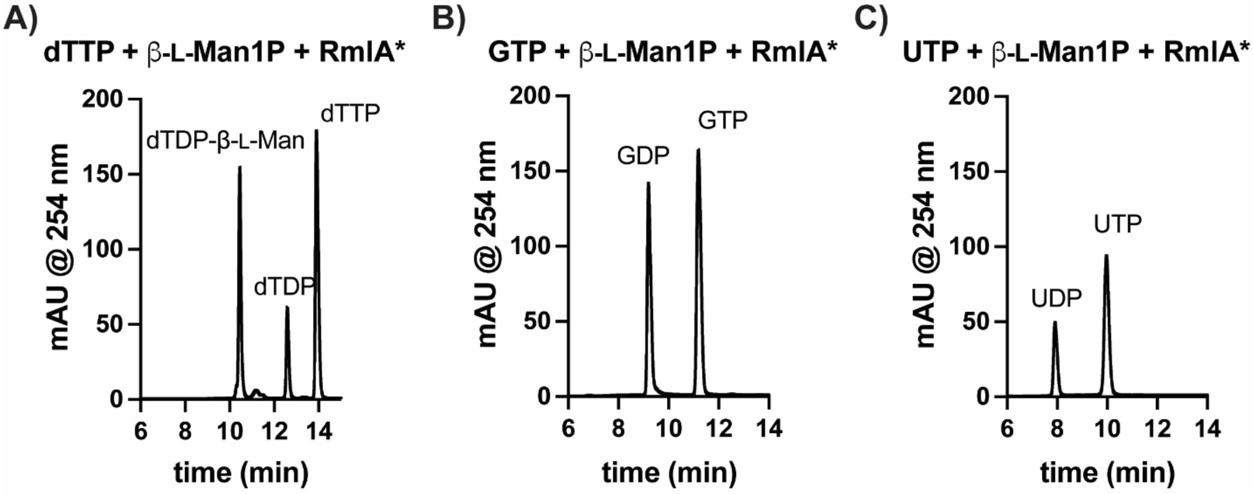

Figure S12. Engineered RmlA* does not activate β-L-Man-1P with non-canonical nucleotides. RmlA* (D104N/Y146F/E256D, 5 μM) was incubated with 2 mM β-L-Man-1P and 2 mM of one of the following: (A) dTTP, (B) GTP, or (C) UTP (t = 6 hr, 37 °C) prior to HPLC analysis. Only the canonical nucleotide, dTTP, could be utilized under these conditions.

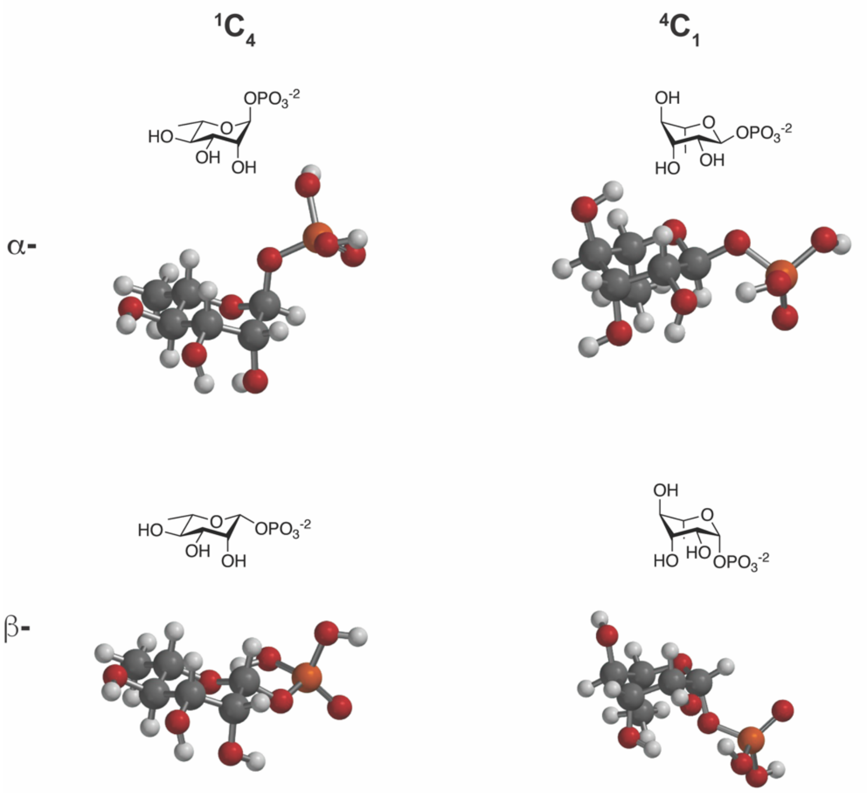

Figure S13. Models of two major chair conformers of the α- and β-anomers of L-Rha-1P to address differences in stability. The $^4C_1$ conformation of β-L-Rha-1P positions the anomeric phosphate similar to that seen in the $^4C_1$ conformation of α-Glc-1P (see **Figure 1B**). See **Table S1** for a comparison of predicted energies of each conformation.

Table S1. Calculation of free energy (kcal/mol) of α-/β-L-**Rha-1P chair conformations.***

	β-anomer	α-anomer	ΔG_b
	ΔG_a	ΔG_a	
L-Rha-1P	-6.44	-0.73	-1.85

Calculation was performed on a cluster computer using Spartan'20 Software. An initial conformational search with Molecular Mechanics (MMFF, ≤ 160 kJ/mol) of a maximal 500 potential conformers produced the lowest energy $^1C_4$ and $^4C_1$ chair conformations, which were then minimized (B3LYP/6-31G) giving the ground-state free energies.[4] The ΔG_a is the free energy gap between $^1C_4$ conformer and $^4C_1$ conformer of each anomer ($E[^1C_4]-E[^4C_1]$), which showed that the $^1C_4$ conformer is thermodynamically more stable among the calculated molecules regardless of the configuration of the anomeric position. ΔG_b is the free energy gap between the $^1C_4$ conformers of the β-anomer and α-anomer ($E\beta[^1C_4]-E\alpha[^1C_4]$). The β-anomer of the free S-1P is predicted to be more stable than the α-anomer.

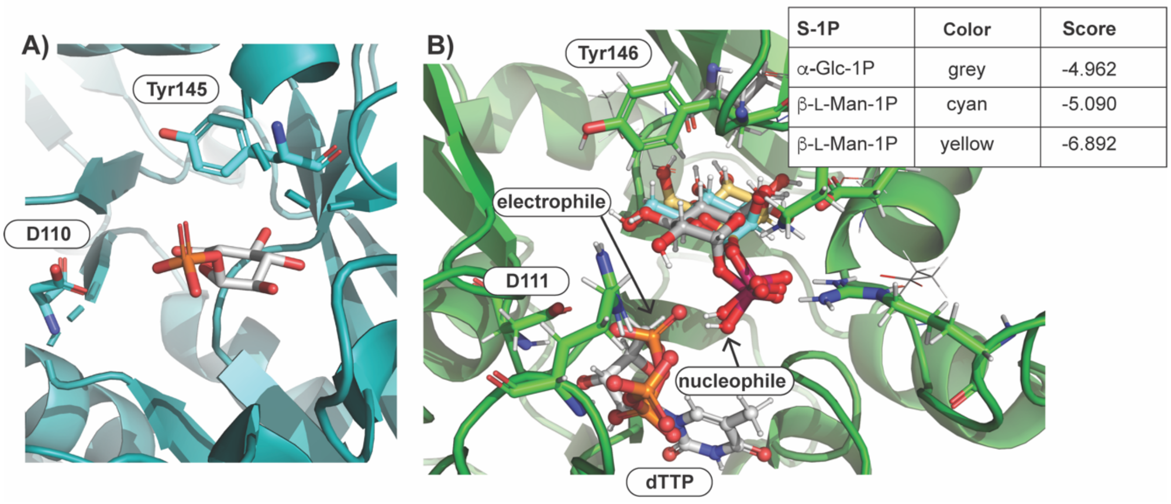

Figure S14. RmlA can accommodate different S-1Ps. (A) Crystal structure of *P. aeruginosa* (Pa) RmlA bound to the native substrate, α-Glc-1P, with relevant residues indicated. The homologous residue to Pa Tyr145 (SALTY Tyr146) has been mutated in SALTY RmlA to expand substrate selectivity and Asp110 is predicted to be involved in catalysis (PDB ID: 1G23). (B) α-Glc-1P docked into SALTY RmlA co-complex with dTTP (PDB ID: 1IIM) using Schrodinger Glide with docking scores shown. β-L-Man-1P (in the lower energy $^{1}C_4$ conformation) is shown in two low energy poses that are similar to the docked α-Glc-1P, which suggests another conformation in which the substrates may bind prior to reaction. The nucleophilic O and electrophilic P of the enzymatic reaction are indicated, along with homologous SALTY residues to those highlighted in Pa RmlA in part A. Molecular docking was performed using Glide (Schrödinger Release 2021–2)[5] loaded with a crystal structure of SALTY RmlA complexed with dTTP. Default parameters were used for optimization, and an OPLS3e force field was employed. The Glide docking grid of the receptor was generated to include the dTTP and predicted S-1P binding pockets. Default van der Waals radii parameters were used. 3D structure of β-L-Man-1P was generated following LigandPrep Wizard from the structure of RmlA-bound α-Glc-1P in a published crystal structure (PDB ID: 1G23). The indicated scores are GlideScores, which approximate the free binding energies of each ligand.

Table S2. Plasmids used in this study.

No.	Plasmid Name	Relevant Features	Notes	References
1	pET28b	pET28b cloning vector	Novagen #69865	N/A
2	pET-His-SUMO	pET His6 SUMO TEV LIC cloning vector (2S-T)	A gift from Scott Gradia (Addgene #29711)	N/A
3	pHYRS52	His6-*S. cerevisiae* Ulp1 (res. 403-621)	Addgene #31122	Muona et al., 2008 [6]
4	pHK255	pET28b-C-term-His-Codon-Optimized-*M. tuberculosis*-RmlA	Protein overexpression vector for Mtb RmlA WT. Made by overlap extension PCR[7] using pET28b and primers pET28b_*M. tuberculosis*_RmlA_Fwd and pET28b_*M. tuberculosis*_RmlA_Rev after amplification of *M. tuberculosis rmlA* gene fragment. DNA was codon optimized for *E. coli* expression as a template (synthetic DNA, Twist Bioscience).	This work
5	pMCZ320	pET28b-C-term-His-SALTY-RmlA	Protein overexpression vector for SALTY RmlA WT	Zheng et al., 2022[8]
6	pMCZ328	pET28b-C-term-His-SALTY-RmlA (D104N)	Protein overexpression vector for SALTY RmlA D104N	Zheng et al., 2022[8]
7	pMCZ348	pET28b-C-term-His-SALTY-RmlA (D104N/Y146F/E256D)	Protein overexpression vector for SALTY RmlA (D104N/Y146F/ E256D) or RmlA*	Zheng et al., 2022[8]
8	pMCZ331	pET28b-C-term-His-SALTY-RmlA (D104N/Y146F)	Protein overexpression vector for SALTY RmlA (D104N/Y146F)	Zheng et al., 2022[8]
9	pMCZ347	pET28b-C-term-His-SALTY-RmlA (D104N/E256D)	Protein overexpression vector for SALTY RmlA (D104N/E256D)	Zheng et al., 2022[8]
10	pMCZ362	pET28b-C-term-His-SALTY-RmlA (D104N/H117A)	Protein overexpression vector for SALTY RmlA (D104N/H117A)	Zheng et al., 2022[8]
11	pMCZ363	pET28b-C-term-His-*P. aeruginosa*-RmlA	Protein overexpression vector for Pa RmlA	Zheng et al., 2022[8]
12	pMCZ382	pET His6 SUMO-*E. coli*-RmlA	Protein overexpression vector for *E. coli* RmlA	Zheng et al., 2022[8]

Table S3. Primers used in this study.

Oligo Name	5' - 3' Sequence
pET28b_*M. tuberculosis*_RmlA_Fwd	TTTGTTTAACTTTAAGAAGGAGATATACC ATGCGCGGTATCATTCTGGCCGGCGGTAGTGG
pET28b_*M. tuberculosis*_RmlA_Rev	GCCGGATCTCAGTGGTGGTGGTGGTGGTG GTTGCGCTCCAGCAGTTCGAGCAGATAATTAC

Table S4. Strains used in this study.

No.	Strain Name	Strain information	Source
1	*E. coli* MG1655	Wildtype *E. coli* strain	Gift from Rojas Lab (NYU)
2	BL21 (DE3)	*E. coli* cells engineered for high level protein expression	Novagen
3	Rosetta2(DE3) pLysS	*E. coli* Rosetta2 expressing T7 lysozyme	Novagen
4	EcHK255	BL21 transformed with plasmid 4 (**Table S2**) for C-term His *M. tuberculosis*-RmlA overexpression	This work

5	*E. coli* expressing C-term His SALTY-RmlA wild-type and mutants	BL21 transformed with plasmids 5-10 for C-term His SALTY-RmlA wild-type and mutant overexpression	Zheng et al., 2022[8]
6	EcMCZ363	BL21 transformed with plasmid 11 for C-term His *P. aeruginosa*-RmlA wild-type overexpression	Zheng et al., 2022[8]
7	EcMCZ382	Rosetta2 pLysS transformed with plasmid 12 for His-SUMO-*E. coli*-RmlA overexpression	Zheng et al., 2022[8]

Supporting Experimental Methods and Data

General Information. All chemicals were purchased from Sigma Aldrich, Alfa Aesar, or Fisher Scientific without further purification. ^{1}H NMR, ^{13}C NMR, ^{31}P NMR spectra were recorded on Bruker Avance III 400 MHz, Bruker Avance III 600 MHz, or Bruker AV4 800 MHz, calibrated using residual non-deuterated solvent as an internal reference, processed by MestReNova (authorized to NYU). HRMS analyses were acquired on an Agilent 6224 Accurate-Mass time-of-flight LC/MS (LC-TOF) spectrometer with an electrospray (ESI) ionization source equipped with an autosampler. Extracted Ion Chromatograms were obtained on a Thermo Scientific LCMS-TQ Fortis. Preparative HPLC purification was performed with a Thermo Fisher Ultimate 3000 HPLC System through a 5 µm, 21.2 × 150 mm, NX-C18, 110 Å Phenomenex Gemini preparative column using acetonitrile/50 mM TEAB buffer gradient as the mobile phase (5 mL/min). Analytical HPLC analysis was performed with Thermo Fisher Ultimate 3000 HPLC System equipped with autosampler through a 5 µm, 4.6 × 150 mm, NX-C18, 110 Å Phenomenex Gemini analytical column using acetonitrile/50 mM TEAB buffer gradient as the mobile phase (1 mL/min). Primers were purchased from Invitrogen and sequencing was done by Genewiz. FPLC purification and analysis was performed using an AKTA pure 15 L instrument (UNICORN™ software, GE Healthcare). Molecular graphics and analyses of protein structures were performed with PyMOL 2.5. GraphPad Prism software 9.0 was used for data analysis and plotting. pET His6 Sumo TEV LIC cloning vector (2S-T) was a gift from Scott Gradia (Addgene plasmid # 29711; http:// n2t.net/addgene:29711; RRID:Addgene_29711).

1. General experimental methods for synthetic compounds

1.1 Ion exchange for isolation of triethylammonium (TEA) salt of nucleotides and S-1Ps

Commercially purchased nucleoside-monophosphates (dTMP, UMP and GMP) and Glc-1P were dissolved in water (0.1-0.2 M). The solution was shaken with 2.5 equivalents of IR-120 H^{+} type ion exchange resin at room temperature for 1 hour. The solution was filtered, and the resin was washed with three times the resin volume with water. The combined filtrate was cooled in an ice-bath and two equivalents of TEA was slowly added into the shaking flask. The neutralized solution was lyophilized, and the number of counter-ions was determined by ^{1}H NMR.

1.2 Chemical activation of (d)NMPs

NMP triethylammonium salt was dissolved in dry DMF (4 mL/mmol), and 2.5 equivalents of carbonyldiimidazole (CDI) was added. The reaction was stirred at room temperature until no NMP was observed on ESI-MS. Dry methanol (0.1 mL/mmol (d)NMP) was added in an ice-bath, and the mixture was stirred for another 30 minutes to quench the excess CDI. The reaction solution was poured into 100 mM $NaClO_4$ in acetone-ether (v/v = 1:1). The resulting suspension was stirred for another 10 minutes and centrifuged (4 °C, 1857 x g, 5 min). The precipitation was collected, washed with cold ether, and centrifuged again. The crude (d)NMP-imidazolide (dNMP-Im) was obtained as a white/pale yellow solid after drying under a vacuum. The successful activation of NMP was confirmed by ^{31}P NMR analysis, with product formation indicated by a ~-8.6 ppm signal and no monophosphate signal.

2 Experimental Synthetic Procedures and Data

2.1 **Synthesis of S-1P analogs**

Synthesis of β-L-Rhamnose-1-phosphate (3)

β-L-Rha-1-phosphate (3) was synthesized following a published protocol reported by Sebasen *et. al.* [9] with slight modification.

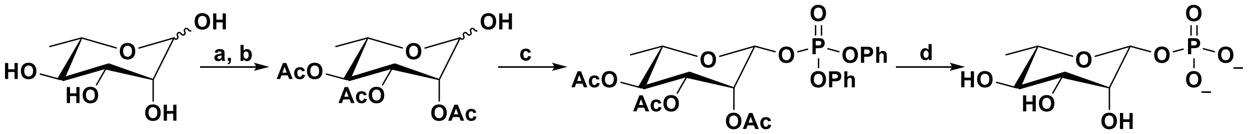

L-Rha 6a 9a 3

Scheme S1. Synthesis of *β*-**L-Rha-1-P** (3) a) Ac_2O, DMAP, Py, 0 °C to r.t.; b) $BnNH_2$, THF, r.t.; c) $(PhO)_2POCl$, DMAP, DCM, r.t.; d) (1) H_2, PtO_2, EtOH-EtOAc, r.t., (2) TEA-MeOH-H_2O, 55 °C.

Synthesis of compound **6a**

3 g of L-rhamnose was stirred with 893 mg (0.4 eq) of DMAP in 25 mL of pyridine (Py) at 0 °C. 17.3 mL (10 eq) of acetic anhydride (Ac_2O) was added in portions over 30 minutes. The reaction was warmed to room temperature and kept stirring for 18 hours. The reaction mixture was then poured into 100 mL of ice-cooled water, and extracted with 150 mL of ethyl acetate (EA). The organic layer was washed with 50 mL of 1M HCl, 50 mL of water, 50 mL of sat. $NaHCO_3$ (aq.), and 50 mL of brine, then dried over Na_2SO_4. The crude product was obtained after filtration and evaporation, and directly used in the next step without further purification.

The crude product of acetylation was stirred with 3 mL (1.5 eq) of benzylamine ($BnNH_2$) in 25 mL of dry tetrahydrofuran (THF) at room temperature overnight. The solvent was removed. The residue was dissolved with EA, washed with water and sat. $NaHCO_3$ (aq.), and dried over Na_2SO_4. The product was obtained after silica gel flash chromatography (Hex/EA = 5:1 to 3:1 to 1:1) with a yield of 62% over two steps and a weight of 3.27 g of pale-yellow oil as the final product.

^{1}H NMR (400 MHz, $CDCl_3$) δ 5.37 (dd, *J* = 10.1, 3.4 Hz, 1H, H-3), 5.27 (dd, *J* = 3.4, 1.8 Hz, 1H, H-2), 5.16 (d, *J* = 1.9 Hz, 1H, H-1), 5.08 (t, *J* = 9.9 Hz, 1H, H-4), 4.17 – 4.09 (m, 1H, H-5), 2.15 (s, 3H), 2.05 (s, 3H), 1.99 (s, 3H), 1.22 (d, *J* = 6.3 Hz, 3H) ppm

^{13}C NMR (101 MHz, $CDCl_3$) δ 170.37, 170.21 (CH_3**CO**), 92.35 (C-1), 71.25 (C-5), 70.29 (C-3), 68.90 (C-4), 66.60 (C-2), 21.08, 20.97, 20.89 (**C**H_3CO), 17.63 (C-6).

ESI-TOF-MS (m/z) $[M+Na]^+$ calculated for $C_{12}H_{18}O_3Na$ 313.0894; found: 313.0916

Synthesis of compound **9a**

1.45 g of the compound **6a** and 1.22 g (2 eq) of DMAP were stirred in 15 mL of dry DCM at room temperature. 2.1 mL (2 eq) of diphenyl phosphoryl chloride in 6 mL of dry DCM was added to the reaction mixture dropwise over a period of 1 hour. The reaction solution was stirred for another 2 hours. The reaction was diluted with 50 mL of DCM, washed with 40 mL of $NaHCO_3$ (sat. aq.), 40 mL of 0.1 M HCl, and then dried over Na_2SO_4, filtered, and concentrated. The crude product was purified via silica gel flash chromatography (Hex:EA = 5:1 to 7:2 to 3:1) to give 1.61 g of pale yellow solid as a major product **9a** (β-phosphate, 62%) and 0.71 g of pale yellow oil as a minor product **9a-α** (α-anomer, 27%).

Compound **9a**

^{1}H NMR (400 MHz, $CDCl_3$) δ 7.34 (ddd, J = 10.3, 5.3, 2.7 Hz, 4H, Ph), 7.27 – 7.22 (m, 2H, Ph), 7.20 – 7.15 (m, 4H, Ph), 5.54 (dd, J = 7.0, 1.3 Hz, 1H, H-1), 5.47 (dt, J = 3.2, 1.0 Hz, 1H, H-2), 5.09 – 4.98 (m, 2H, H-4, H-3), 3.61 (dq, J = 9.2, 6.2 Hz, 1H, H-5), 2.11 (s, 3H, COC**H**$_3$), 2.05 (s, 3H, COC**H**$_3$), 1.98 (s, 3H, COC**H**$_3$), 1.24 (d, J = 6.2 Hz, 3H, H-6) ppm.

^{13}C NMR (101 MHz, $CDCl_3$) δ 170.06, 169.98, 169.95 (**C**OCH_3), 130.01, 129.79 (*meta*-C, Ph), 125.95, 125.73 (*para*-C, Ph), 120.60 (d, *J* = 5.1 Hz), 120.42 (d, *J* = 5.1 Hz), (*ortho*-C, Ph), 94.91 (d, *J* = 4.0 Hz, C-1), 71.58 (C-5), 70.53 (C-3), 70.03 (C-4), 68.65, (d, *J* = 9.1 Hz, C-2), 20.91, 20.80, 20.68 (CO**C**H_3), 17.37 (C-6) ppm.

^{31}P NMR (162 MHz, CDCl3) δ -13.74 ppm.

ESI-TOF-MS (m/z)[M+Na]$^+$ calculated for $C_{24}H_{27}O_{11}PNa$ 545.1183; found: 545.1180.

Compound **9a-α**

^{1}H NMR (400 MHz, $CDCl_3$) δ 7.34 (ddd, *J* = 8.7, 7.1, 4.7 Hz, 4H, Ph), 7.28 – 7.22 (m, 4H, Ph), 7.22 – 7.16 (m, 2H, Ph), 5.81 (dd, *J* = 6.7, 1.6 Hz, 1H, H-1), 5.29 (d, *J* = 9.3 Hz, 2H, H-2, H-4), 5.09 (t, *J* = 9.6 Hz, 1H, H-3), 3.92 (dq, *J* = 10.0, 6.2 Hz, 1H, H-5), 2.10 (s, 3H, COC**H**$_3$), 2.01 (s, 3H, COC**H**$_3$), 1.97 (s, 3H, COC**H**$_3$), 1.09 (d, *J* = 6.3 Hz, 3H, H-6) ppm.

^{13}C NMR (101 MHz, $CDCl_3$) δ 169.52, 169.47, 169.27 (**C**OCH_3), 150.07 (d, *J* = 7.0 Hz, -**C**-O, Ph), 149.87 (d, *J* = 7.3 Hz, -**C**-O, Ph)), 129.79, 129.70 (*meta*-C, Ph), 125.61, 125.45 (*para*-C, Ph), 120.01 (d, *J* = 4.7 Hz), 119.78 (d, *J* = 5.1 Hz) (*ortho*-C, Ph), 96.05 (d, *J* = 5.6 Hz, C-1), 69.74 (C-5), 68.71, 68.60 (C-3 & C-4), 67.97 (C-2), 20.42, 20.36, 20.31 ($COCH_3$), 16.88 (C-6) ppm.

^{31}P NMR (162 MHz, $CDCl_3$) δ -14.19 ppm.

ESI-TOF-MS (m/z) [M+Na]$^+$ calculated for $C_{24}H_{27}O_{11}PNa$ 545.1183; found: 545.1170

Synthesis of β-L-rhamnose-1-phosphate (**3**)

104 mg of compound **9a** was stirred with 28 mg of PtO_2 (0.6 eq) in 2 mL of EA/2 mL of ethanol under 1.1 atm of H_2 overnight. The catalyst was filtered out and the solvent was concentrated to dryness. The crude product was dissolved in a mixture of 1 mL of TEA/2 mL of water/2 mL of methanol and stirred at 55 °C over 36 hours. The solvent was removed, and residue was dissolved with 10 mL of water, washed 3 times with 3 mL of dicholoromethane (DCM). The aqueous layer was lyophilized, giving 78 mg of pale-yellow oil as product **3** (L-Rha-1P ·nTEA, n = 2 determined by ^{1}H NMR) with a yield of 87%.

^{1}H NMR (400 MHz, D_2O) δ 5.11 (dd, *J* = 8.6, 1.0 Hz, 1H, H-1), 4.03 (d, *J* = 3.3 Hz, 1H, H-2), 3.65 (dd, *J* = 9.3, 3.3 Hz, 1H, H-3), 3.50 – 3.33 (m, 2H, H-4 & H-5), 3.21 (q, *J* = 7.3 Hz, 12H, CH_2 of TEA), 1.32 (d, *J* = 5.9 Hz, 3H, H-6), 1.28 (t, *J* = 7.3 Hz, 18H, , CH_3 of TEA).

^{13}C NMR (101 MHz, D_2O) δ 95.03 (d, *J* = 3.0 Hz, C-1), 72.56 (C-5), 72.29 (C-4), 71.70 (C-3), 71.13 (d, *J* = 5.5 Hz, C-2), 46.64 (**C**H_2 of TEA), 16.71 (C-6), 8.19 (**C**H_3 of TEA).

^{31}P NMR (162 MHz, D_2O) δ -1.38 ppm.

ESI-TOF-MS (m/z) [M-H]$^-$ calculated for $C_6H_{12}O_8P$ 243.0275; found: 243.0282.

Synthesis of α-L-rhamnose-1-phosphate (**3-α**)

103 mg of compound **9a-α** was stirred with 28 mg of PtO_2 (0.6 eq) in 2 mL of ethyl acetate/2 mL of ethanol under 1.1 atm of H_2 overnight. The catalyst was filtered out and the solvent was concentrated to dryness. The crude product was dissolved in a mixture of 1 mL of TEA/2 mL of water/2 mL of methanol and stirred at 55 °C over 36 hours. The solvent was removed, and the residue was dissolved in 10 mL of water, and washed 3 times with 3 mL of DCM. The aqueous layer was lyophilized, giving 75 mg of pale-yellow oil as product **3-α** (L-Rha-1P ·nTEA, n = 2 determined by ^1H NMR) with a yield of 84%.

^1H NMR (400 MHz, D_2O) δ 5.31 (dd, J = 7.8, 2.0 Hz, 1H, H-1), 3.97 (dd, J = 3.4, 2.1 Hz, 1H, H-2), 3.89 (ddt, J = 9.8, 6.4, 3.4 Hz, 2H, H-3 and H-5), 3.43 (t, J = 9.8 Hz, 1H, H-4), 3.20 (q, J = 7.3 Hz, 12H, CH_2 of TEA), 1.28 (t, J = 7.3 Hz, 21H, CH_3 of TEA and H-6).

^{13}C NMR (101 MHz, D_2O) δ 95.42 (d, J = 5.2 Hz, C-1), 72.15 (C-5), 70.77 (d, J = 8.2 Hz, C-2), 69.69, 69.13 (C-3 and C-4), 46.63 (**C**H_2 of TEA), 16.75 (d, J = 3.3 Hz, C-6), 8.19 (**C**H_3 of TEA).

^{31}P NMR (162 MHz, D_2O) δ – 0.98 ppm.

ESI-TOF-MS (m/z) $[M-H]^-$ calculated for $C_6H_{12}O_8P$ 243.0275; found: 243.0286.

Synthesis of L-Mannose-1-phosphate (**4**)

Synthesis of β-L-Man-1P (**4**) was synthesized following a protocol similar to that of **3**.

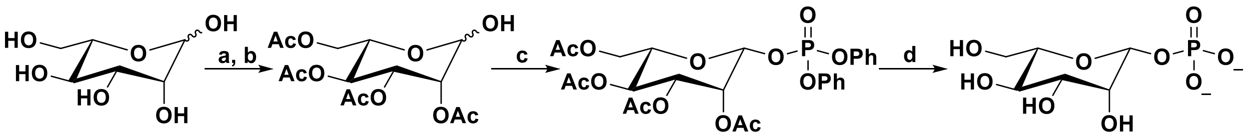

Scheme S2. Synthesis of *β*-L-Man-1P (**4**): a) Ac_2O, DMAP, Py, 0 °C to r.t.; b) $BnNH_2$, THF, r.t.; c) $(PhO)_2POCl$, DMAP, DCM, r.t.; d) (1) H_2, PtO_2, EtOH-EtOAc, r.t., (2) TEA-MeOH-H_2O, 55 °C.

Synthesis of compound **6b**

1 g of L-mannose was stirred with 366 mg (0.5 eq) of DMAP in 10 mL of Py at 0 °C. 7 mL (12.5 eq) of Ac_2O was added in portions over 30 minutes. The reaction was warmed to room temperature and kept stirring for 18 hours. The reaction mixture was poured into 40 mL of ice-cooled water, and then extracted with 80 mL of EA. The organic layer was washed with 30 mL of 1M HCl, 30 mL of water, 30 mL of sat. $NaHCO_3$ (aq.), and 30 mL of brine, then dried over Na_2SO_4. The crude product was obtained after filtration and evaporation, and directly used in the next step without further purification.

The crude product of acetylation was stirred with 1 mL (1.5 eq) of $BnNH_2$ in 10 mL of dry THF at room temperature overnight. The solvent was removed. The residue was dissolved in EA, washed with water and sat. $NaHCO_3$ (aq.), then dried over Na_2SO_4. The product was obtained after silica gel flash chromatography (Hex/EA = 5:1 to 3:1 to 1:1) as 1.62 g of pale-yellow oil as product with a yield of 81%.

^1H NMR (400 MHz, $CDCl_3$) δ 5.42 (dd, J = 10.1, 3.4 Hz, 1H, H-1), 5.34 – 5.24 (m, 3H, H-2~H4), 4.29 – 4.21 (m, 2H, H-5, H-6a), 4.14 (dt, J = 11.4, 2.1 Hz, 1H, H-6b), 3.20 (d, J = 4.1 Hz, <1H, -OH), 2.16 (s, 3H, C**H**$_3$CO-), 2.11 (s, 3H, C**H**$_3$CO-), 2.05 (s, 3H, C**H**$_3$CO-), 2.00 (s, 3H, C**H**$_3$CO-) ppm.

^{13}C NMR (101 MHz, $CDCl_3$) δ 170.93, 170.30, 170.13, 169.94 (CH_3**C**O-), 92.40 (H-1), 70.04 (C-5), 68.83 (C-3), 68.74 (C-2), 66.29 (C-4), 62.70 (H-6), 21.05, 20.93, 20.87, 20.84 (**C**H_3CO-) ppm.

ESI-TOF-MS (m/z) $[M+Na]^+$ calculated for $C_{14}H_{20}O_{10}Na$ 371.0949; found: 371.0986.

Synthesis of compound **9b**

1.60 g of **6b** and 1.12 g (2 eq) of DMAP were stirred in 20 mL of dry DCM at room temperature. 1.9 mL (2 eq) of diphenyl phosphoryl chloride in 6 mL of dry DCM was added into the reaction mixture dropwise over a period of 1 hour. The reaction solution was stirred for another 2 hours. The reaction was diluted with 50 mL of DCM, washed with 40 mL of $NaHCO_3$ (sat. aq.), 40 mL of 0.1 M HCl, and then dried over Na_2SO_4, filtered, and concentrated. The product was purified via silica gel flash chromatography (Hex:EA = 3:1 to 3:2 to 2:3) and to give 1.72 g of pale yellow oil as the major product **9b** (β-phosphate, 64%) and 0.60 g of pale yellow oil as the minor product **9b-α** (α-anomer, 22%).

Compound **9b**

^{1}H NMR (400 MHz, $CDCl_3$) δ 7.31 (td, *J* = 8.1, 7.7, 2.5 Hz, 4H, Ph), 7.25 (d, *J* = 8.1 Hz, 2H, Ph), 7.20 – 7.14 (m, 4H, Ph), 5.63 (dd, *J* = 7.2, 1.3 Hz, 1H, H-1), 5.48 (d, *J* = 3.7 Hz, 1H, H-2), 5.24 (t, *J* = 9.6 Hz, 1H, H-4), 5.09 (dd, *J* = 9.7, 3.4 Hz, 1H, H-3), 4.25 (dd, *J* = 12.3, 5.5 Hz, 1H, H-6a), 4.09 (dd, *J* = 12.2, 2.7 Hz, 1H, H-6b), 3.78 (ddd, *J* = 9.1, 5.5, 2.7 Hz, 1H, H-5), 2.07 (s, 3H, COC**H**$_3$), 2.02 (s, 6H, 2x COC**H**$_3$), 1.96 (s, 3H, COC**H**$_3$) ppm.

^{13}C NMR (101 MHz, $CDCl_3$) δ 170.45, 169.76, 169.65, 169.51 (-**C**OCH$_3$), 150.32 (d, *J* = 7.7 Hz), 149.95 (d, *J* = 7.3 Hz)(-**C**-O, Ph), 129.84, 129.63 (*meta*-C, Ph), 125.79 (d, *J* = 1.4 Hz), 125.62 (d, *J* = 1.5 Hz) (*para*-C, Ph), 120.39 (d, *J* = 4.7 Hz), 120.21 (d, *J* = 4.7 Hz) (*ortho*-C, Ph), 94.73 (d, *J* = 4.7 Hz, C-1), 72.99 (C-5), 70.07 (C-3), 68.07 (d, *J* = 8.8 Hz, C-2), 65.33 (C-4), 61.97 (C-6), 20.62, 20.60, 20.54, 20.43 (-CO**C**H$_3$) ppm.

^{31}P NMR (162 MHz, $CDCl_3$) δ -13.8 ppm.

ESI-TOF-MS (m/z) $[M+Na]^+$ calculated for $C_{26}H_{29}O_{13}PNa$ 603.1238; found: 603.1256.

Compound **9b-α**

^{1}H NMR (400 MHz, $CDCl_3$) δ 7.38 (ddd, *J* = 9.1, 7.1, 2.1 Hz, 4H, H-Ar), 7.30 – 7.20 (m, 6H, H-Ar), 5.88 (dd, *J* = 6.7, 1.9 Hz, 1H, H-1), 5.41 – 5.31 (m, 3H, H-2, H-3, H-4), 4.20 (dd, *J* = 12.5, 4.8 Hz, 1H, H-6a), 4.09 (ddd, *J* = 9.4, 4.6, 2.2 Hz, 1H, H-5), 3.94 (dd, *J* = 12.4, 2.3 Hz, 1H, H-6b), 2.17 (s, 3H, COC**H**$_3$), 2.06 (s, 3H, COC**H**$_3$), 2.02 (s, 3H, COC**H**$_3$), 1.99 (s, 3H, COC**H**$_3$) ppm.

^{13}C NMR (101 MHz, $CDCl_3$) δ 170.63, 169.84, 169.61 (-**C**OCH$_3$), 150.24 (d, *J* = 7.0 Hz), 150.04 (d, *J* = 7.2 Hz)(-**C**-O, Ph), 130.12, 130.06 (*meta*-C, Ph), 125.90 (d, *J* = 1.5 Hz), 125.78 (d, *J* = 1.4 Hz) (*para*-C, Ph), 120.22 (d, *J* = 4.7 Hz), 120.07 (d, *J* = 5.0 Hz) (*ortho*-C, Ph), 96.06 (d, *J* = 5.7 Hz, C-1), 70.80 (C-5), 68.63 (d, *J* = 11.7 Hz, C-2), 68.25 (C-3), 65.18 (C-4), 61.74 (C-6), 20.79, 20.73, 20.68, 20.66 ppm.

^{31}P NMR (162 MHz, $CDCl_3$) δ -14.2 ppm.

ESI-TOF-MS (m/z) $[M+Na]^+$ calculated for $C_{26}H_{29}O_{13}PNa$ 603.1238; found: 603.1119.

Synthesis of β-L-mannose-1-phosphate (**4**)

180 mg of compound **9b** was stirred with 43 mg of PtO_2 (0.6 eq) in 2 mL of EA/2 mL of ethanol under 1.1 atm of H_2 overnight. The catalyst was filtered out and the solvent was concentrated to dryness. The crude product was dissolved in a mixture of 1 mL of TEA/2 mL of water/2 mL of methanol and stirred at 55 °C

over 36 hours. The solvent was removed, and the resulting residue was dissolved with 10 mL of water, and then washed 3 times with 3 mL of DCM. The aqueous layer was lyophilized, giving 163 mg of pale-yellow oil as product **4** (L-Man-1P ·nTEA, n = 3 determined by ^{1}H NMR) with a yield of 94%.

^{1}H NMR (400 MHz, D_2O) δ 5.16 (dd, *J* = 8.4, 1.1 Hz, 1H, H-1), 4.02 (d, *J* = 3.3 Hz, 1H, H-2), 3.93 (dd, *J* = 12.2, 2.2 Hz, 1H, H-6a), 3.77 – 3.65 (m, 2H, H-6b, H-3), 3.55 (t, *J* = 9.7 Hz, 1H, H-4), 3.44 (ddd, *J* = 9.5, 6.8, 2.2 Hz, 1H, H-5), 3.20 (q, *J* = 7.4 Hz, 17H, CH_2 of TEA), 1.28 (t, *J* = 7.3 Hz, 24H, CH_3 of TEA) ppm.

^{13}C NMR (101 MHz, D_2O) δ 95.05 (d, *J* = 4.3 Hz, C-1), 76.70 (C-5), 72.56 (C-3), 71.06 (d, *J* = 6.5 Hz, C-2), 66.59 (C-4), 61.17 (C-6), 46.63 (**C**H_2 of TEA), 8.19 (**C**H_2 of TEA).

^{31}P NMR (162 MHz, D_2O) δ -1.24 ppm.

ESI-TOF-MS (m/z) [M-H]$^-$ calculated for $C_6H_{12}O_9P$ 259.0224; found: 259.0233.

Synthesis of α-L-mannose-1-phosphate (**4-α**)

180 mg of compound **9b-α** was stirred with 43 mg of PtO_2 (0.6 eq) in 2 mL of EA/2 mL of ethanol under 1.1 atm of H_2 overnight. The catalyst was filtered out and the solvent was concentrated to dryness. The crude product was dissolved in a mixture of 1 mL of TEA/2 mL of water/2 mL of methanol and stirred at 55 °C over 36 hours. The solvent was removed, and the resulting residue was dissolved with 10 mL of water, and then washed 3 times with 3 mL of DCM. The aqueous layer was lyophilized, giving 133 mg of pale-yellow oil as product **4-α** (L-Man-1-P ·nTEA, n = 2.2 determined by ^{1}H NMR) with a yield of 89%.

^{1}H NMR (400 MHz, D_2O) δ 5.37 (dd, *J* = 8.2, 2.0 Hz, 1H, H-1), 3.96 (dd, *J* = 3.4, 2.0 Hz, 1H, H-2), 3.94 – 3.90 (m, 1H, H-5), 3.90 – 3.86 (m, 1H, H-6a), 3.86 – 3.82 (m, 1H, H-3), 3.77 – 3.71 (m, 1H, H-6b), 3.63 (t, *J* = 9.8 Hz, 1H, H-4), 3.19 (q, *J* = 7.3 Hz, 13H), 1.27 (t, *J* = 7.3 Hz, 20H) ppm.

^{13}C NMR (101 MHz, D_2O) δ 95.36 (d, *J* = 4.9 Hz, C-1), 73.18 (C-3), 70.73 (d, *J* = 8.0 Hz, C-2), 69.94 (C-4), 66.73 (C-5), 60.99 (C-6), 46.61 (**C**H_2 of TEA), 8.18 (**C**H_3 of TEA) ppm.

^{31}P NMR (162 MHz, D_2O) δ -0.75 ppm.

ESI-TOF-MS (m/z) [M-H]$^-$ calculated for $C_6H_{12}O_9P$ 259.0224; found: 259.0223.

Synthesis of 6-deoxy-β-L-talose-1-phosphate (5)

This is the first reported synthesis of β-L-6dTal-1P (**5**). The synthesis of precursor **8** was performed following a protocol reported by Danieli *et. al.* [10] with slight modifications.

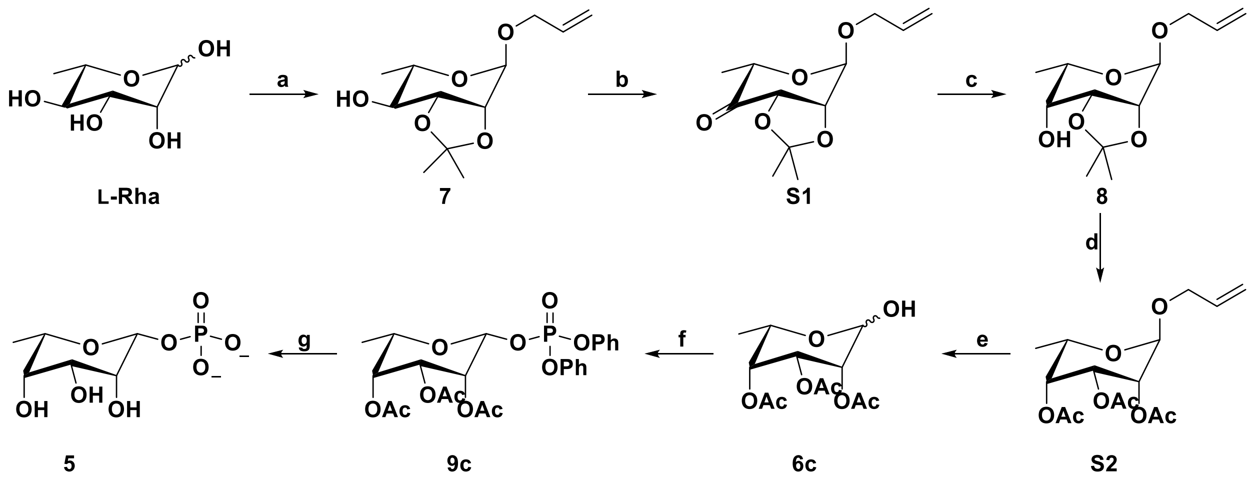

Scheme S3. Synthesis of *β*-L-6dTal-1P (**5**): a) (1) IR-120, H^+ exchange resin, allyl alcohol, reflux, (2) $Me_2C(OMe)_2$, acetone, BF_3-Et_2O; b) oxalyl chloride, DMSO, DCM, -78 °C; c) $NaBH_4$, EtOH, 0 °C; d) (1) 10 % AcOH, 50 °C, (2) Ac_2O, Py, r.t.; e) $PdCl_2$, DCM-MeOH; f) $(PhO)_2POCl$, DMAP, DCM, r.t.; g) (1) H_2, PtO_2, EtOH-EtOAc, r.t., (2) TEA-MeOH-H_2O, 55 °C.

Synthesis of compound **7**

4 g of L-rhamnose was refluxed in a stirring solution of 30 mL of allylic alcohol in the presence of 1.2 g of IR-120 H^+ resin for 2 hours, and monitored by TLC (DCM:Acetone = 1:1). The mixture was filtered and concentrated to be used for the next step without further purification.

To a solution of the allyl rhamnoside in 1:1 (v/v) acetone/2,2-dimethoxypropane (30 mL/30 mL), 0.1 mL of $BF_3·Et_2O$ (0.04 eq) was added and the resulting solution was stirred at room temperature until the reaction was complete as indicated by TLC analysis (Hex:EA = 2:1). The reaction was quenched with the addition of 0.2 mL of TEA and filtered. The filtrate was concentrated and purified via silica gel flash chromatography (Hex:EA = 4:1) to give 3.6 g of pale yellow oil as the product with a yield of 61%.

^1H NMR (400 MHz, d_6-Acetone) δ 6.00 – 5.88 (m, 1H, -C**H**=), 5.29 (dq, *J* = 17.3, 1.8 Hz, 1H, =C**H**$_2$a), 5.16 (dq, *J* = 10.5, 1.5 Hz, 1H, =C**H**$_2$b), 4.94 (s, 1H, H-1), 4.45 (d, *J* = 5.5 Hz, 1H, 4-OH), 4.17 (ddt, *J* = 13.1, 5.2, 1.6 Hz, 1H, -OC**H**$_2$a-), 4.09 (d, *J* = 5.7 Hz, 1H, H-2), 4.04 – 3.93 (m, 2H, -OC**H**$_2$b-, H-3), 3.55 (dq, *J* = 10.0, 6.4 Hz, 1H, H-5), 3.30 – 3.20 (m, 1H, H-4), 1.42 (s, 3H, -CC**H3**), 1.29 (s, 3H, -CC**H3**), 1.21 (d, *J* = 6.3 Hz, 3H, H-6) ppm.

^{13}C NMR (101 MHz, d_6-Acetone) δ 135.41, 117.06, 109.35, 97.13, 79.68, 76.86, 75.08, 68.24, 66.74, 28.38, 26.53, 17.74 ppm.

ESI-TOF-MS (m/z) $[M+Na]^+$ calculated for $C_{12}H_{20}O_5Na$ 267.1203; found: 267.1171.

Synthesis of compound **S1**

3.3 mL (4 eq) of dry DMSO was added dropwise into a stirring solution of 2 mL (2 eq) of oxalyl chloride in 15 mL of dry DCM at -78 °C. A solution of 2.87 g of compound **7** in 30 mL of dry DCM was added into the stirring solution over a 1 hour period at -78 °C. After the addition of 8.2 mL (4 eq) of DIPEA, the reaction mixture was warmed to room temperature and stirred for another hour until TLC analysis indicated that the reaction was complete. The mixture was quenched by the addition of an equal volume of sat.

$NaHCO_3$ and extracted with 50 mL of DCM. The organic layer was dried over Na_2SO_4 and concentrated to dryness, giving the crude product that could be used directly in the next step.

Synthesis of compound **8**

Crude **S1** was stirred in 40 mL of ethanol at 0 °C. 450 mg (1 eq) of $NaBH_4$ was added and the reaction was stirred at 0 °C for another hour. The reaction was quenched by the addition of 20 mL of brine. The resulting mixture was extracted with EA (3 x 50 mL). The combined organic layers were dried over Na_2SO_4, concentrated to dryness, and purified via silica gel flash chromatography (Hex:EA = 100:0 to 9:1 to 4:1), giving 2.26 g of colorless oil as product **8** with a yield of 79%.

^{1}H NMR (400 MHz, d_6-Acetone) δ 5.99 – 5.87 (m, 1H, -C**H**=), 5.29 (dq, J = 17.3, 1.8 Hz, 1H, =C**H**$_2$a), 5.15 (dq, J = 10.5, 1.6 Hz, 1H, =C**H**$_2$b), 4.96 (d, J = 1.5 Hz, 1H, H-1), 4.26 – 4.16 (m, 2H, -OC**H**$_2$a-, H-3), 4.06 – 3.99 (m, 2H, -OC**H**$_2$b-, H-2), 3.86 (qd, J = 6.6, 1.8 Hz, 1H, H-5), 3.58 (ddd, J = 6.7, 4.7, 1.8 Hz, 1H, H-4), 2.83 (d, J = 7.0 Hz, 1H, 4-OH), 1.49 (s, 3H, -CC**H3**), 1.32 (s, 3H, -CC**H3**), 1.24 (d, J = 6.6 Hz, 3H, H-6) ppm.

^{13}C NMR (101 MHz, d_6-Acetone) δ 135.53 (-**C**H=), 116.93 (=**C**H$_2$), 109.68 (-**C**Me$_2$), 97.74 (**C**-1), 74.63 (C-2), 74.41 (C-3), 68.59 (-O**C**H$_2$-), 67.42 (C-4), 66.02 (C-5), 26.25, 25.65 (-C(CH$_3$)$_2$), 17.06 (C-6) ppm.

ESI-TOF-MS (m/z) $[M+Na]^+$ calculated for $C_{12}H_{20}O_5Na$ 267.1203; found: 267.1210.

Synthesis of compound **S2**

2.26 g of **8** in 10% acetic acid (AcOH,100 ml) was stirred at 50 °C for 5 hour, and monitored by TLC (1:1 Hex/EA). The mixture was co-evaporated with toluene, then the residue was dissolved in 30 mL of dry Py. 678 mg (0.6 eq) of DMAP and 5.3 mL (6 eq) of Ac_2O was added, and the solution was stirred overnight at room temperature. The reaction mixture was poured into 60 mL of ice-cooled water, and extracted with 2 x 100 mL of EA. The organic layer was washed with 50 mL of 1 M HCl, 50 mL of water, 50 mL of sat. $NaHCO_3$ (aq), and 50 mL of brine, then dried over Na_2SO_4. The crude product after filtration and evaporation was purified via silica gel flash chromatography (Hex:EA = 100:0 to 8:1 to 7:2) to give 2.71 g of pale-yellow oil as product with a yield of 89%.

^{1}H NMR (400 MHz, d_6-Acetone) δ 5.95 (dddd, J = 17.3, 10.4, 5.9, 5.2 Hz, 1H, -C**H**=), 5.31 (dq, J = 17.2, 1.7 Hz, 1H, =C**H**$_2$a), 5.23 (t, J = 3.8 Hz, 1H, H-3), 5.18 (dq, J = 10.5, 1.5 Hz, 1H, =C**H**$_2$b), 5.13 (ddt, J = 3.5, 1.6, 0.7 Hz, 1H, H-4), 5.03 (dt, J = 3.9, 1.3 Hz, 1H, H-2), 4.87 (d, J = 1.6 Hz, 1H, H-1), 4.21 (dddd, J = 10.4, 5.2, 4.0, 2.4 Hz, 2H, , H-5, -OC**H**$_2$a-), 4.05 (ddt, J = 12.9, 5.8, 1.4 Hz, 1H, -OC**H**$_2$b-), 2.11 (s, 3H, C**H**$_3$CO-), 2.08 (s, 3H, C**H**$_3$CO-), 1.91 (s, 3H, C**H**$_3$CO-), 1.15 (d, J = 6.5 Hz, 3H, H-6).

^{13}C NMR (101 MHz, Acetone-d$_6$) δ 171.02, 170.55, 169.88 (CH$_3$**C**O-), 134.96 (-**C**H=), 117.52(=**C**H$_2$), 98.30 (C-1), 69.54, 68.74 (-O**C**H$_2$-), 67.77, 67.00, 65.66, 20.80, 20.65, 20.58, 16.39 (C-6)) ppm.

ESI-TOF-MS (m/z) $[M+Na]^+$ calculated for $C_{15}H_{22}O_8Na$ 353.1207; found: 353.1219.

Synthesis of compound **6c**

2.12 g of compound **S2** was dissolved in 15 mL of DCM /15 mL of methanol, stirred with 454 mg (0.4 eq) of $PdCl_2$ at room temperature for 4 hours. The mixture was filtered through a layer of Celite and washed with DCM. The filtrate was diluted with 70 mL of DCM and extracted with 0.1 N HCl. The organic layer

was dried over Na_2SO_4, filtrated, and then concentrated to dryness. The crude product was purified via silica gel flash chromatography (Hex:EA = 2:1 to 1:1) giving 1.15 g of colorless oil as product with a yield 63%.

^{1}H NMR (400 MHz, $CDCl_3$) δ 5.36 (t, *J* = 3.7 Hz, 1H, H-3), 5.29 (t, *J* = 2.2 Hz, 1H, H-1), 5.19 (dt, *J* = 3.2, 1.3 Hz, 1H, H-4), 5.13 (dt, *J* = 4.0, 1.2 Hz, 1H, H-2), 4.42 (qd, *J* = 6.6, 1.6 Hz, 1H, H-5), 2.75 (d, *J* = 3.8 Hz, 1H, 1-OH), 2.16 (s, 3H, C**H**$_3$CO-), 2.15 (s, 3H, C**H**$_3$CO-), 2.00 (s, 3H, C**H**$_3$CO-), 1.22 (d, *J* = 6.6 Hz, 3H, H-6) ppm.

^{13}C NMR (101 MHz, $CDCl_3$) δ 170.73, 170.31, 169.85 (CH_3**C**O-), 93.33 (C-1), 69.15 (C-4), 67.62 (C-2), 65.85 (C-3), 65.02 (C-5), 21.15, 20.89, 20.82 (**C**H$_3$CO-), 16.36 (C-6) ppm.

ESI-TOF-MS (m/z) $[M+Na]^+$ calculated for $C_{12}H_{18}O_3Na$ 313.0894; found: 313.0851

Synthesis of compound **9c**

457 mg of compound **6c** and 391 mg (2 eq) of DMAP were stirred in 10 mL of dry DCM at room temperature. 663 μL (2 eq) of diphenyl phosphoryl chloride in 5 mL of dry DCM was added into the reaction mixture dropwise over a period of 1 hour. The reaction solution was stirred for another 2 hours. The reaction was diluted with 20 mL of DCM, washed by 20 mL of sat. $NaHCO_3$ (aq.) and 20 mL of 0.1 M HCl, dried over Na_2SO_4, filtered, and concentrated. The crude product was purified via silica gel flash chromatography (Hex:EA = 3:1 to 3:2) to give 383 mg of pale yellow oil as the major product **9c** (β-phosphate, 52%); however, the corresponding α-anomer minor product was not obtained.

^{1}H NMR (400 MHz, $CDCl_3$) δ 7.31 (td, *J* = 7.9, 4.0 Hz, 4H, Ar-H), 7.26 – 7.24 (m, 2 H, Ar-H), 7.19 – 7.14 (m, 4H, Ar-H), 5.51 (dd, *J* = 7.5, 1.6 Hz, 1H, H-1), 5.37 – 5.36 (m, 1H, H-2), 5.09 (dt, *J* = 2.4, 1.2 Hz, 1H, H-4), 5.05 (t, *J* = 3.7 Hz, 1H, H-3), 3.84 (qd, *J* = 6.5, 1.6 Hz, 1H, H-5), 2.13 (s, 3H, C**H**$_3$CO-), 2.07 (s, 3H, C**H**$_3$CO-), 1.96 (s, 3H, C**H**$_3$CO-), 1.22 (d, *J* = 6.8 Hz, 3H, H-6).

^{13}C NMR (101 MHz, $CDCl_3$) δ 170.52, 170.00, 169.52 (CH_3**C**O-), 150.52 (d, *J* = 7.3 Hz), 150.14 (d, *J* = 7.5 Hz))(-**C**-O, Ph) , 129.94, 129.76 (*metha*-C of Ph), 125.87 (d, *J* = 1.4 Hz), 125.64 (d, *J* = 1.4 Hz) (*para*-C of Ph), 120.49 (d, *J* = 4.8 Hz), 120.40 (d, *J* = 4.7 Hz), (*ortho*-C, Ph), 95.34 (d, *J* = 4.4 Hz, C-1), 71.13 (C-5), 67.85 (d, *J* = 1.5 Hz, C-3), 67.39 (C-4), 66.45 (d, *J* = 8.5 Hz, C-2), 20.78, 20.73, 20.57 (**C**H$_3$CO-), 15.98 (C-6) ppm.

^{31}P NMR (162 MHz, $CDCl_3$) -13.49 ppm

ESI-TOF-MS (m/z) $[M+Na]^+$ calculated for $C_{24}H_{27}O_{11}PNa$ 545.1183; found: 545.1199

Synthesis of compound **5**

188 mg of compound **9c** was stirred with 52 mg of PtO_2 in 2 mL of EA/2 mL of ethanol under 1.1 atm of H_2 overnight. The catalyst was filtered out and the solvent was concentrated to dryness. The crude product was dissolved in a mixture of 1 mL TEA/2 mL of water/2 mL of methanol and stirred at 55 °C over 36 hours. The solvent was removed, and the resulting residue was dissolved with 10 mL of water, and then washed 3 times with 3 mL of DCM. The aqueous layer was lyophilized, giving 108 mg of pale-yellow oil as product **5** (β-L-6dTal-1P·nTEA, n = 1.5 determined by ^{1}H NMR) with a yield of 76%.

^{1}H NMR (400 MHz, D_2O) δ 5.06 (dt, *J* = 8.7, 0.9 Hz, 1H, H-1), 3.99 – 3.95 (m, 1H, H-2), 3.83 (t, *J* = 3.3 Hz, 1H, H-3), 3.78 – 3.71 (m, 1H, H-5), 3.68 – 3.65 (m, 1H, H-4), 3.21 (q, *J* = 7.3 Hz, 9H, CH_2 of TEA), 1.35 – 1.24 (m, 17H, CH_3 of TEA and H-6) ppm.

^{13}C NMR (101 MHz, D_2O) δ 95.48 (d, *J* = 3.8 Hz, C-1), 72.10 (C-5), 71.14 (C-4), 70.95 (d, *J* = 5.8 Hz, C-2), 68.30 (C-3), 46.64 (**C**H_2 of TEA), 15.42 (C-6), 8.19 (**C**H_3 of TEA).

^{31}P NMR (162 MHz, D_2O) δ -1.20 ppm.

ESI-TOF-MS (m/z) $[M+HCOO]^-$ calculated for $C_7H_{14}O_{10}P$ 289.0330; found: 289.0338

2.2 Screening of phosphate coupling conditions and synthesis of NDP-sugars

2.2.A Catalyst screening for the coupling of Glc-1P with dTMP-Im (**Figure S1**)

0.05 mmol of Glc-1P·nTEA (n = 1.7 TEA) and 0.065 mmol (1.3 equivalents) of preactivated dTMP-Im was dissolved in 1.6 mL of anhydrous DMF. 0.2 mmol (4 equivalents) of each indicated catalyst was added to identical reaction mixtures. The reaction was stirred at room temperature for over 120 hours. 0.14 mL of the reaction mixture was taken at certain time points, quenched with 0.56 mL of 0.1 M ammonium bicarbonate aqueous solution (NH_4HCO_3), and mixed with 0.1 mL of D_2O for NMR analysis (^{31}P NMR).

2.2.B Short time course of coupling reactions with Glc-1P (**Figure S2**) or β-L-Fuc-1P (**Figure S3**) or β-L-Rha-1P (**Figure S4**)

0.05 mmol of Glc-1P·nTEA (n = 1.7 TEA) or L-Fuc-1P·nTEA (n = 2 TEA) or β-L-Rha-1P·nTEA (n = 2 TEA), and 0.065 mmol (1.3 equivalents) of preactivated dTMP-Im were dissolved in 1.6 mL of anhydrous DMF, with or without the addition of 0.2 mmol (4 equivalents) of *N*-methyl imidazolium chloride (NMI-HCl) to the reaction mixture. The reaction was stirred at room temperature for over 18 hours. 0.1 mL of reaction mixture was taken at indicated time points, quenched with 0.5 mL of 0.1 M NH_4HCO_3, and mixed with 0.1 mL of D_2O for NMR analysis (^{31}P NMR).

2.2.C Chemical synthesis of NDP-sugars via coupling of S-1Ps and activated NMPs

1 equivalent of S-1P was stirred with 1.3 equivalent of activated NMP-Im dissolved in dry DMF. 4 equivalents of N-methylimidazolium chloride (NMI-HCl) was added and the mixture was stirred for 3 hours. The reaction was quenched with the addition of 10 mL 0.1 M NH_4HCO_3 at 0 °C. After 10 minutes of stirring, the solvent was removed by lyophilization. The crude product was purified by semi-preparative HPLC (Thermo Fisher Ultimate 3000) using a Gemini column (Phenomenex, 5 μm, 21.2 × 150 mm, NX-C18, 110 Å) with acetonitrile (ACN) and 50 mM TEAB buffer as the mobile phase (0-5 min, 2% ACN; 5-35 min, 2-6 % ACN; 35-40 min, 6-100% ACN; 40-45 min, 100% ACN; 45-50 min, 100-2% ACN; 50-55 min, 2% ACN) at a flow rate of 5 mL/min). The yield of each NDP-sugar was calculated by the absorbance at 260 nm of the relevant NDP as measured by Nano Drop (Thermo Scientific).

Table S5. Summarized final yields of NDP-sugars obtained via coupling reactions post-purification.

S-1-P (mg)	NMP-Im (mg)	NMI-HCl (mg)	Product (μmol/separation yield)
**3** (54 mg)	dTMP-Im (66 mg)	52	dTDP-β-L-Rha (**1**, 26 μmol/24%)
**3-α** (55 mg)	dTMP-Im (69 mg)	61	dTDP-α-L-Rha (**10**, 18 μmol/14%)
**4** (28 mg)	dTMP-Im (37 mg)	24	dTDP-β-L-Man (**11**, 5.6 μmol/11.2%)
**5** (45 mg)	dTMP-Im (57 mg)	42	dTDP-β-L-6dTal (**2**, 14.4 μmol/16%)
**3** (27 mg)	UMP-Im (34 mg)	26	UDP-β-L-Rha (**12**, 4.8 μmol/8.9%)*
**3** (27 mg)	GMP-Im (39 mg)	27	GDP-β-L-Rha (**13**, 2.7 μmol/5%)*

* Low yield due to the formation of cyclic carbonate during the activation of UMP and GMP.

dTDP-β-L-rhamnose (1)

^{1}H NMR (400 MHz, D_2O) δ 7.69 (d, *J* = 1.4 Hz, 1H, H-6 Thymine), 6.38 (t, *J* = 7.0 Hz, 1H, H-1' dRib), 5.22 (dd, *J* = 8.8, 1.0 Hz, 1H, H-1'' Rha), 4.62 (dt, *J* = 4.8, 2.6 Hz, 1H, H-3' dRib), 4.17 (d, *J* = 4.4 Hz, 3H, H-4' dRib, H-5'a dRib, H-5'b dRib), 4.09 (d, *J* = 3.3 Hz, 1H, H-2'' Rha), 3.64 (dd, *J* = 9.4, 3.3 Hz, 1H, H-3'' Rha), 3.48 – 3.34 (m, 2H, H-4'' Rha, H-5'' Rha), 3.15 (q, *J* = 7.3 Hz, 16H, CH_2 of TEA), 2.41 – 2.29 (m, 2H, H-2' dRib), 1.92 (d, *J* = 1.2 Hz, 3H, 5-Me Thymine), 1.31 (d, *J* = 6.0 Hz, 3H, H-6'' Rha), 1.26 (t, *J* = 7.3 Hz, 26H, CH_3 of TEA) ppm.

^{13}C NMR (101 MHz, D_2O) δ 170.06 (C-4 Thymine), 154.40 (C-2 Thymine), 136.94 (C-6 Thrymine), 111.85 (C-5 Thymine), 95.52 (d, *J* = 4.0 Hz, C-1'' Rha), 85.15 (d, *J* = 9.1 Hz, C-4' dRib), 84.95 (C-1' dRib), 72.69 (C-5'' Rha), 72.13 (C-4'' Rha), 71.64 (C-3'' Rha), 71.02 (C-3' dRib), 70.79 (d, *J* = 5.8 Hz, C-2'' Rha), 65.44 (d, *J* = 5.7 Hz, C-5' dRib), 46.55 (**C**H_2 TEA), 38.62 (C-2' dRib), 16.72 (**C**H_3, C-6'' Rha), 12.04 (d, *J* = 6.6 Hz, **C**H_3 Thymine), 8.29 (**C**H_3 TEA) ppm.

^{31}P NMR (162 MHz, D_2O) δ -11.59 (d, *J* = 20.6 Hz), -13.75 (d, *J* = 20.7 Hz) ppm.

ESI-TOF-MS (m/z) [M-H]$^-$ calculated for $C_{16}H_{25}N_2O_{15}P_2$ 547.0736; found: 547.0742

dTDP-α-L-rhamnose (10)

^{1}H NMR (400 MHz, D_2O) δ 7.74 (d, *J* = 1.4 Hz, 1H, H-6 Thymine), 6.37 (t, *J* = 7.0 Hz, 1H, H-1' dRib), 5.43 (dd, *J* = 7.6, 2.0 Hz, 1H, H-1'' Rha), 4.62 (dt, *J* = 5.8, 3.2 Hz, 1H, H-3' dRib), 4.18 (qd, *J* = 4.1, 1.6 Hz, 3H, H-4' dRib, H-5'a dRib, H-5'b dRib), 4.04 (dd, *J* = 3.4, 2.0 Hz, 1H, H-2'' Rha), 3.97 – 3.84 (m, 2H, H-3'' and H-5'' Rha), 3.44 (t, *J* = 9.8 Hz, 1H, H-4'' Rha), 3.18 (q, *J* = 7.3 Hz, 34H, CH_2 of TEA), 2.43 – 2.32 (m, 2H, H-2' dRib), 1.93 (d, *J* = 1.2 Hz, 3H, 5-Me Thymine), 1.27 (t, *J* = 7.4 Hz, 54H, CH_3 of TEA and H-6'' Rha) ppm.

^{13}C NMR (101 MHz, D_2O) δ 168.79 (C-4 Thymine), 153.37 (C-2 Thymine), 137.13 (C-6 Thymine), 111.82 (C-5 Thymine), 96.30 (d, *J* = 6.0 Hz, C-1'' Rha), 85.28 (d, *J* = 9.2 Hz, C-4' dRib), 84.96 (C-1' dRib), 72.00 (C-5'' Rha), 71.08 (C-3' dRib), 70.38 (d, *J* = 9.4 Hz, C-2'' Rha), 69.64, 64.57 (C-3'' and C-4'' Rha), 65.42 (d, *J* = 5.5 Hz, C-5' dRib), 46.60 (**C**H_2 TEA), 38.60 (C-2' dRib), 16.78 (**C**H_3, C-6'' Rha), 11.90 (**C**H_3 Thymine), 8.26 (**C**H_3 TEA) ppm.

^{31}P NMR (162 MHz, D_2O) δ -11.74 (d, *J* = 20.6 Hz), -14.00 (d, *J* = 21.1 Hz) ppm.

ESI-TOF-MS (m/z) [M-H]$^-$ calculated for $C_{16}H_{25}N_2O_{15}P_2$ 547.0736; found: 547.0779

dTDP-β-L-mannose (11)

^{1}H NMR (400 MHz, D_2O) δ 7.71 (d, *J* = 1.4 Hz, 1H, H-6 Thymine), 6.37 (t, *J* = 7.0 Hz, 1H, H-1' dRib), 5.26 (dd, *J* = 8.4, 1.0 Hz, 1H, H-1'' Man), 4.63 (p, *J* = 2.8 Hz, 1H, 1H, H-3' dRib), 4.18 (d, *J* = 4.4 Hz, 3H, H-4' dRib, H-5'a dRib, H-5'b dRib), 4.09 (d, *J* = 3.5 Hz, 1H, H-2'' Man), 3.92 (dd, *J* = 12.3, 2.2 Hz, 1H, H-6''a Man), 3.76 – 3.68 (m, 2H, H-3'' & H6''b Man), 3.56 (t, *J* = 9.7 Hz, 1H, H-4'' Man), 3.44 (ddd, *J* = 9.5, 6.6, 2.2 Hz, 1H, H-5'' Man), 3.17 (q, *J* = 7.3 Hz, 14H, CH_2 of TEA), 2.41 – 2.32 (m, 2H, H-2' dRib), 1.92 (d, *J* = 4.2 Hz, 3H, 5-Me Thymine), 1.27 (t, *J* = 7.3 Hz, 23H, CH_3 of TEA) ppm.

^{13}C NMR (101 MHz, D_2O) δ 168.59 (C-4 Thymine), 153.26 (C-2 Thymine), 137.10 (C-6 Thymine), 111.82 (C-5 Thymine), 95.54 (d, *J* = 4.5 Hz, C-1'' Man), 85.24 (d, *J* = 9.2 Hz, C-4' dRib), 84.96 (C-1' dRib), 76.82 (C-5'' Man), 72.41 (C-3'' Man), 71.03 (C-3' dRib), 70.70 (d, *J* = 6.7 Hz, C-2'' Man), 66.45 (C-4'' Man), 65.45 (d, *J* = 5.9 Hz, C-5' dRib), 61.08 (C-6'' Man), 46.59 (**C**H_2 TEA), 38.60 (C-2' dRib), 11.84 (**C**H_3 Thymine), 8.25 (**C**H_3 TEA) ppm.

^{31}P NMR (162 MHz, D_2O) δ -11.70 (d, *J* = 21.1 Hz), -13.46 (d, *J* = 21.0 Hz) ppm.

ESI-TOF-MS (m/z) [M-H]$^-$ calculated for $C_{16}H_{25}N_2O_{16}P_2$ 563.0685; found: 563.0708

dTDP-6-deoxy-β-L-talose (2)
^{1}H NMR (400 MHz, D_2O) δ 7.76 (d, *J* = 1.2 Hz, 1H, H-6 Thymine), 6.36 (dd, *J* = 7.6, 6.4 Hz, 1H, H-1' dRib), 5.17 (dd, *J* = 8.8, 1.2 Hz, 1H, H-1" dTal), 4.64 (dt, *J* = 6.0, 3.0 Hz, 1H, H-3' dRib), 4.19 (d, *J* = 4.6 Hz, 3H, H-4' dRib, H-5'a dRib, H-2" dTal), 4.09 – 4.03 (m, 1H, H5' dRib), 3.82 (t, *J* = 3.3 Hz, 1H, H-3" dTal), 3.79 – 3.70 (m, 1H, H-5" dTal), 3.64 (dt, *J* = 3.5, 1.2 Hz, 1H, H-4" dTal), 3.20 (t, *J* = 7.3 Hz, 10H, CH2 of TEA), 2.44 – 2.32 (m, 2H, H-2' dRib), 1.94 (d, *J* = 1.2 Hz, 3H, 5-Me Thymine), 1.32 – 1.26 (m, 20H, CH_3 of TEA and H-6" dTal) ppm.
^{13}C NMR (201 MHz, D_2O) δ 166.56 (C-4 Thymine), 151.73 (C-2 Thymine), 137.36 (C-6 Thymine), 111.77 (C-5 Thymine), 95.93 (d, J = 3.8 Hz, C1" 6dTal), 85.35 (d, J = 8.9 Hz, C-4' dRib), 84.95 (C-1' dRib), 72.22 (C-5" 6dTal), 71.18 (C-4" 6dTal), 70.99 (C-3' dRib), 70.65 (d, J = 5.8 Hz, C-2" 6dTal), 68.23 (C-3" 6dTal), 65.44 (d, J = 5.8 Hz, C-5' dRib), 46.65 (**C**H_2 TEA), 38.58 (C-2' dRib), 15.45 (**C**H_3 C-6" 6dTal), 11.64 (**C**H_3 Thymine), 8.21 (**C**H_3 TEA) ppm.
^{31}P NMR (162 MHz, D_2O) δ -11.61 (d, *J* = 21.3 Hz), -13.76 (d, *J* = 21.2 Hz) ppm.
ESI-TOF-MS (m/z) [M-H]$^-$ calculated for $C_{16}H_{25}N_2O_{15}P_2$ 547.0736; found: 547.0740

UDP-β-L-rhamnose (12)
^{1}H NMR (400 MHz, D_2O) δ 7.87 (d, *J* = 7.8 Hz, 1H, H-6 Ura), 6.02 (d, *J* = 4.7 Hz, 1H, H-5 Ura), 5.92 (d, *J* = 7.8 Hz, 1H, H-1' Rib), 5.23 (dd, *J* = 8.8, 1.1 Hz, 1H, H-1" Rha), 4.36 (p, *J* = 5.3 Hz, 2H, H2' & H3' Rib), 4.28 – 4.17 (m, 3H, H-4', H5'a & H5'b Rib), 4.09 (d, *J* = 3.3 Hz, 1H, H-2" Rha), 3.64 (m, 1H, H-3" Rha), 3.47 – 3.36 (m, 2H, H-4" & H-5" Rha), 3.17 (q, *J* = 7.3 Hz, 58H, CH_2 TEA), 1.31 (d, *J* = 6.0 Hz, 3H, CH_3 H-6" Rha), 1.27 (t, *J* = 7.3 Hz, 85H, CH_3 TEA) ppm.
^{13}C NMR (101 MHz, D_2O) δ 156.24 (C-2 Uracil), 140.78 (C-6 Uracil), 102.95 (C-5 Uracil), 95.52 (d, *J* = 3.8 Hz, C-1" Rha), 88.41 (C-1' Rib), 82.85 (d, *J* = 9.2 Hz, C-4' Rib), 73.70 (C-2' Rib), 72.69 (C-5" Rha), 72.12 (C-4" Rha), 71.64 (C-3" Rha), 70.79 (d, *J* = 6.0 Hz, C-2" Rha), 69.66 (C-3' Rib), 64.97 (d, *J* = 5.7 Hz, C-5' Rib), 62.45, 46.58 (**C**H_2 TEA), 16.72 (**C**H_3, C-6" Rha), 8.29 (**C**H_3 TEA) ppm.
^{31}P NMR (162 MHz, D_2O) δ -11.45 (d, *J* = 21.0 Hz), -13.69 (d, *J* = 20.6 Hz) ppm.
ESI-TOF-MS (m/z) [M-H]$^-$ calculated for $C_{15}H_{23}N_2O_{16}P_2$ 549.0528; found: 549.0532

GDP-β-L-rhamnose (13)
^{1}H NMR (400 MHz, D_2O) δ 8.10 (s, 1H, H-8 Guanine), 5.94 (d, *J* = 6.3 Hz, 1H, 1H, H-1' Rib), 5.22 (dd, *J* = 8.8, 1.0 Hz, 1H, H-1" Rha), 4.83 (t, d = 5.9 Hz, 1H, H-2' Rib), [11, 12] 4.53 (dd, *J* = 5.2, 3.1 Hz, 1H, H-3' Rib), 4.35 (dt, *J* = 5.4, 2.6 Hz, 1H, H-4' Rib), 4.20 (dd, *J* = 5.5, 3.5 Hz, 2H, H-5' Rib), 4.09 (d, *J* = 3.3 Hz, 1H, H-2" Rha), 3.62 (dd, *J* = 9.2, 3.3 Hz, 1H, H-3" Rha), 3.45 – 3.32 (m, 2H, H-4" & H-5" Rha,), 3.19 (q, *J* = 7.3 Hz, 24H), 1.28 (t, *J* = 7.3 Hz, 39H, CH_3 of TEA and H-6" Rha) ppm.
^{13}C NMR (101 MHz, D_2O) δ 159.92 (C-6, Guanine), 154.70 (C-2), 151.84 (C-4, Guanine), 137.45 (C-8, Guanine), 116.41 (C-5, Guanine), 95.51 (d, *J* = 3.8 Hz, C-1" Rha), 86.63 (C-1' Rib), 83.81 (d, *J* = 9.1 Hz, C-4' Rib), 73.49 (C-2' Rib), 72.68 (C-5" Rha), 72.10 (C-4" Rha), 71.62 (C-3" Rha), 70.77 (d, *J* = 5.6 Hz, C-2" Rha), 70.48 (C-3' Rib), 65.26 (d, *J* = 5.8 Hz, C-5' Rib), 46.63 (**C**H_2 TEA), 16.67 (**C**H_3, C-6" Rha), 8.20 (**C**H_3 TEA) ppm.
^{31}P NMR (162 MHz, D_2O) δ -11.40 (d, *J* = 20.6 Hz), -13.67 (d, *J* = 20.6 Hz).
ESI-TOF-MS (m/z) [M-H]$^-$ calculated for $C_{16}H_{24}N_5O_{15}P_2$ 588.0750; found: 588.0797

3 NMR spectra of compounds synthesized in this study.

Compound 6a

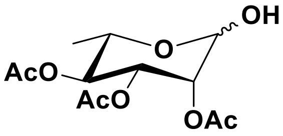

^{1}H NMR

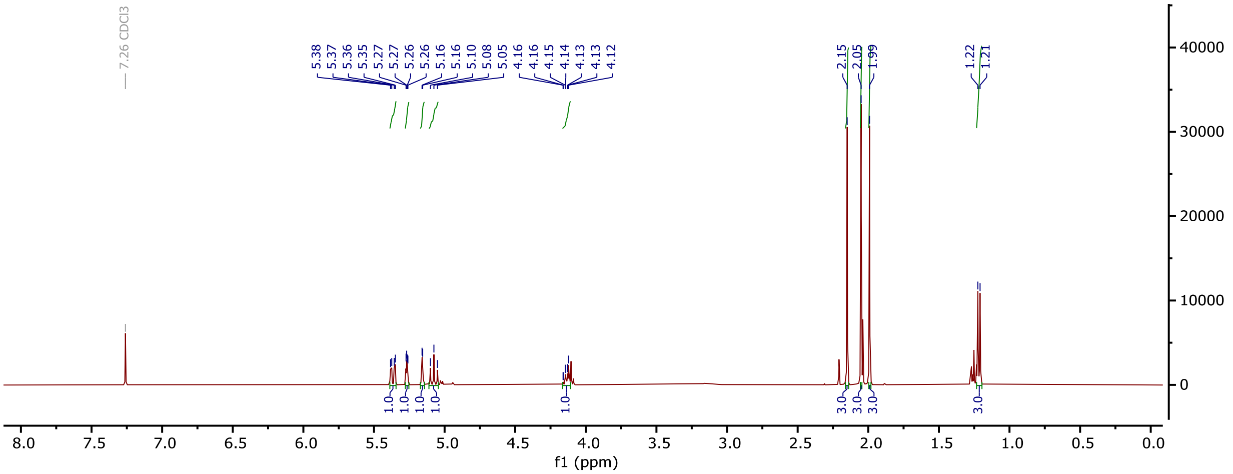

^{13}C NMR

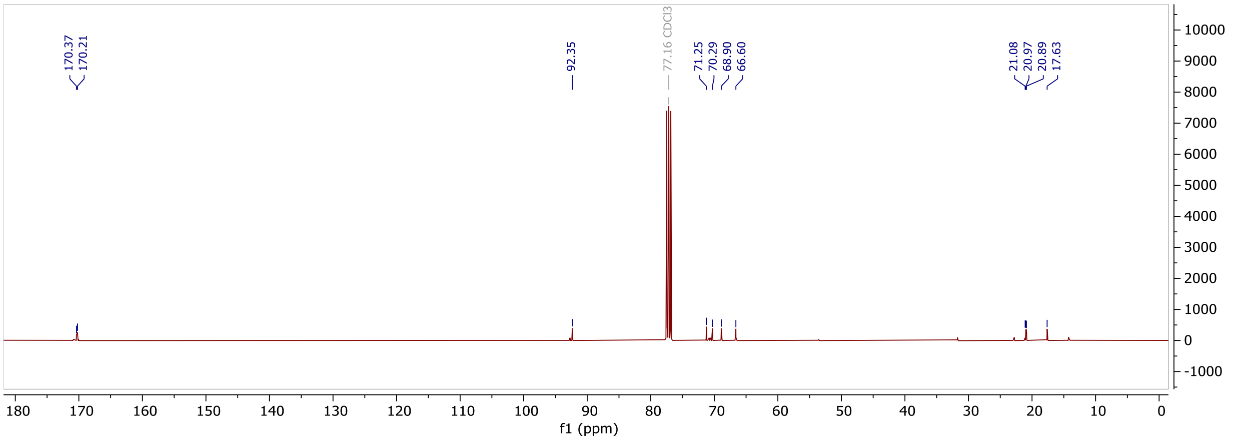

Compound 9a

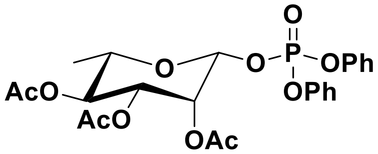

^{1}H NMR

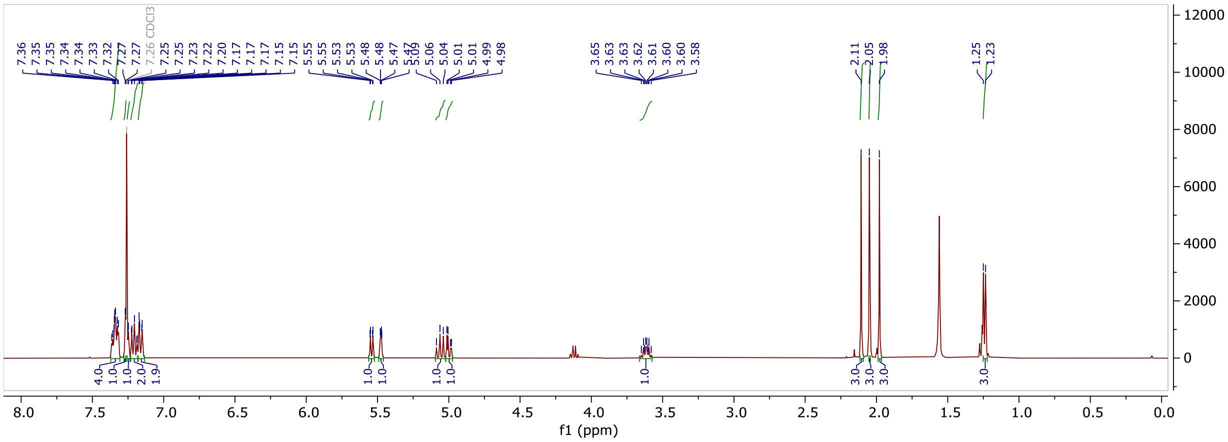

^{13}C NMR

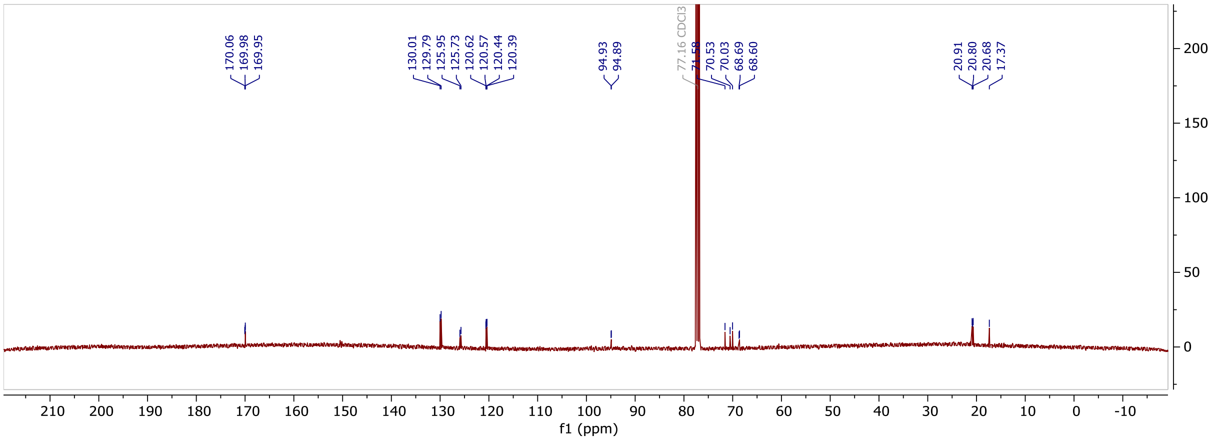

^{31}P NMR

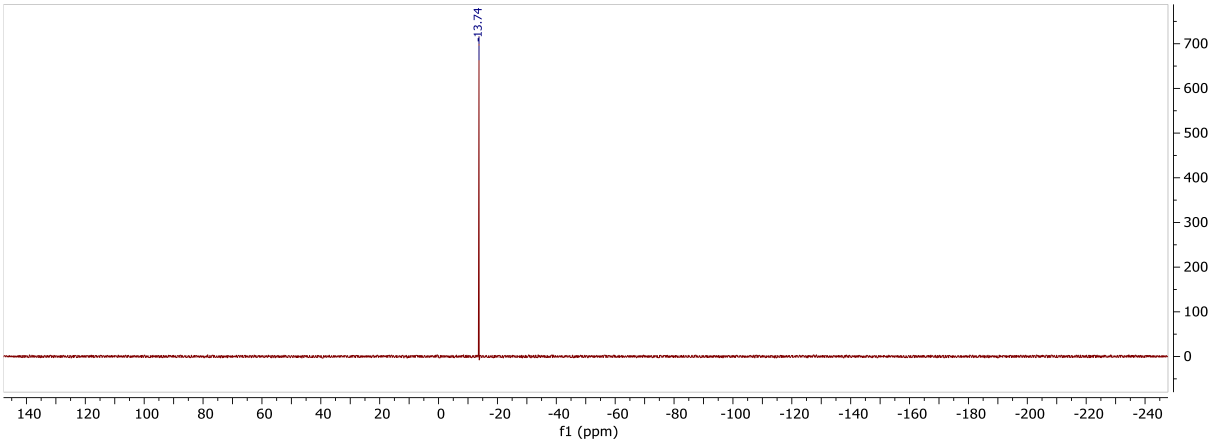

Compound 9a-α

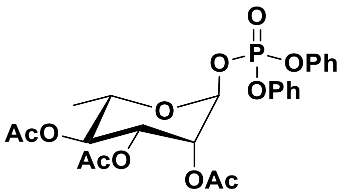

^{1}H NMR

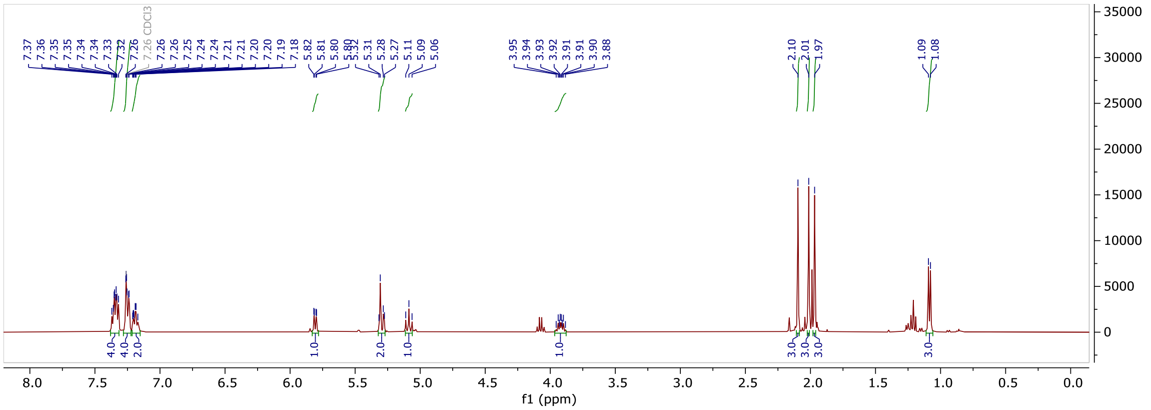

^{13}C NMR

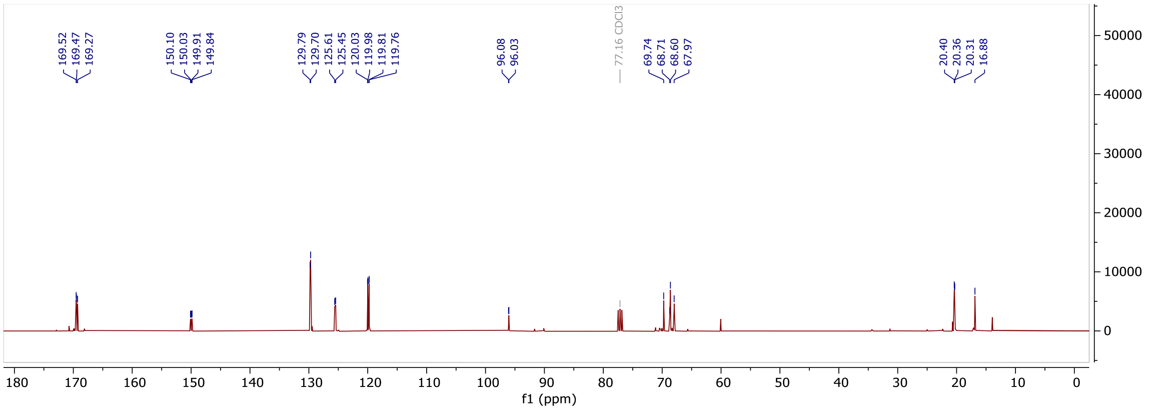

^{31}P NMR

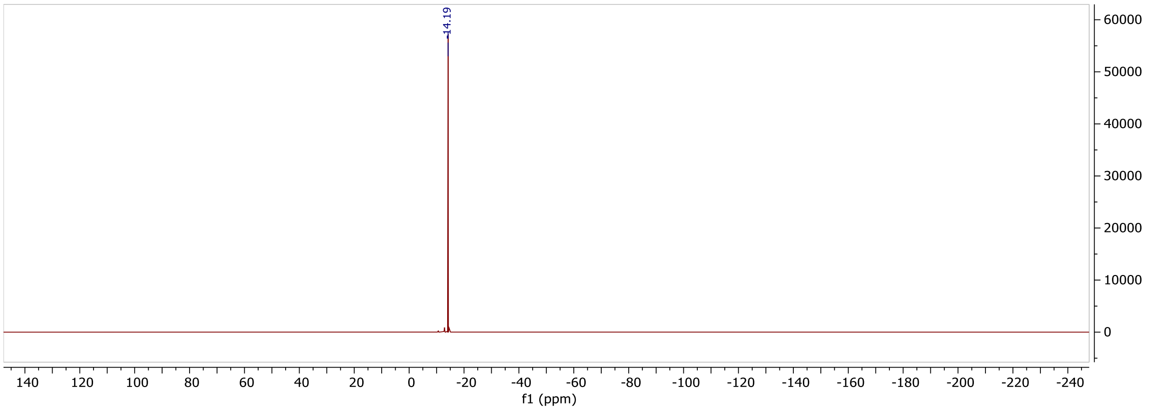

Compound 3

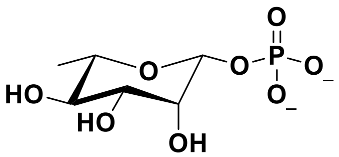

^{1}H NMR

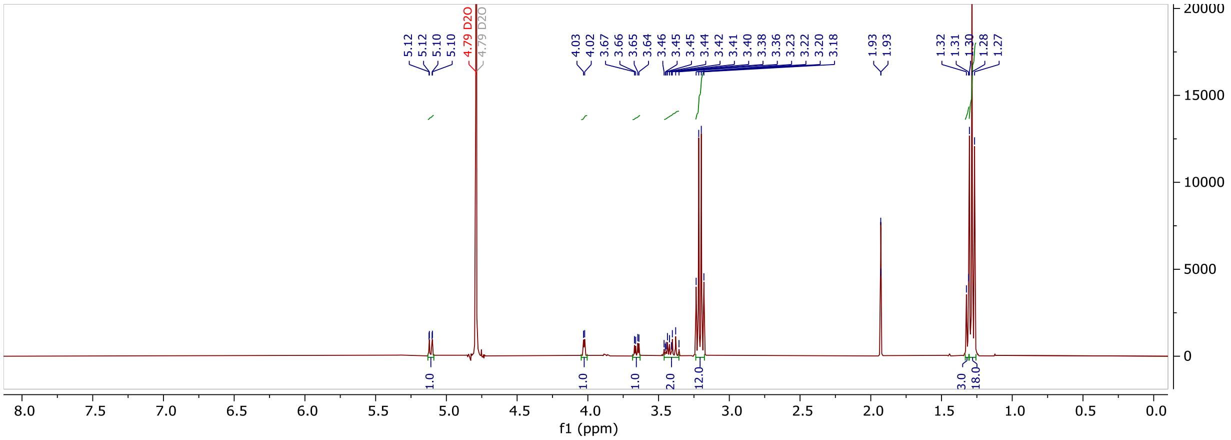

^{13}C NMR

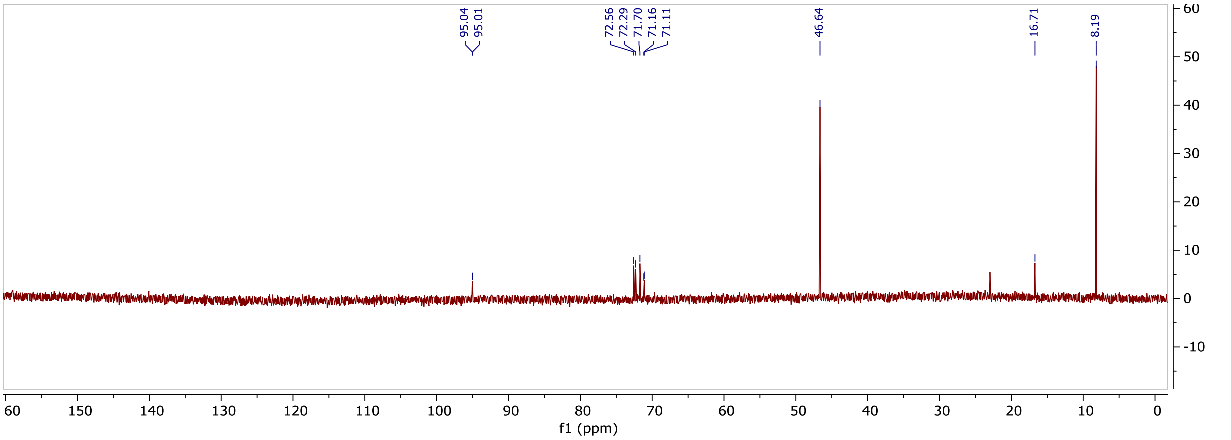

^{31}P NMR

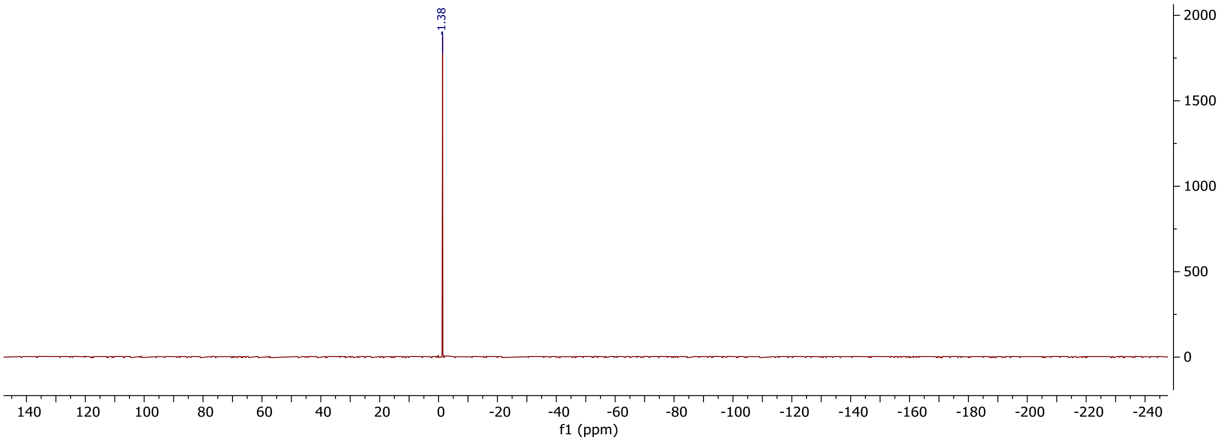

Compound 3-α

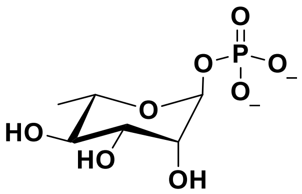

^{1}H NMR

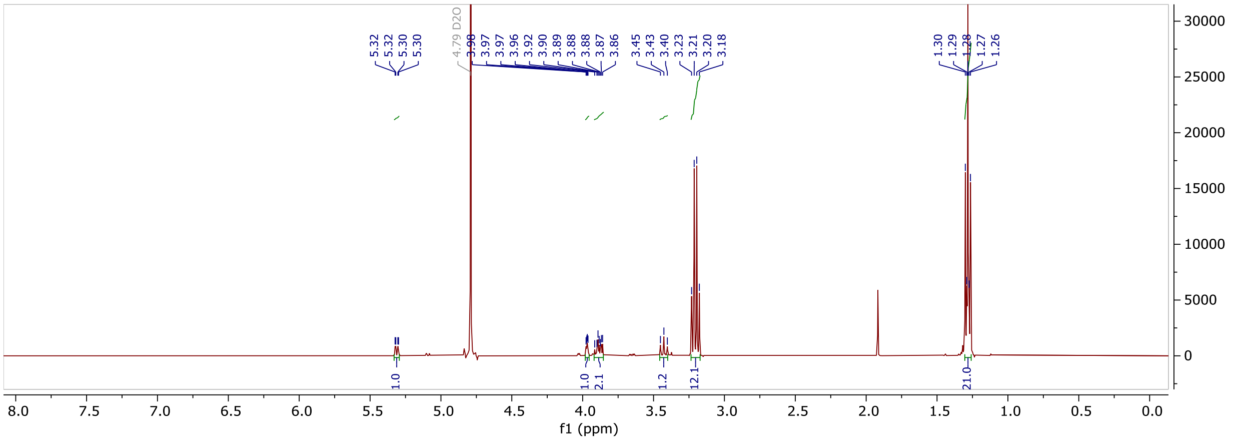

^{13}C NMR

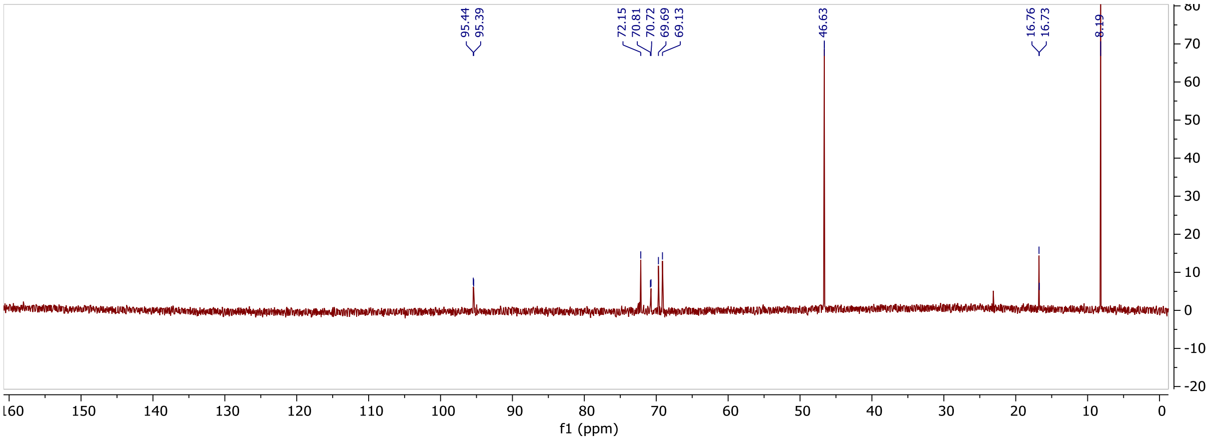

^{31}P NMR

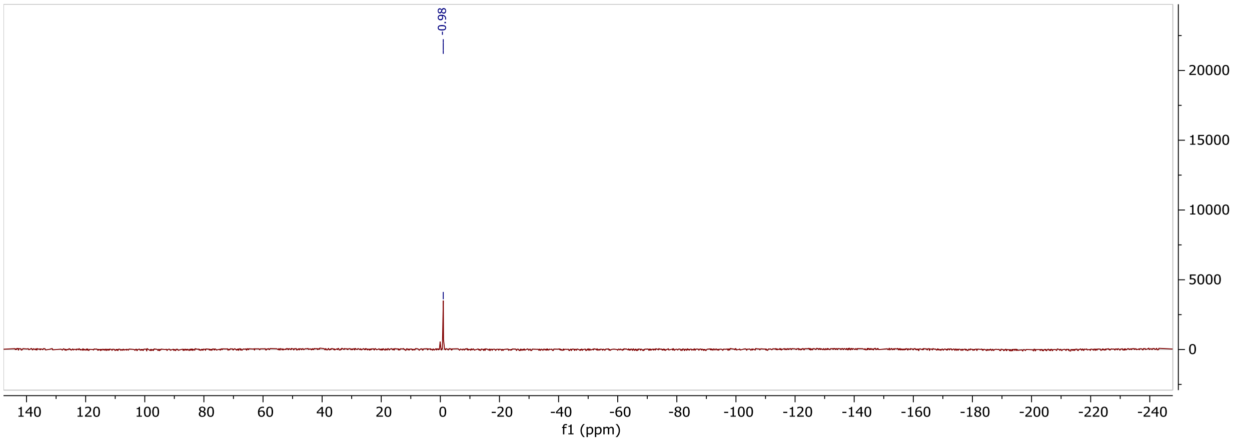

Compound 6b

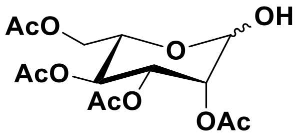

^{1}H NMR

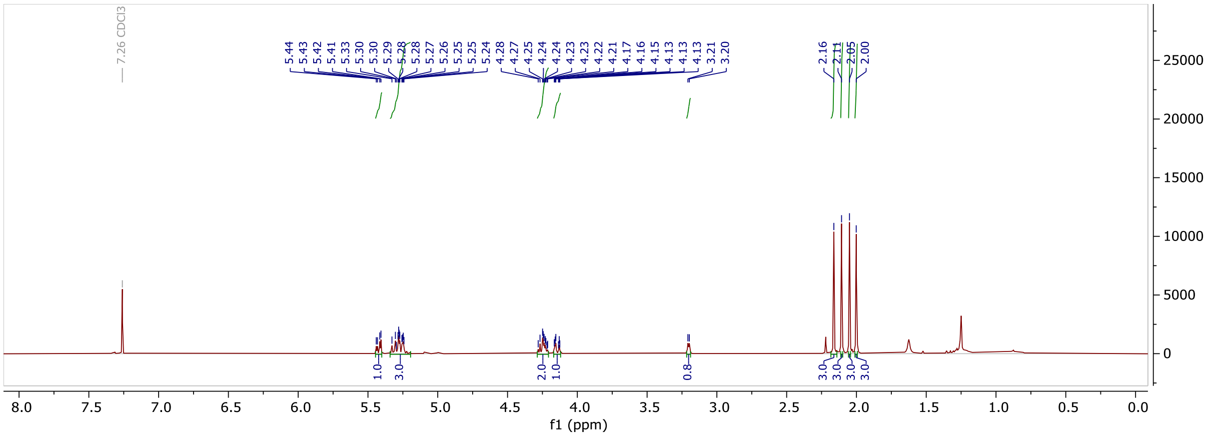

^{13}C NMR

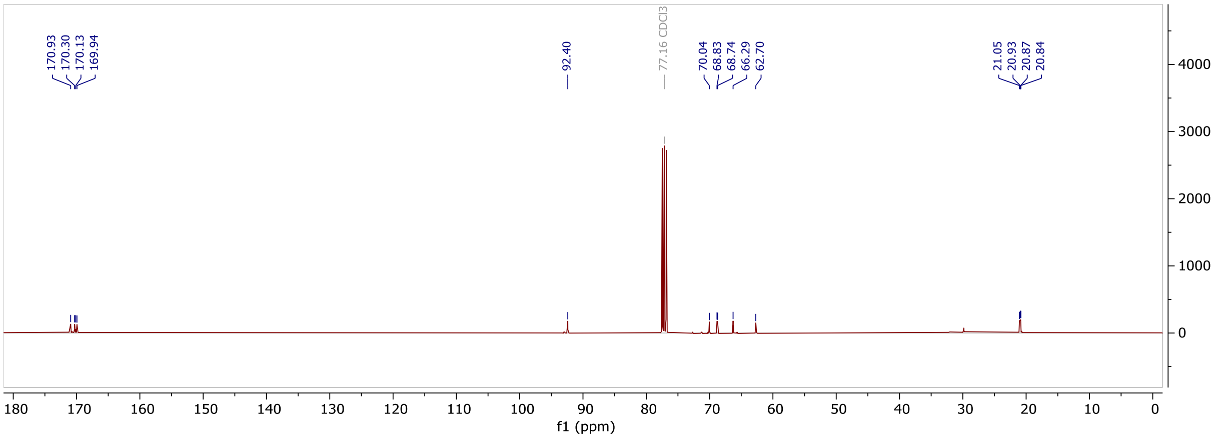

Compound 9b

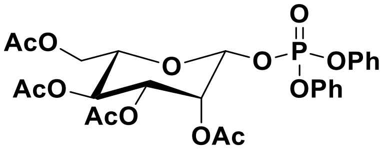

^{1}H NMR

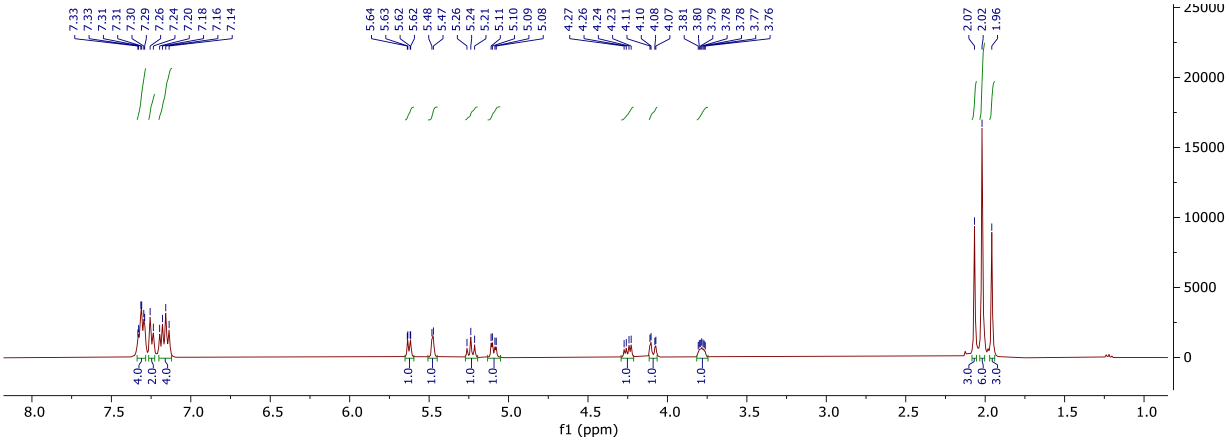

^{13}C NMR

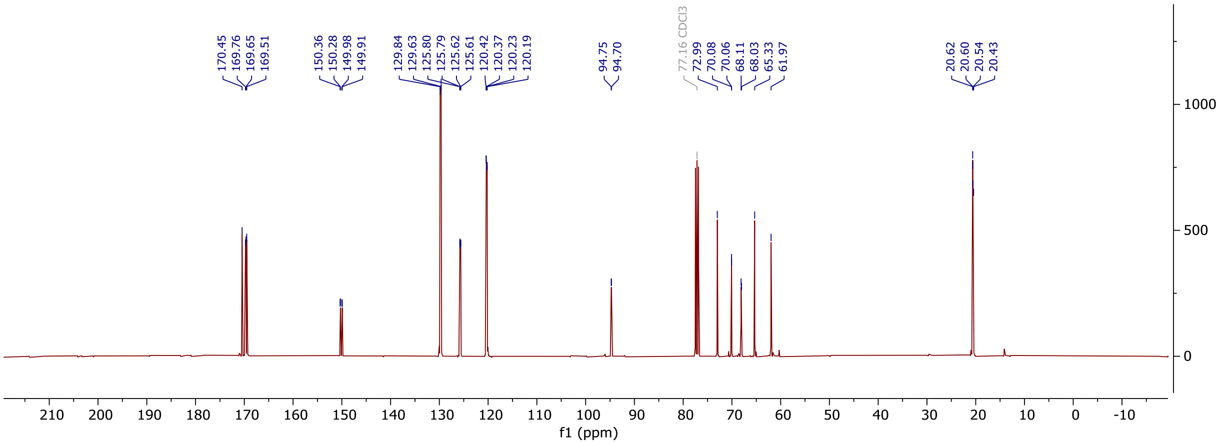

^{31}P NMR

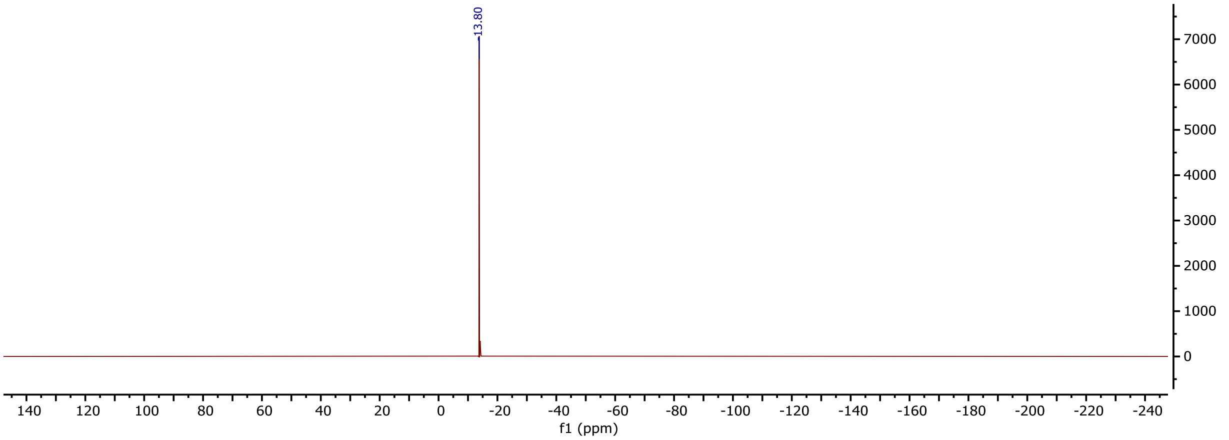

Compound 9b-α

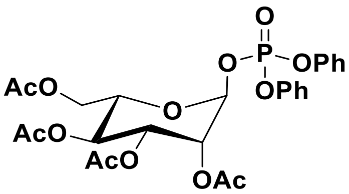

^{1}H NMR

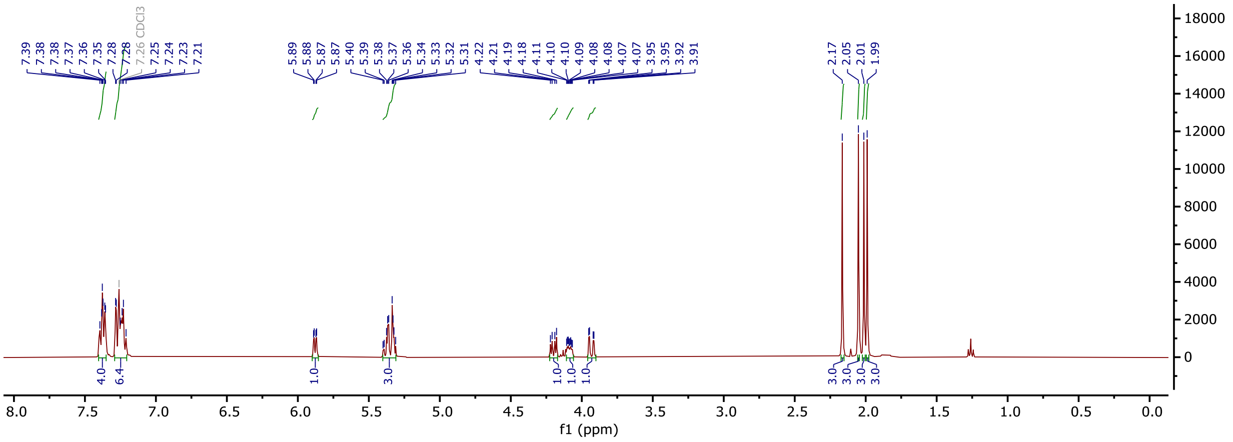

^{13}C NMR

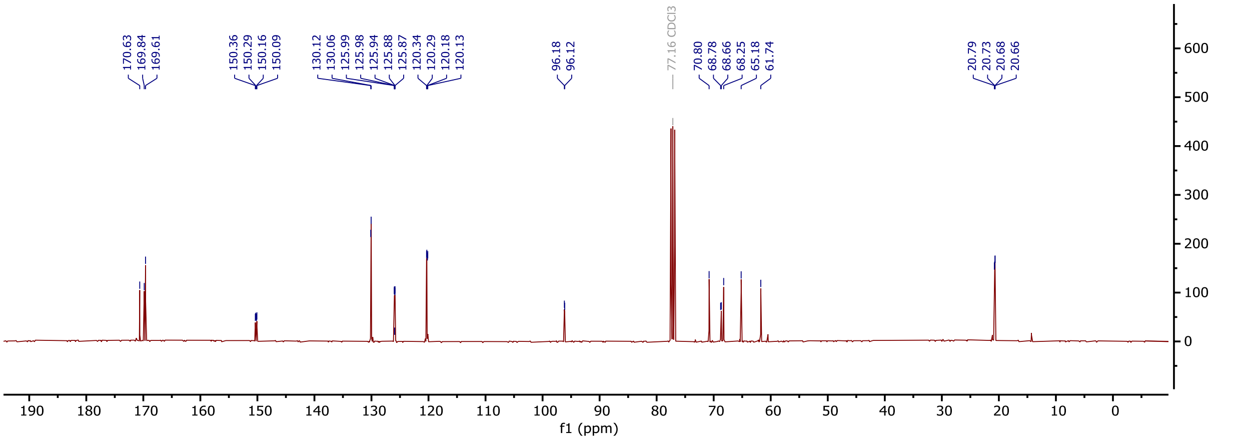

^{31}P NMR

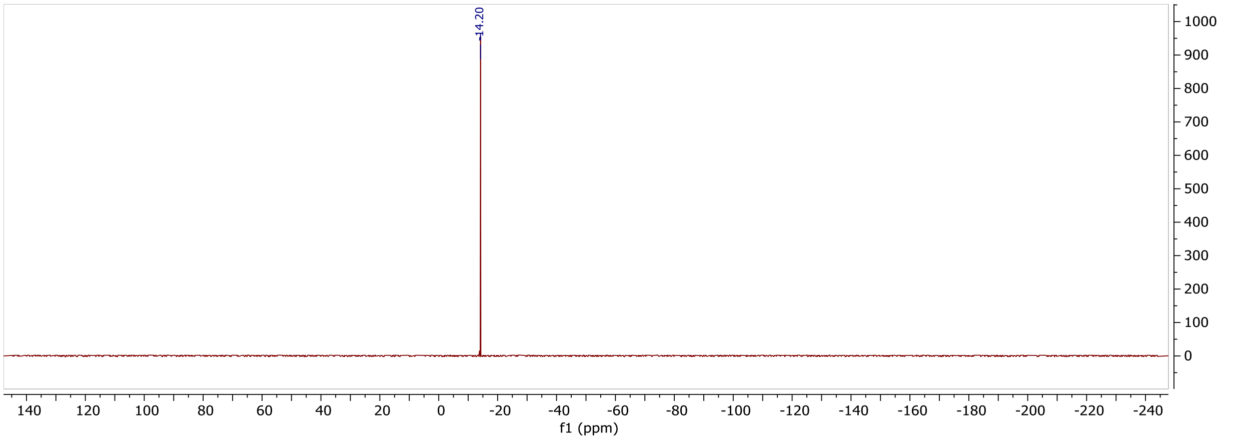

Compound 4

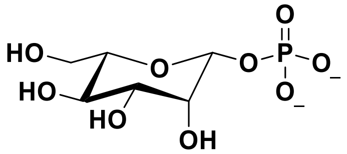

^{1}H NMR

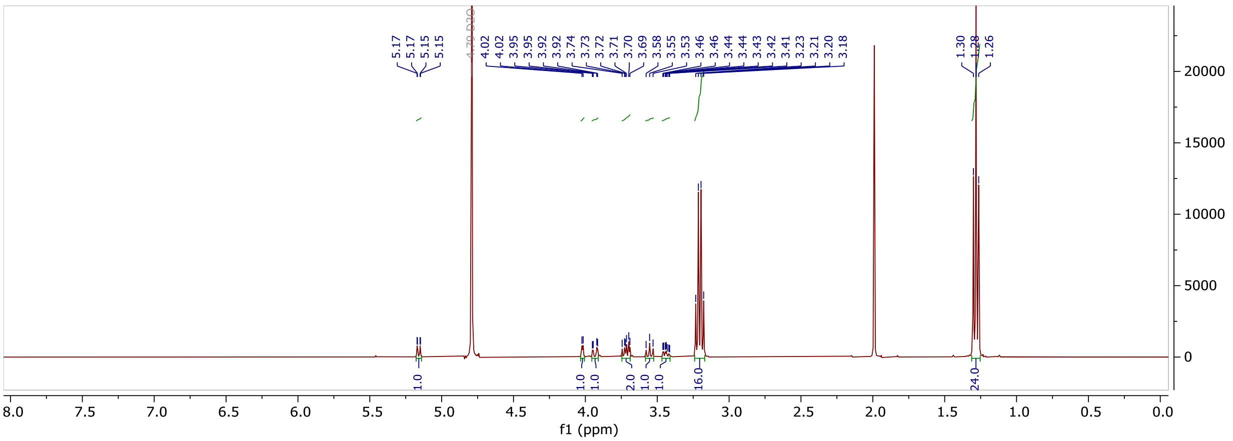

^{13}C NMR

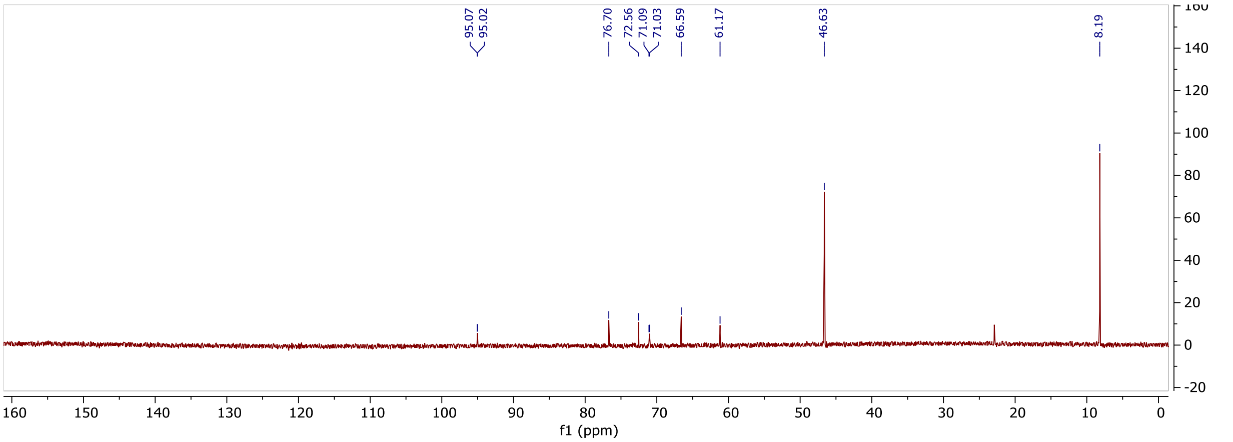

^{31}P NMR

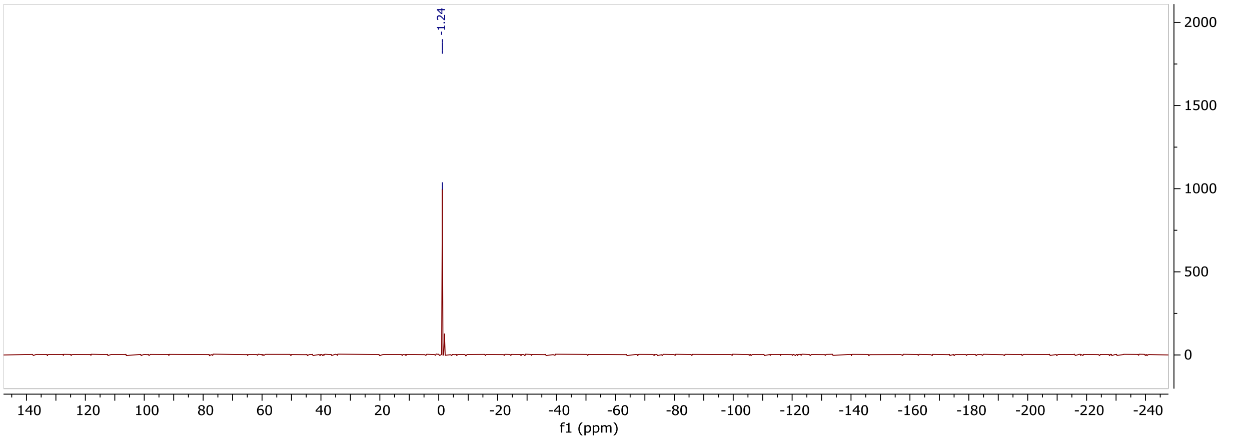

Compound 4-α

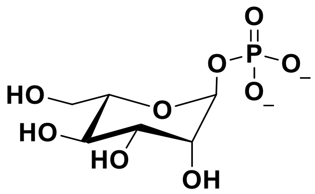

^{1}H NMR

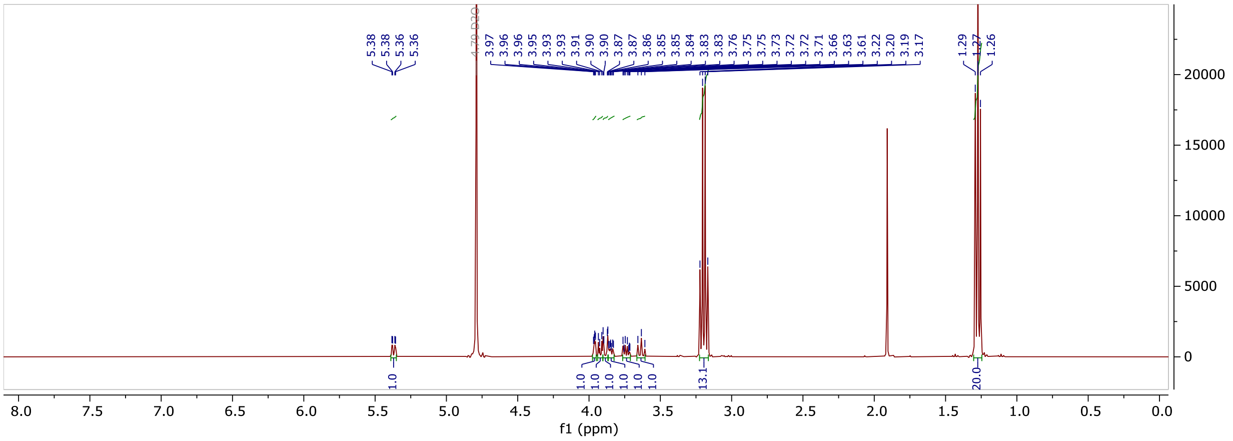

^{13}C NMR

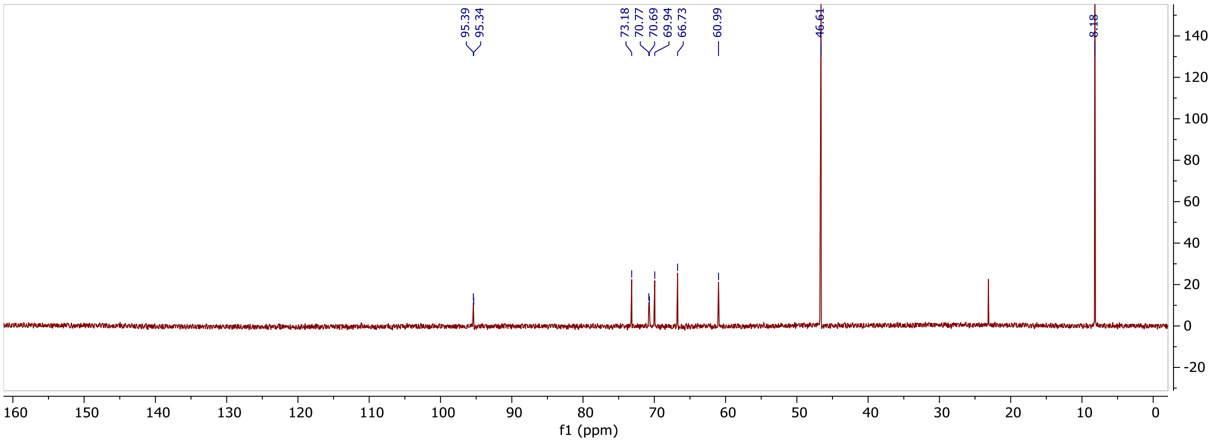

^{31}P NMR

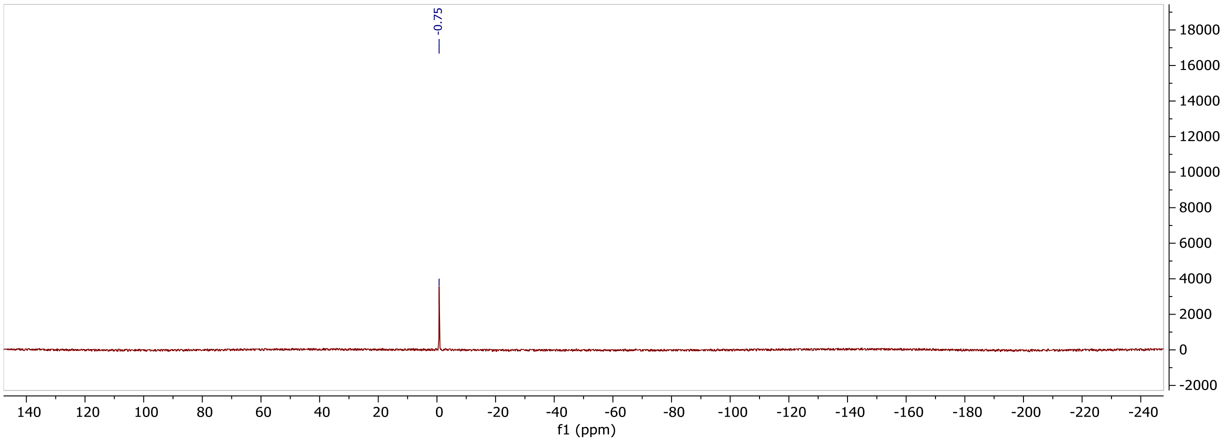

Compound 7

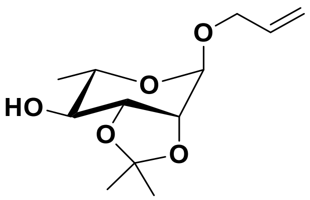

^{1}H NMR

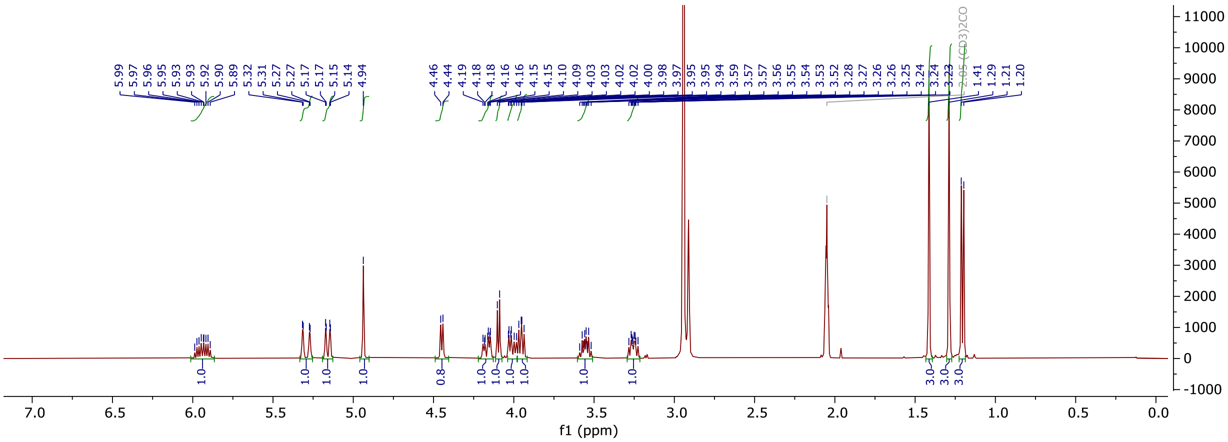

^{13}C NMR

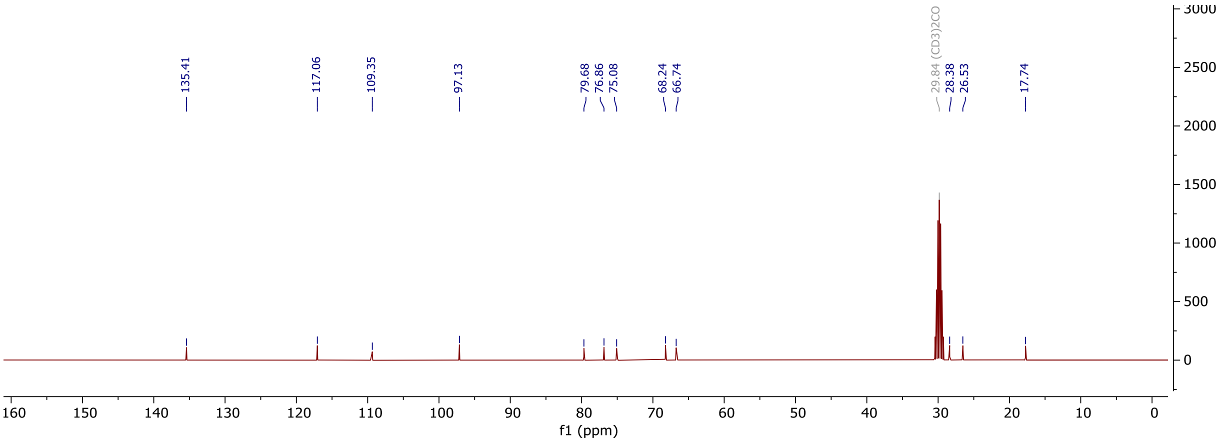

Compound 8

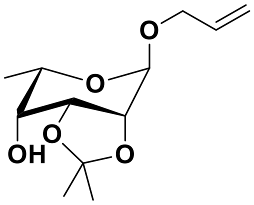

^{1}H NMR

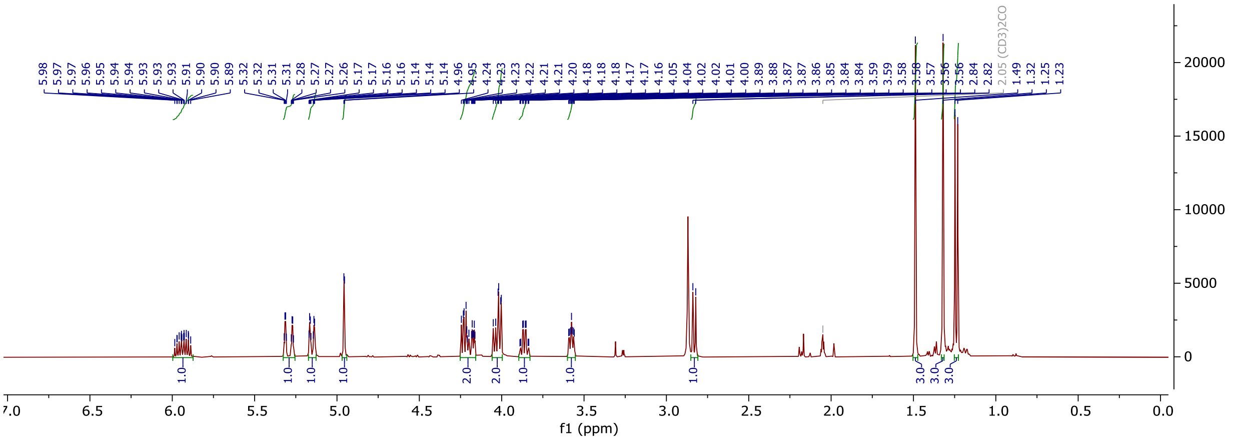

^{13}C NMR

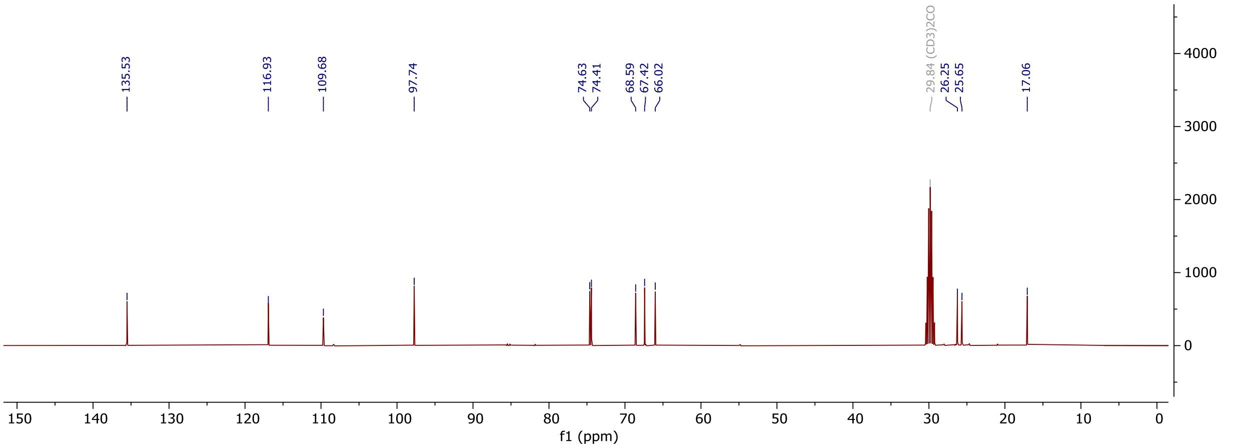

Compound S2

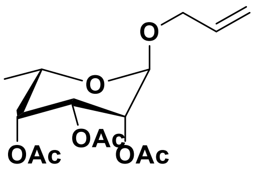

^{1}H NMR

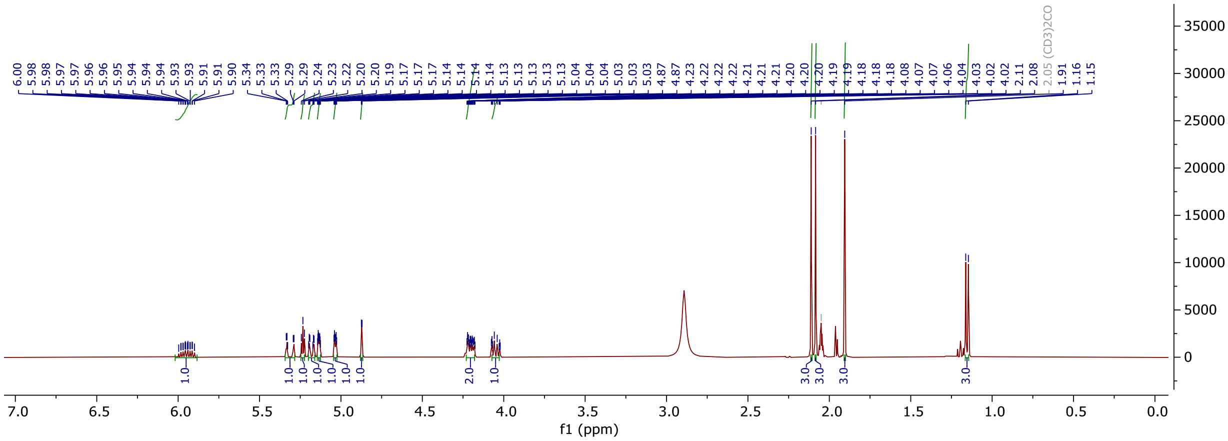

^{13}C NMR

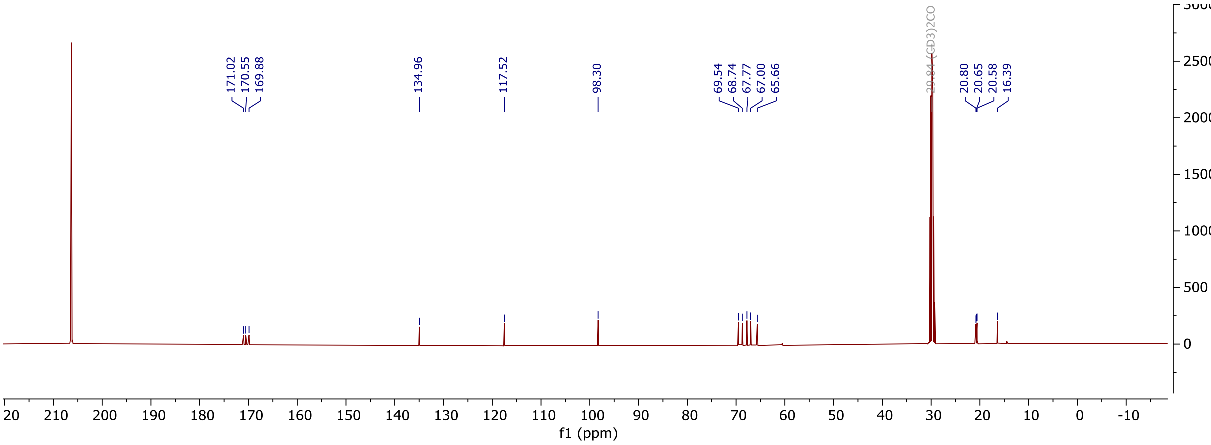

Compound 6c

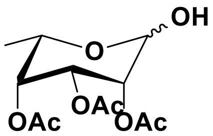

^{1}H NMR

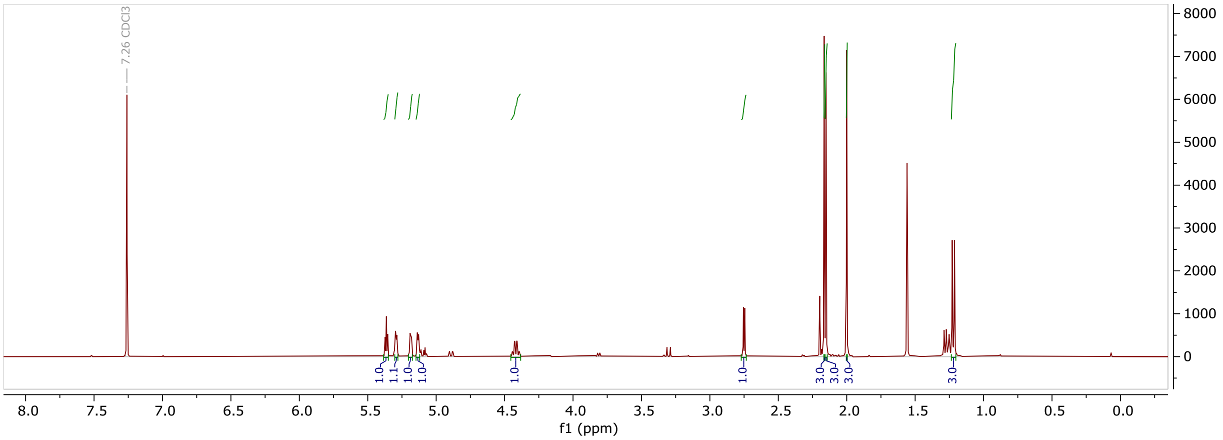

^{13}C NMR

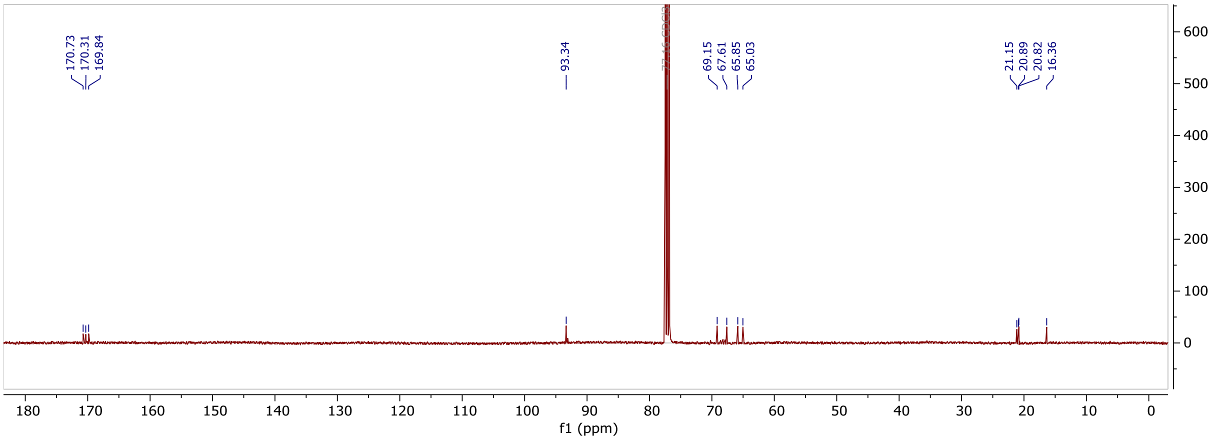

Compound 9c

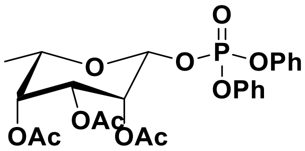

^{1}H NMR

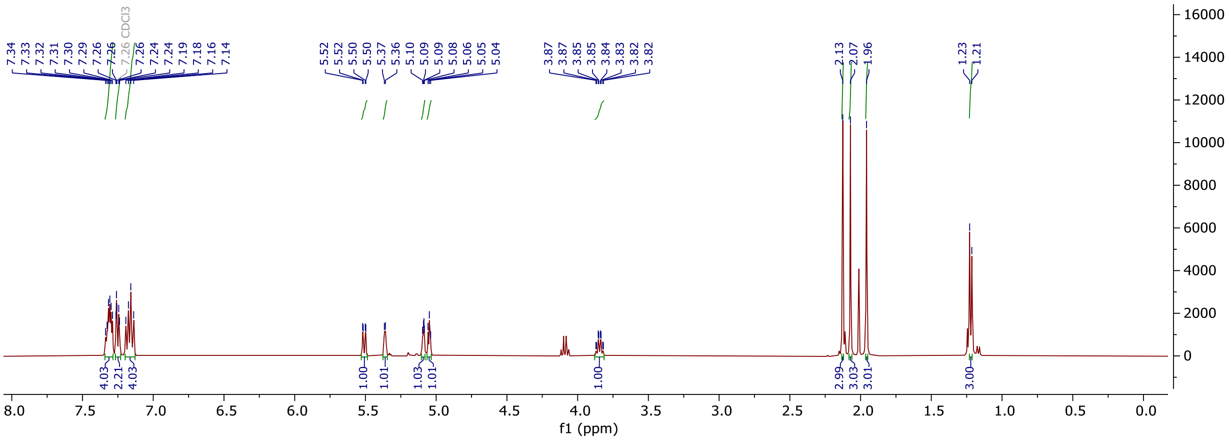

^{13}C NMR

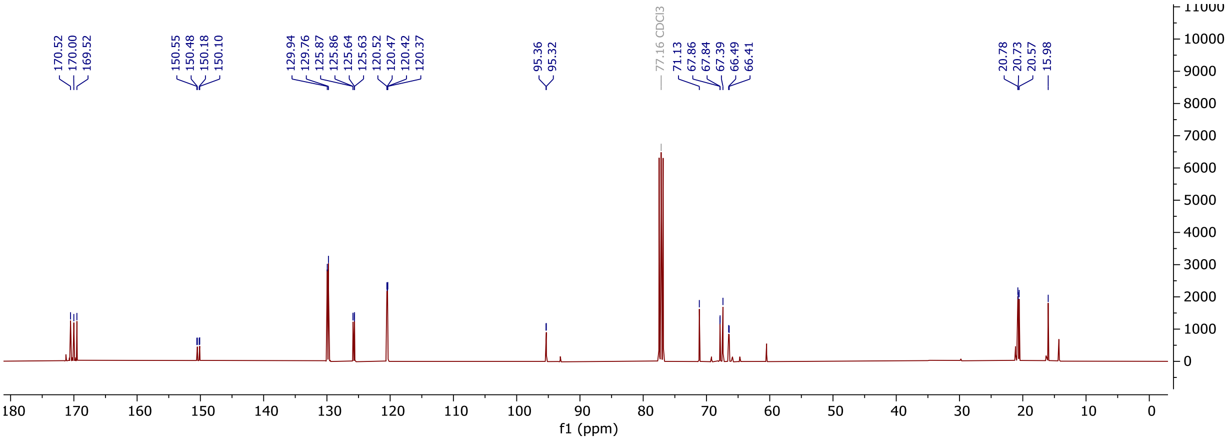

^{31}P NMR

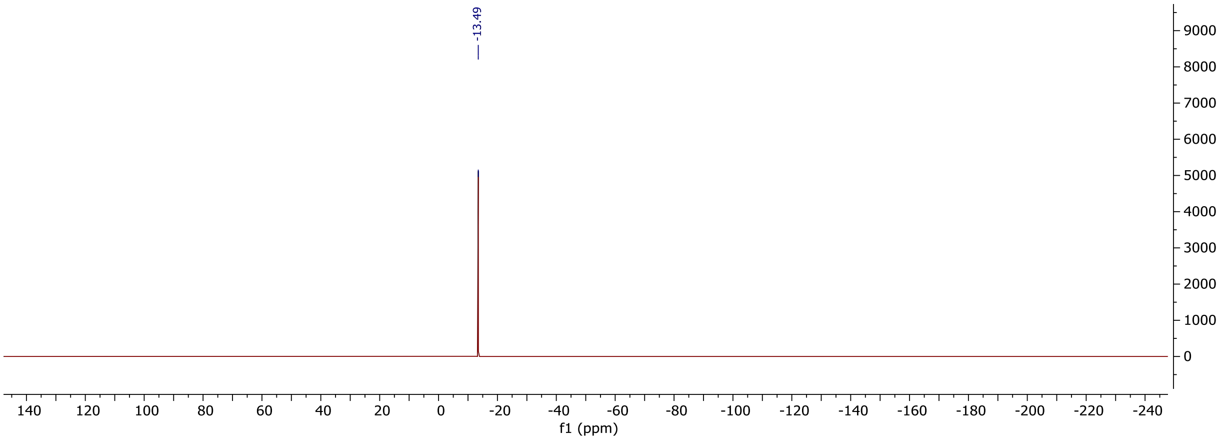

Compound 5

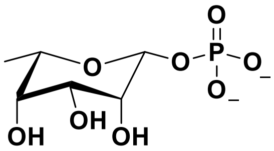

^{1}H NMR

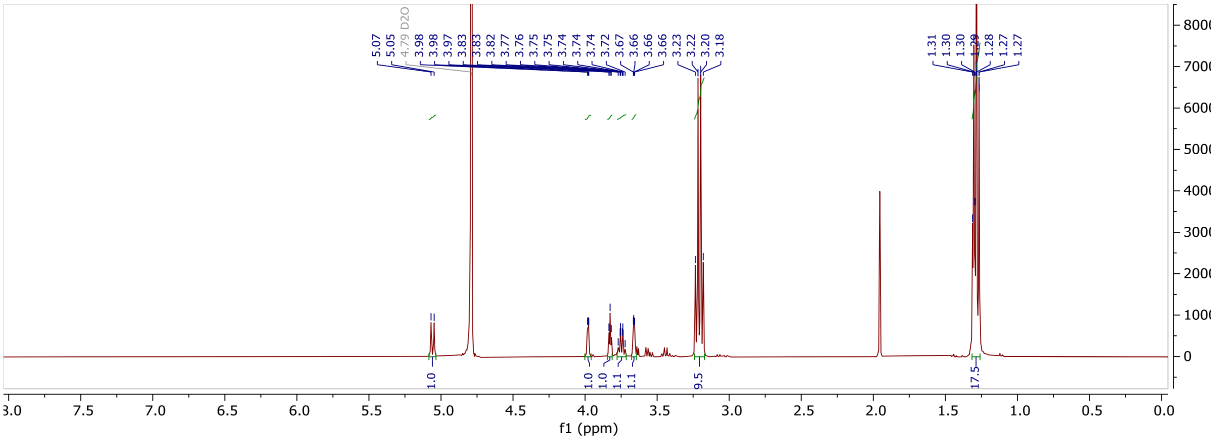

^{13}C NMR

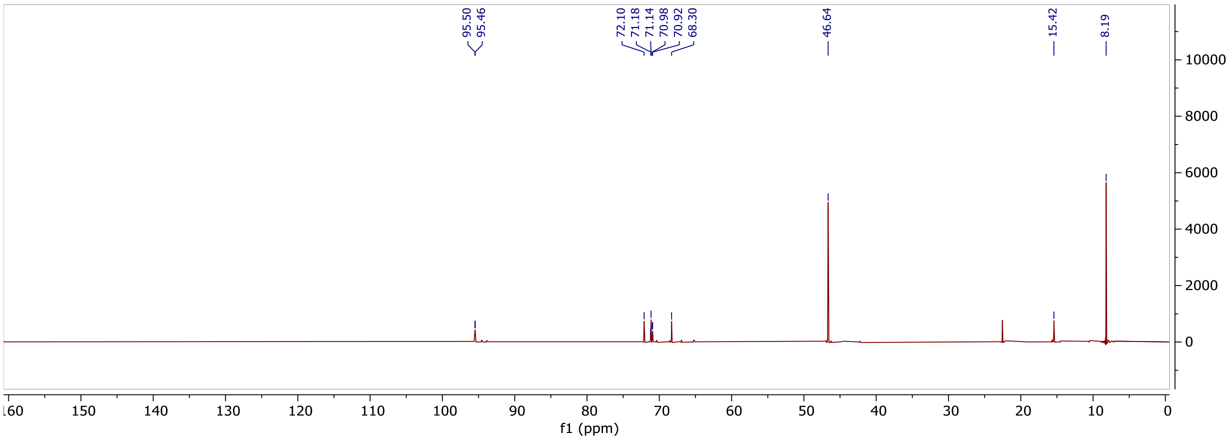

^{31}P NMR

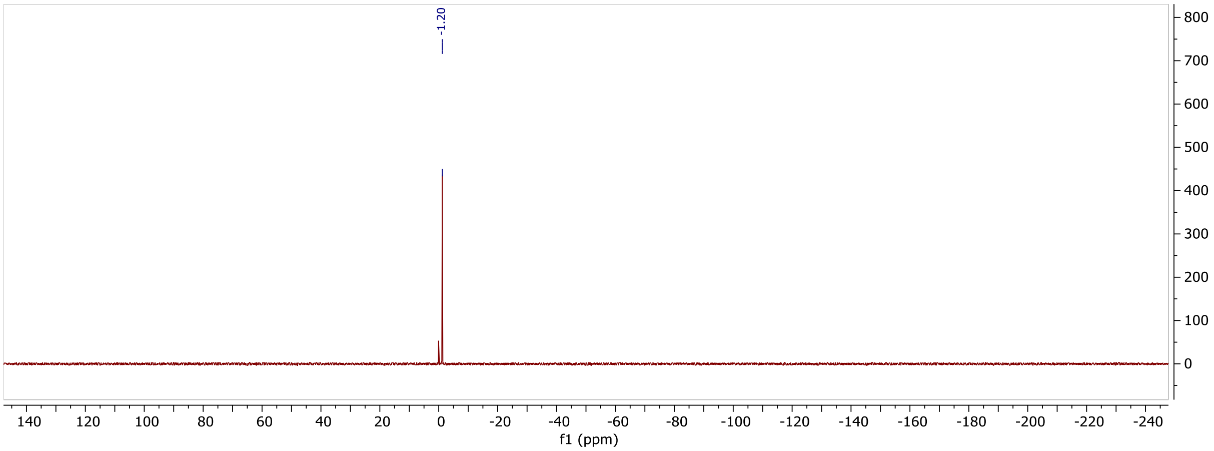

dTDP-β-L-Rhamnose (1)

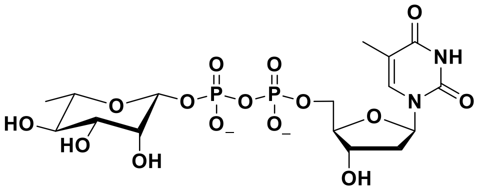

^{1}H NMR

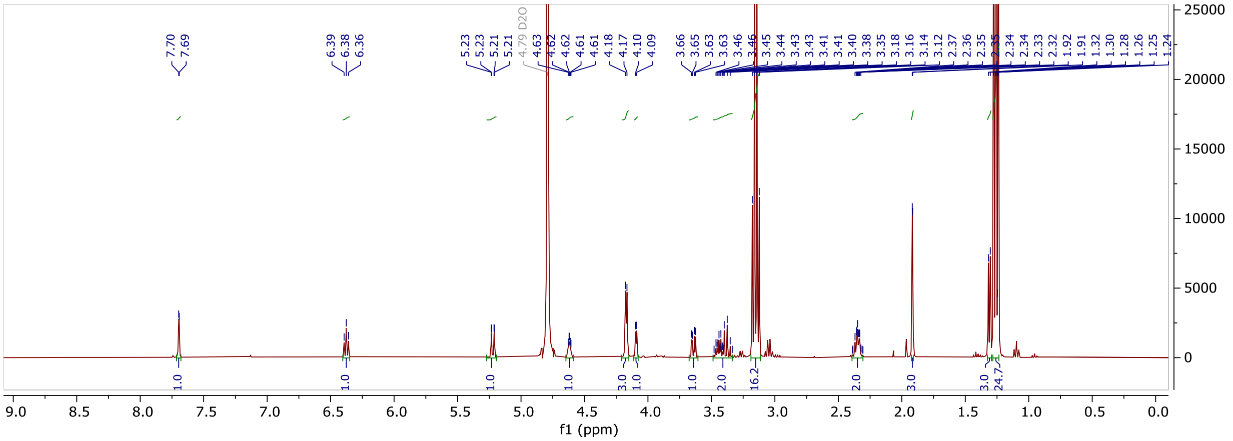

^{13}C NMR

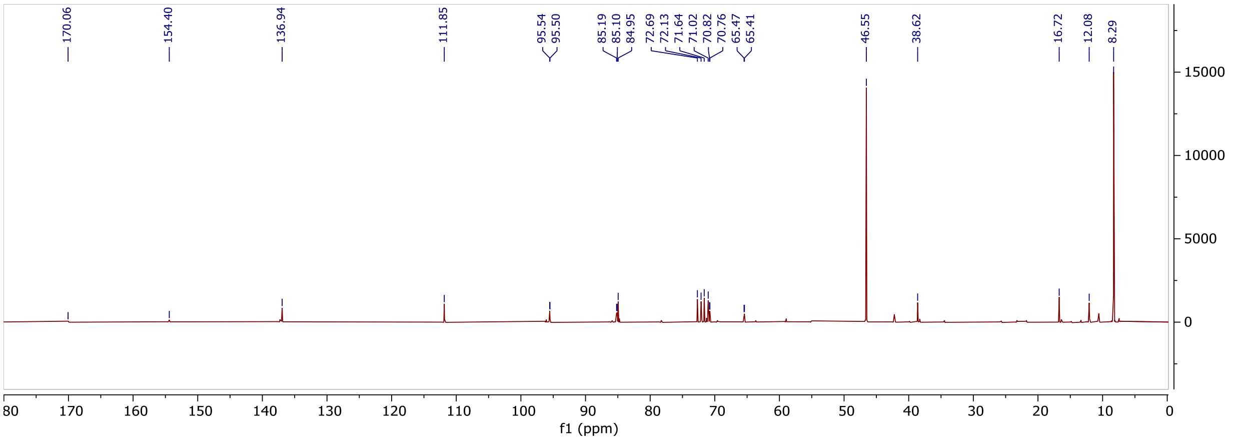

^{31}P NMR

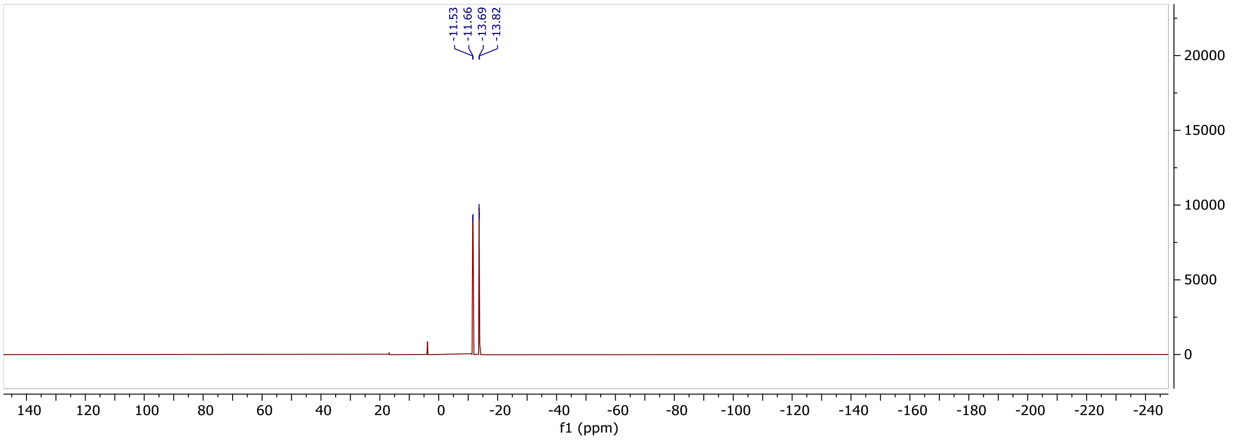

dTDP-α-L-Rhamnose (10)

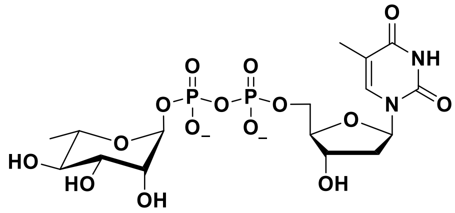

^{1}H NMR

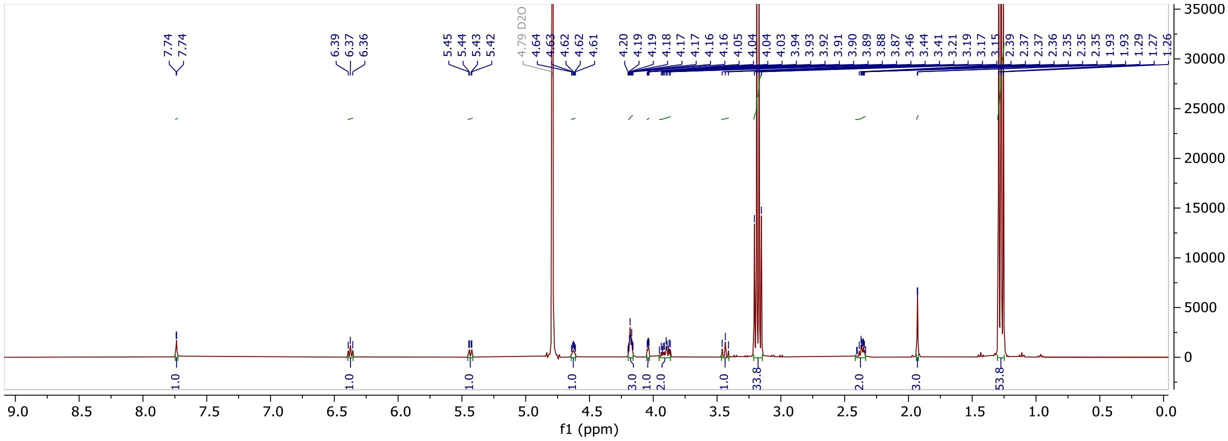

^{13}C NMR

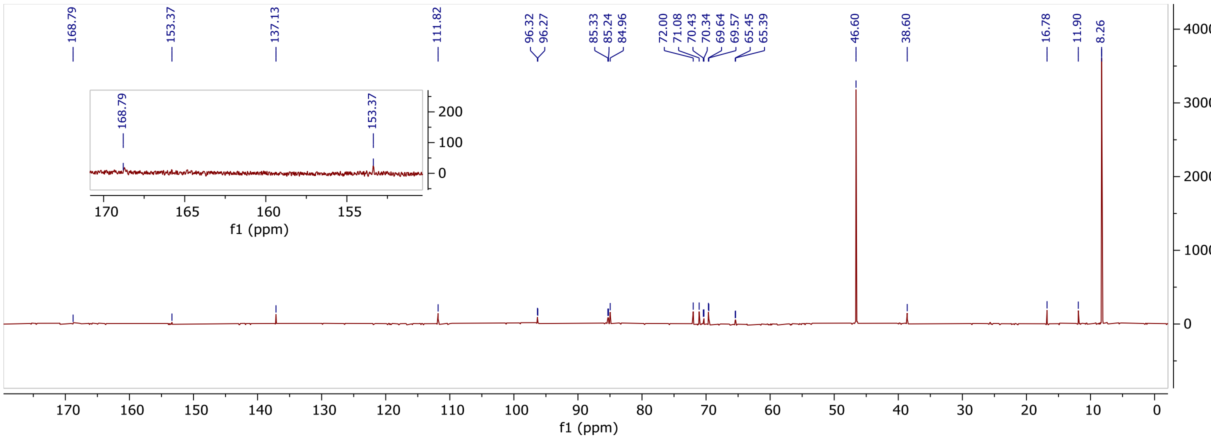

^{31}P NMR

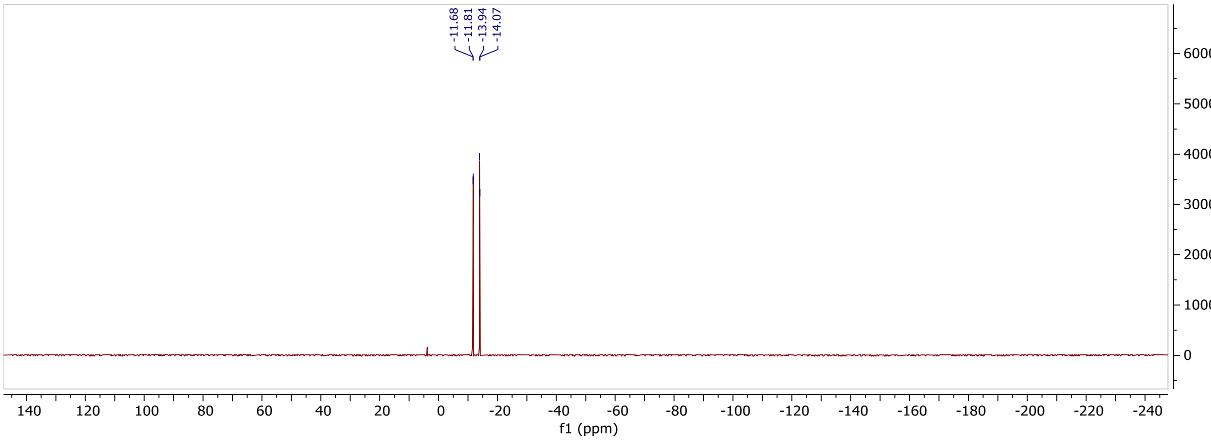

dTDP-β-L-Mannose (11)

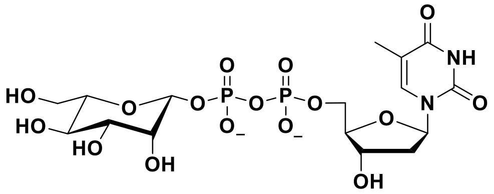

^{1}H NMR

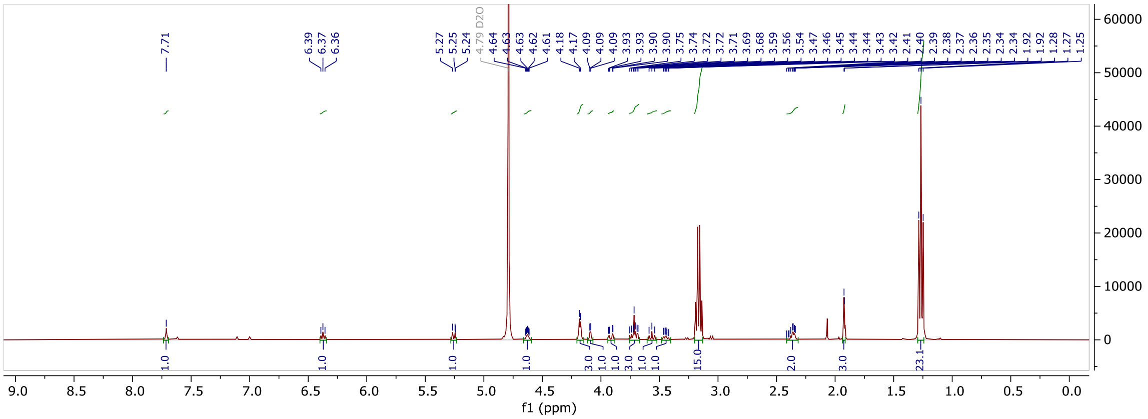

^{13}C NMR

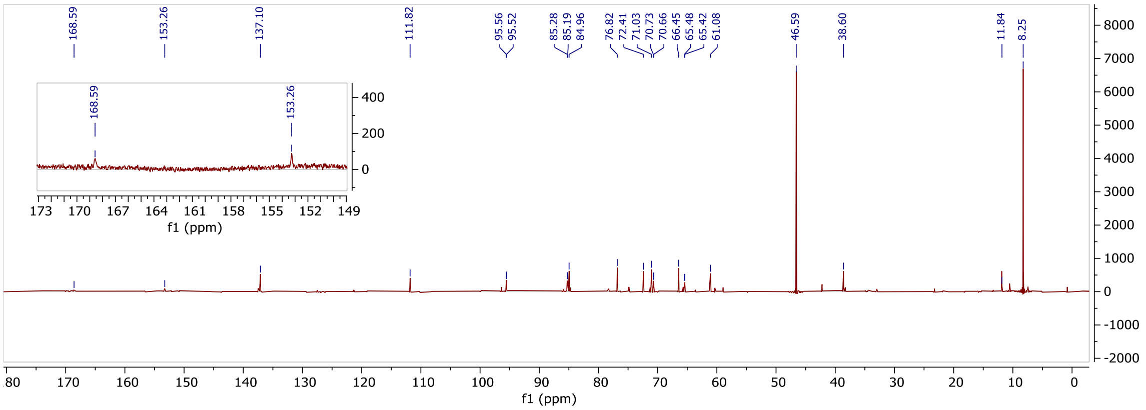

^{31}P NMR

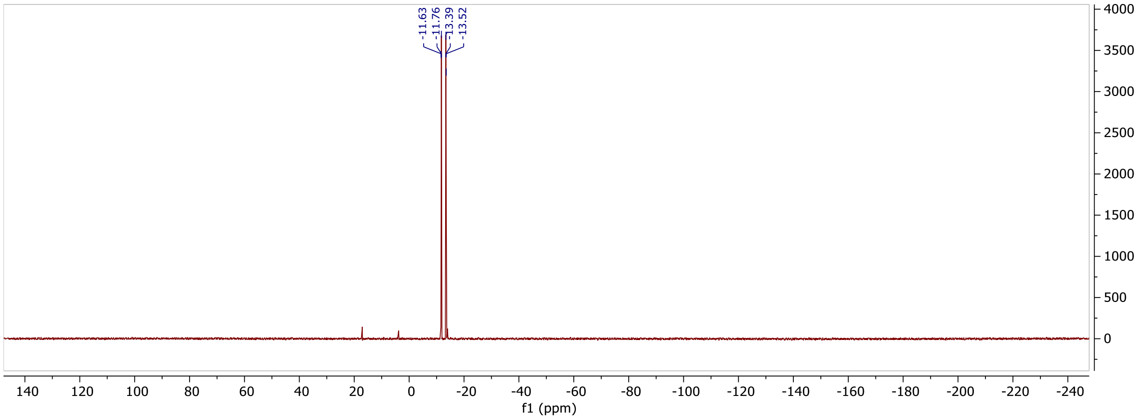

dTDP-6-deoxy-β-L-Talose (2)

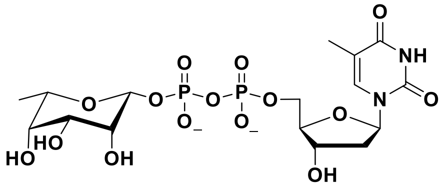

^{1}H NMR

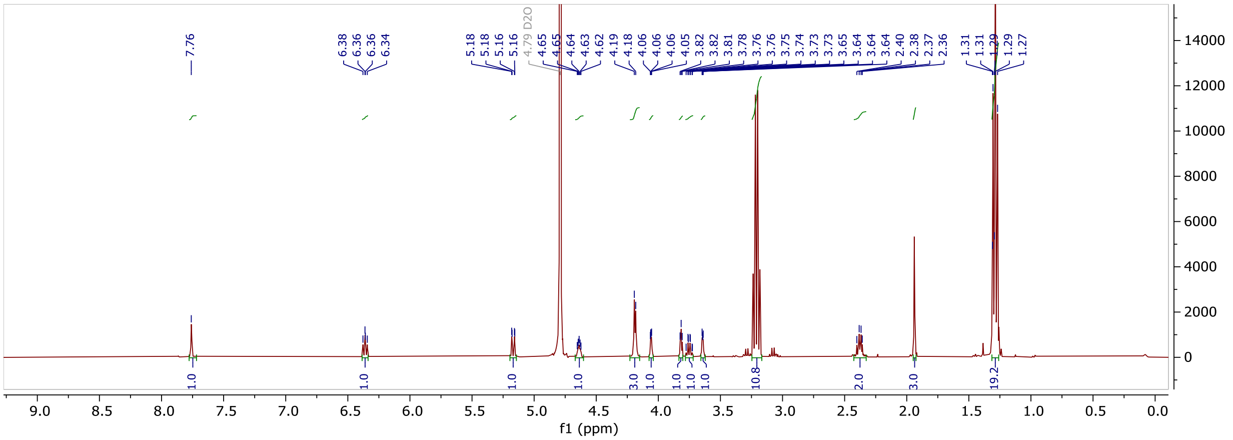

^{13}C NMR

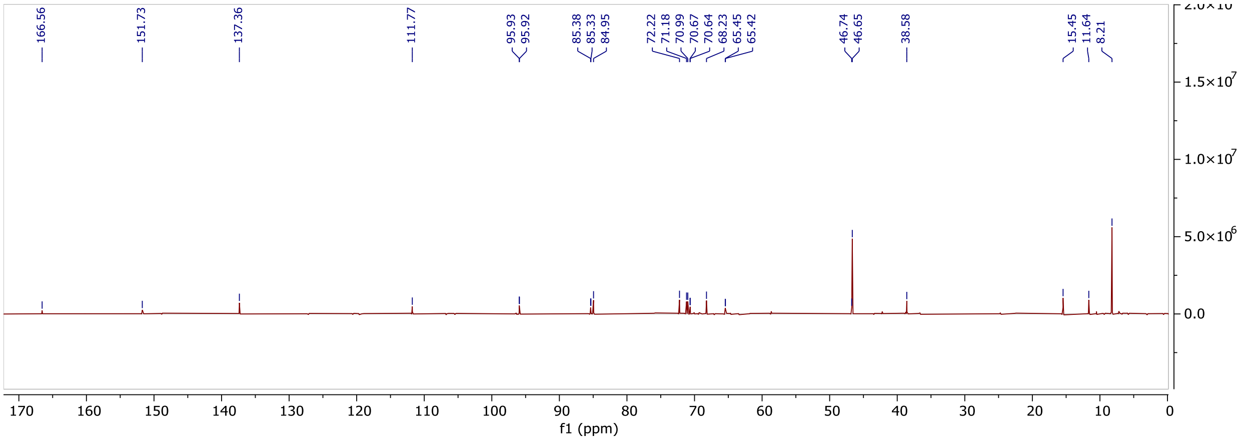

^{31}P NMR

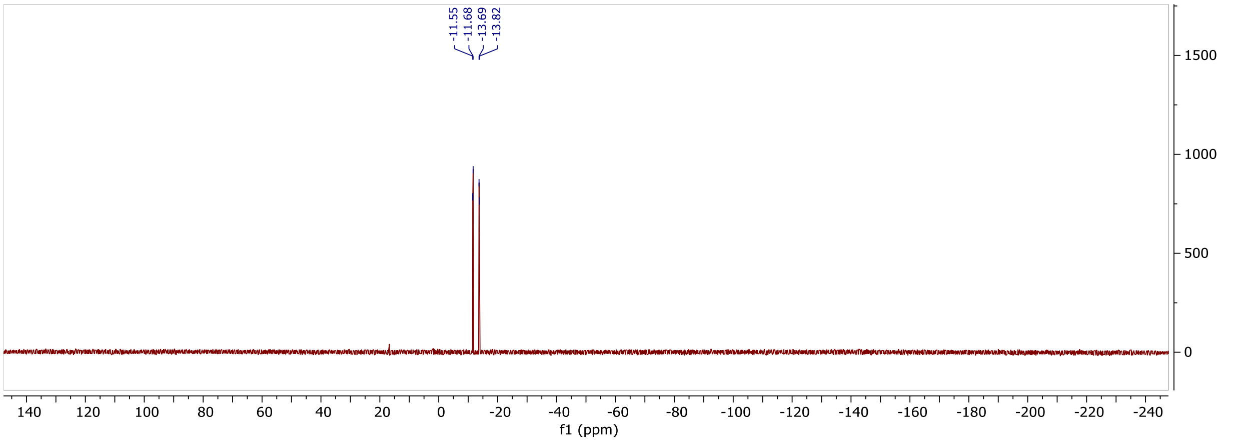

UDP-β-L-Rhamnose (12)

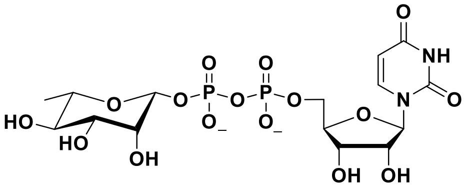

^{1}H NMR

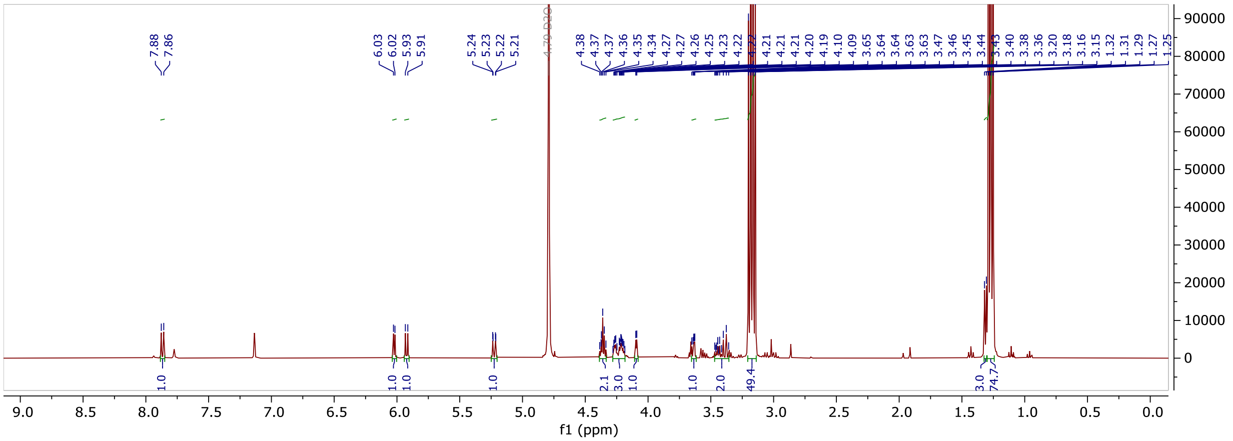

^{13}C NMR

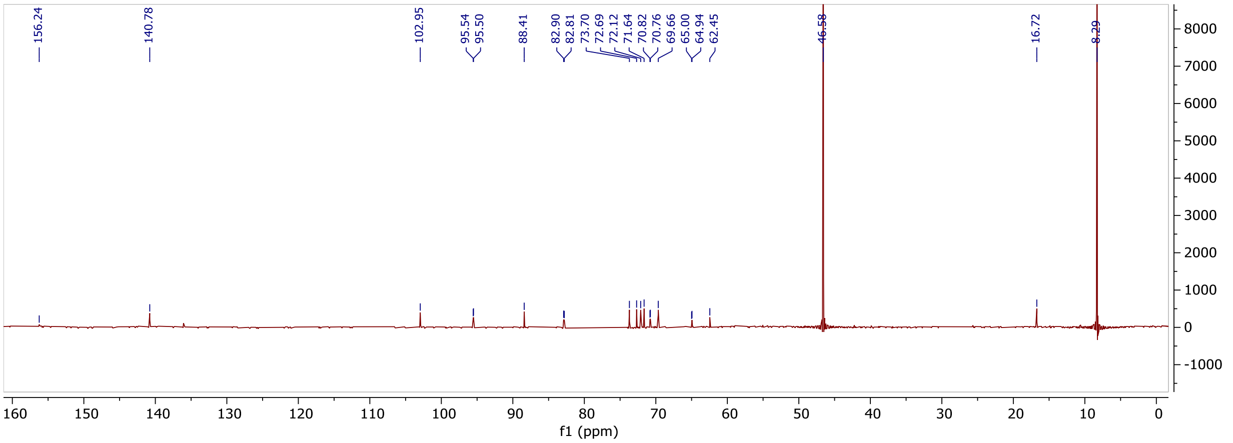

^{31}P NMR

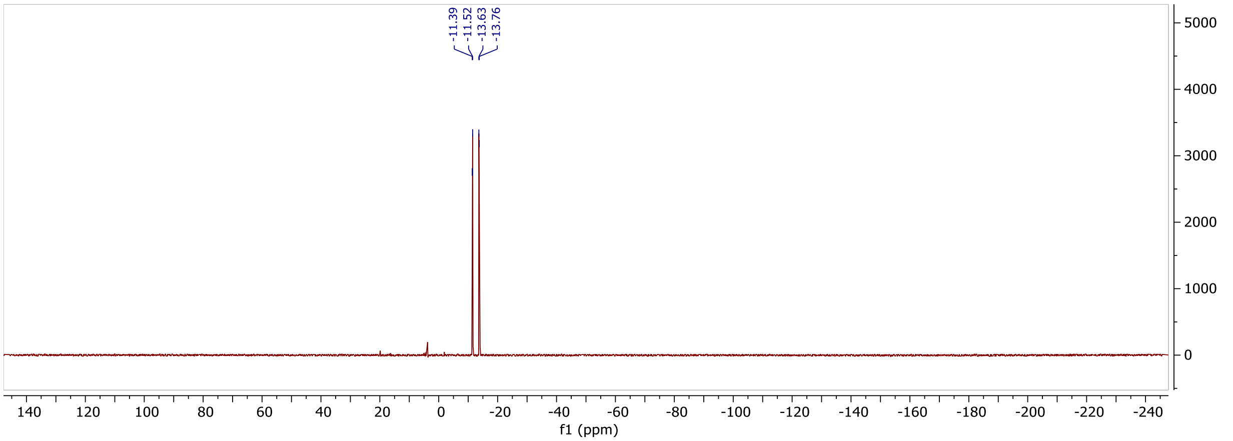

GDP-β-L-Rhamnose (13)

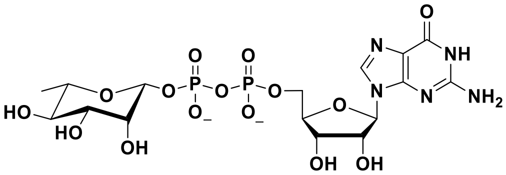

^{1}H NMR

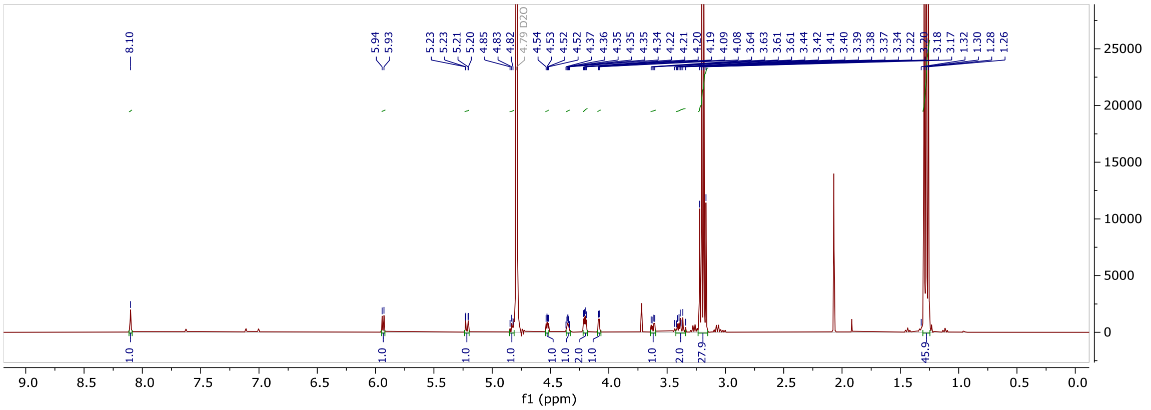

^{13}C NMR

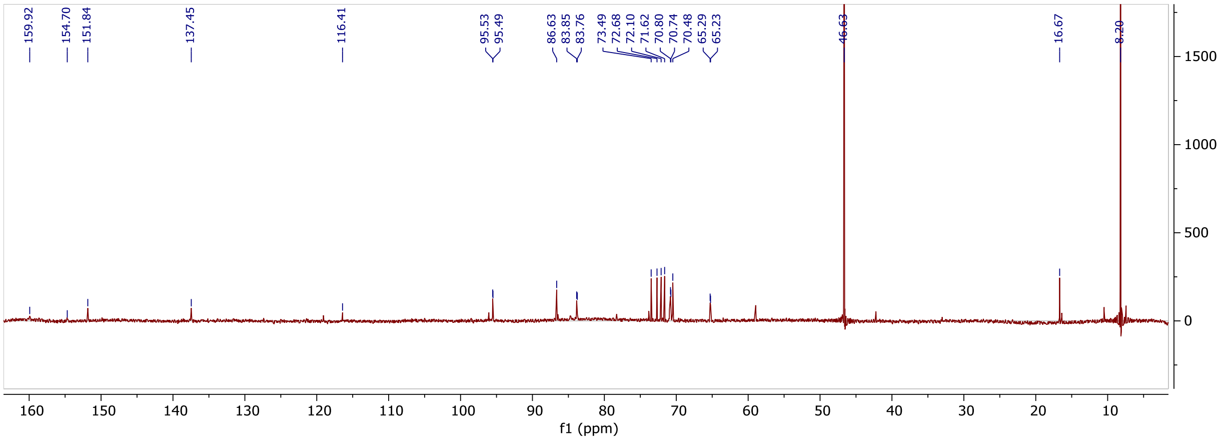

^{31}P NMR

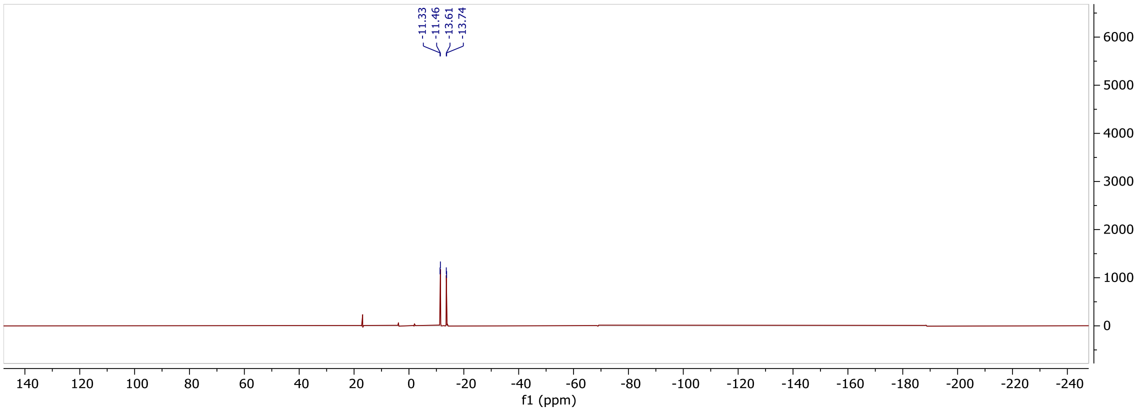

4 Experimental Biochemical Methods and Data.

Cloning of expression vector for Mtb RmlA overexpression in *E. coli*. *M. tuberculosis rmlA* was codon optimized for *E. coli* expression as a gene fragment obtained from Twist Bioscience, which used as a template for plasmid construction by overlap extension PCR[7] into a pET28b vector using the primers shown in **Table S3**. PCR insertion products were DpnI treated prior to PCR purification (Qiagen), and transformation into Mach1 competent cells (Invitrogen). Following sequence confirmation by DNA sequencing (Genewiz), plasmids (**Table S2**) were transformed into indicated competent cells (**Table S4**) for overexpression.

Overexpression and purification of diverse bacterial RmlA proteins from *E. coli*. Overexpression and purification of C-term His_6-tagged SALTY RmlA and mutants, C-term His_6-tagged *P. aeruginosa* RmlA, and His-SUMO *E. coli* RmlA proteins were carried out following a published protocol.[8]

For C-term His_6-tagged *M. tuberculosis* RmlA, protein was overexpressed in BL21 cells (Novagen) in Luria-Bertani (LB) medium supplemented with 25 μg/mL kanamycin containing corresponding plasmid (**Table S2**). Overnight cultures (5 mL) were used to inoculate 500 mL of LB medium (1:100) supplemented with 25 μg/mL kanamycin and grown to late log-phase (OD_{600}= 0.9, optical density at 600 nm) with shaking (37°C, 200 RPM). Cultures were then induced with 1 mM isopropyl β-D-1-thiogalactopyranoside (IPTG) for 20 hr (21°C, 200 RPM). Cells were then harvested by centrifugation (1857 x g, 20 min, 4 °C).

For C-term His_6-tagged *M. tuberculosis* RmlA purification, pellets from 500 mL of culture were each resuspended on ice with 25 mL of lysis buffer (50 mM sodium phosphate, pH 8.0, 300 mM NaCl, 10% glycerol, 20 mM imidazole). Cells were rocked for 30 min at 4 °C and were sonicated (Fisherbrand, 5 min, 30 sec on/30 sec off, 50% amplitude, twice with cooling between runs). Cellular debris was cleared by centrifugation (Beckman Coulter Allegra X-15R at 10,956 x g for 30 min at 4 °C). For each, supernatant was added to 3 mL of pre-washed/equilibrated Ni-NTA resin (Qiagen) and rocked for 1 hr at 4 °C. The column was washed with 2 column volumes (CVs) of 25 mM imidazole in lysis buffer. The targeted His-tagged proteins were eluted with 1 CV of elution buffer (250 mM imidazole in lysis buffer). Eluted fractions were concentrated using an Amicon Ultra Centrifugal Filter Device (Millipore, 10 kD molecular weight cut off (MWCO)) by centrifugation (1857 x g, 4 °C) and buffer exchanged into 10 mM tris(hydroxylmethyl)aminomethane hydrochloride (Tris-HCl), pH 8.0, 20 mM NaCl, and 10 % glycerol.

All protein purifications were performed at 4 °C. The concentration of each purified protein was determined by the DC protein assay (Bio-Rad) using bovine serum albumin (BSA) as a standard. Proteins were aliquoted, flash frozen with $N_{2(l)}$, and stored at -80 °C.

Analysis bacterial RmlA inhibition by synthetic NDP-sugars. Activities of RmlA from different species were measured using a reported coupled malachite green assay.[8, 13] Reactions were conducted with 12.5 nM SALTY, *E.coli* and *P.aeruginosa* RmlA and 25 nM of *M. tuberculosis* RmlA with 0.008 mg/mL inorganic pyrophosphatase (PPiase) in reaction buffer (35 mM Tris-HCl, pH 7.5, 2.2 mM $MgCl_2$) [8, 14] with 100 μM each dTTP and α-Glc-1P at 25 °C in a final volume of 30 μL. The linear phase was determined as shown in **Figure S6D**.

For each dilution series, the compounds were diluted in water and added to reactions with final concentrations of: 1000 μM, 500 μM, 250 μM, 175 μM, 100 μM, 75 μM, 50 μM, 25 μM, 10 μM, 2.5 μM, 1 μM, and 0 μM (control reaction with no inhibitors added; water was used). In a typical reaction, RmlA and PPiase were pre-incubated in reaction buffer at 25 °C for 5 min with and without inhibitors, enzymatic reactions were then initiated with the addition of 100 μM substrates, yielding a final volume of 30 μL. At t = 6 min post-initiation of each reaction, 30 μL of 0.05% formic acid was added to quench the reaction. For each set of reactions, reactions that only contained substrates and inhibitors at each concentration (12 total) in reaction buffer were prepared and treated the same as the reaction described above except no enzyme

was added. Reactions were analyzed using malachite green as previously published with slight modification.[8] Briefly, for the coupled malachite green colorimetric assay, a calibration curve was first generated via two-fold serial dilution of monophosphate (1 mM monophosphate (Pi) to 0.976 μM Pi) using the reaction buffer mentioned above ; 50 μL of reaction buffer was then added to a blank well. Each quenched reaction (50 μL each) was then added to a 96-well clear plate (Greiner) with 50 μL of premixed malachite green reagents. The premixed malachite green reagent contained 0.0812% malachite green, 2.32% poly(vinyl alcohol), 5.72% ammonium molybdate in 6 N HCl, and water (2:1:1:2, volume ratio respectively). Reagents were preincubated at 25 °C for t = 10−15 min until the color appeared gold/yellow prior to addition to reactions. After addition of reagents, the plate was incubated at 25 °C for t = 30 min and subsequently analyzed using a plate reader (Molecular Devices, SpectraMax iD5) by measuring absorbance at 630 nm. Data was exported from SoftMax Pro 7.1 software. The concentration of PPi was calculated using a monophosphate standard curve for each experiment and graphed in GraphPad Prism 9. To generate the standard curve, the absorbance of water was subtracted from the obtained absorbance, followed by normalizing the absorbance value to buffer well. The resulting delta absorbance was plotted against the corresponding concentration of Pi to generate the calibration curve. A linear fit was found between 0 and 62.5 μM Pi. To calculate the concentration of PPi produced in each reaction, normalized absorbance values were compared to the standard curve. Relative percent activity of each reaction was calculated by normalizing each to the reaction without added inhibitor, which was set to 100% activity.

In order to calculate the half-maximal inhibitory concentrations (IC50) of compounds against each RmlA protein, % activity versus log(10) of inhibitor concentration (μM) data were analyzed in GraphPad Prism 9 using log(inhibitor) vs. normalized response – variable slope with the equation of Y = 100/(1+10^((LogIC50-X)*HillSlope))). Variable slope was used because each RmlA protein demonstrated a different level of cooperativity with added ligands.

Kinetic analyses of wild-type and mutant RmlA constructs. Steady-state kinetic parameters of natural substrates in the absence of inhibitor were obtained by fixing glucose-1-phosphate at 100 μM and titrating the substrate dTTP. For dTTP, eight concentrations (0–800 μM) were assayed in three replica experiments. For each reaction, 12.5 nM of RmlA enzyme and 0.008 mg/mL PPiase were pre-incubated at 25 °C (t = 5 min) in buffer (35 mM Tris-HCl pH 7.5, 2.2 mM $MgCl_2$). Reaction was subsequently initiated by adding corresponding substrates (reaction volume 30 μL), incubated at 25 °C (t = 5 min), and quenched using 30 μL of 0.05% formic acid.

For kinetic analysis in the presence of inhibitor dTDP-β-L-Rha, reactions were performed following the protocol described above with slight modification. For each reaction, 100 μM or 200 μM of dTDP-β-L-Rha was added to 12.5 nM RmlA enzyme and 0.008 mg/mL PPiase in buffer (35 mM Tris-HCl pH 7.5, 2.2 mM $MgCl_2$), which were pre-incubated at 25 °C (t = 5 min). Reaction was subsequently initiated by adding corresponding substrates (reaction volume 30 μL), incubated at 25 °C (t = 5 min), and quenched using 30 μL of 0.05% formic acid. The reactions were subsequently analyzed using the coupled malachite green assay described above.

Kinetic parameters of RmlA constructs were obtained using Prism with the following equation (“determine k_{cat}”): $Y = Et * k_{cat} * X / (K_M + X)$ (X is the substrate concentration in μM; Y is the enzyme velocity in μM/min; Et is enzyme concentration in μM).

Analysis of RmlA-mediated activation of S-1Ps by analytical HPLC. For analysis of RmlA activity, reactions were carried out by following a published protocol with slight modifications.[8] Reactions were performed in a final volume of 30 μL containing 5 μM mutant or wild-type RmlA, 0.008 mg/mL PPiase, 2

mM dTTP, and 2 mM S-1P in buffer (100 mM 3-(*N*-morpholino)-propanesulfonic acid (MOPS), pH 7.5, and 7.5 mM $MgCl_2$) based on conditions from a previous report.[15] Reactions were incubated at 37 °C for 6 hr (or indicated time points) and quenched by adding 30 μL of 0.05% formic acid. For analysis of synthetic S-1P activation at high concentrations, 10 mM S-1P was used and reactions were incubated at 37 °C for 24 hr. Subsequently, 1 U of 1 U/μL Shrimp Alkaline Phosphatase (rSAP) was added to the reaction, which was then incubated at 37 °C for 2 hr prior to quenching with 30 μL of 0.05% formic acid. Analytical HPLC analysis was performed on a Thermo Scientific Dionex UltiMate 3000 UHPLC+ with a Phenomenex 5 μm, 4.6 x 150 mm, NX-C18, 110 Å Gemini column. Each sample (15 μL) was injected and eluted using 50 mM triethylammonium bicarbonate (TEAB, Buffer A) and acetonitrile (Buffer B) over a linear gradient of 0-5% Buffer B or 0-10% Buffer B at a flow rate of 1.0 mL/min for t = 16 min using 254 nm wavelength for detection. To obtain the percent NDP-sugar, the area of peak representing NDP-sugar was normalized to the total area of peaks containing nucleotides (NTP, NDP, NMP, and NDP-sugar) or nucleoside if rSAP was used, which was analyzed with Chromeleon software (Thermo Scientific).

LCMS or HRMS analysis of RmlA reactions containing S-1P and (d)NTPs. Reactions were carried out under the reaction conditions mentioned above. For LCMS analysis, quenched reactions (50 μL) were loaded into 96-well noncoated polypropylene microplates (Thermo Scientific). Analysis of standard NDP-sugars and RmlA enzymatic reactions was performed on a Thermo Scientific LCMS-TQ Fortis system with an electrospray (ESI) ionization source equipped with an autosampler through a Phenomenex 5 μm, 4.6 × 150 mm, NX-C18, 110 Å Gemini column. Each sample (15 μL) was injected and eluted using 50 mM TEAB buffer (Buffer A) and acetonitrile (Buffer B) using a linear gradient 0-5% Buffer B at a flow rate of 0.5 mL/min for t = 16 min with detection at 254 nm. For extracted ion chromatography (EIC) analysis, SIM Q1 was employed with a selected center mass (547, 549, and 563 m/*z*) at a scan rate of 250 Da/sec and a scan width of 10 m/*z* in the negative ion mode. For general mass detection of compounds during LC analysis, full Scan Q1 was used with scan range from 250 to 1000 m/*z* at a scan rate of 1000 Da/sec in the negative ion mode.

For HRMS analysis of select NDP-sugars produced via chemoenzymatic reactions, HPLC fractions representing indicated peaks were lyophilized, resuspended in HPLC-grade water and analyzed by using an Agilent 6224 Accurate-Mass time-of-flight LC/MS as previously described.[8]

References:

1. Tsukamoto, H.; Kahne, D., *N*-Methylimidazolium chloride-catalyzed pyrophosphate formation: Application to the synthesis of Lipid I and NDP-sugar donors. *Bioorg. Med. Chem. Lett.* **2011,** *21,* 5050-5053.
2. Höfle, G.; Steglich, W.; Vorbrüggen, H., 4-Dialkylaminopyridines as Highly Active Acylation Catalysts. [New synthetic method (25)]. *Angew. Chem. Int. Ed. Eng.* **1978,** *17,* 569-583.
3. Sun, Q.; Li, X.; Sun, J.; Gong, S.; Liu, G.; Liu, G., An improved P(V)-N activation strategy for the synthesis of nucleoside diphosphate 6-deoxy-L-sugars. *Tetrahedron* **2014,** *70,* 294-300.
4. Demkiw, K. M.; Hu, C. T.; Woerpel, K. A., Hyperconjugative Interactions of the Carbon–Halogen Bond that Influence the Geometry of Cyclic α-Haloacetals. *J. Org. Chem.* **2022,** *87,* 5315-5327.
5. *Schrödinger Release 2023-1: Glide*, Schrödinger, LLC: New York, NY, 2021.
6. Muona, M.; Aranko, A. S.; Iwai, H., Segmental isotopic labelling of a multidomain protein by protein ligation by protein trans-splicing. *Chembiochem* **2008,** *9,* 2958-61.
7. Bryksin, A. V.; Matsumura, I., Overlap extension PCR cloning: a simple and reliable way to create recombinant plasmids. *BioTechniques* **2010,** *48,* 463-465.

8. Zheng, M.; Zheng, M.; Lupoli, T. J., Expanding the Substrate Scope of a Bacterial Nucleotidyltransferase via Allosteric Mutations. *ACS Infect. Dis.* **2022,** *8,* 2035-2044.
9. Sabesan, S.; Neira, S., Synthesis of glycosyl phosphates and azides. *Carbohydr. Res.* **1992,** *223,* 169-185.
10. Danieli, E.; Proietti, D.; Brogioni, G.; Romano, M. R.; Cappelletti, E.; Tontini, M.; Berti, F.; Lay, L.; Costantino, P.; Adamo, R., Synthesis of *Staphylococcus aureus* type 5 capsular polysaccharide repeating unit using novel L-FucNAc and D-FucNAc synthons and immunochemical evaluation. *Bioorg. Med. Chem.* **2012,** *20,* 6403-6415.
11. Burgoyne, A. R.; Kaschula, C. H.; Parker, M. I.; Smith, G. S., Tripodal Half-Sandwich Rhodium and Iridium Complexes Containing Sulfonate and Pyridinyl Entities as Antitumor Agents. *Eur. J. Inorg. Chem.* **2017,** *2017,* 5379-5386.
12. Munte, C. E.; Karl, M.; Kauter, W.; Eberlein, L.; Pham, T.-V.; Erlach, M. B.; Kast, S. M.; Kremer, W.; Kalbitzer, H. R., High pressure response of ^{1}H NMR chemical shifts of purine nucleotides. *Biophys. Chem.* **2019,** *254,* 106261.
13. Sha, S.; Zhou, Y.; Xin, Y.; Ma, Y., Development of a colorimetric assay and kinetic analysis for *Mycobacterium tuberculosis* D-glucose-1-phosphate thymidylyltransferase. *J. Biomol. Screen.* **2012,** *17,* 252-7.
14. Timmons, S. C.; Mosher, R. H.; Knowles, S. A.; Jakeman, D. L., Exploiting Nucleotidylyltransferases To Prepare Sugar Nucleotides. *Org. Lett.* **2007,** *9,* 857-860.
15. Moretti, R.; Chang, A.; Peltier-Pain, P.; Bingman, C. A.; Phillips, G. N., Jr.; Thorson, J. S., Expanding the nucleotide and sugar 1-phosphate promiscuity of nucleotidyltransferase RmlA via directed evolution. *J. Biol. Chem.* **2011,** *286,* 13235-43.
